# Assessing Current Fish Diversity in the Yellow River Basin by Integrating Large‐Scale Barcoding and Morphological Data

**DOI:** 10.1002/ece3.72617

**Published:** 2025-12-22

**Authors:** Chuanjiang Zhou, Ke Ma, Tai Wang, Yongtao Tang, Meng Zhang, Jinhui Yu, Ruyao Liu, Qiqi Ding, Shangjin Qiu, Yikai Li, Zhigang Ma, Guoxing Nie

**Affiliations:** ^1^ College of Life Sciences Henan Normal University Xinxiang People's Republic of China; ^2^ College of Fisheries, Engineering Technology Research Center of Henan Province for Aquatic Animal Cultivation Henan Normal University Xinxiang People's Republic of China; ^3^ Gansu Key Laboratory of Cold Water Fishes Germplasm Resources and Genetics Breeding Gansu Fisheries Research Institute Lanzhou People's Republic of China

**Keywords:** conservation, cryptic diversity, DNA barcode, species delimitation, species polyphyly

## Abstract

In recent years, fish populations in the Yellow River (YR) basin have declined sharply due to human activities. Some studies even suggest that nearly half of the native fish species in the YR have disappeared. There are also significant differences among the literature on the systematic discussion of fish species in the Yellow River Basin. The construction of a standardized and complete DNA barcode database for YR fish is urgently needed. In this study, 127 sampling points were sampled in the YR basin from 2013 to 2023, and a DNA barcode database of 3011 sequences and 110 species was constructed. A total of 124 operable taxonomic units (OTUs) were identified via four sequence‐based species classification methods. The barcode and morphological results of 84 (77.1%) species remained consistent. Some species cannot be described in the literature and are assigned to unique OTUs, and some widely distributed species are also assigned multiple OTUs. These findings indicate that the fish diversity in the YR basin has been severely underestimated, and more detailed investigations and further research are needed. This study constructed the first fish DNA barcode database in the YR basin and provided new insights for the study of fish diversity in the region.

## Graphical abstract

In this study, 127 sampling points were sampled in the Yellow River Basin, and a DNA barcode database of 3011 sequences and 110 species was constructed. We found that diversity of fish species in the Yellow River Basin has been seriously underestimated.

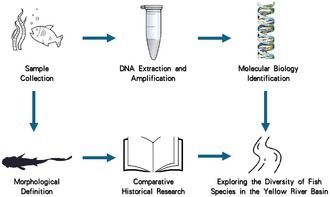

## 1 Introduction

The Yellow River (YR) originates in the Gezigeya Basin in the northern Bayankala Mountains and flows across half of China from west to east into the Bohai Sea. It is the fifth‐longest river in the world, covering a distance of 5687 km and draining an area of 813,100 km^2^; it has 4157 tributaries exceeding 50 km^2^ (J. Cai 2017). The YR spans four geomorphic units, including the Qinghai‐Tibet Plateau, the Inner Mongolian Plateau, the Loess Plateau, and the North China Plain. Its diverse hydrological environment, complex trunk and tributary patterns, and extremely high sediment concentration have led to many endemic fish species in the basin (He and He 2012; Miao et al. 2011; Zhao et al. 2020). However, in recent decades, human activities have considerably changed the habitat of the YR basin. Although the YR Basin accounts for 2% of China's water resources, it accounts for about 6% of the country's wastewater and 7% of its chemical oxygen demand emissions. Some watersheds are severely polluted, with 14.7% of the total threatened fish species (Ministry of Ecology and Environment 2018). The construction of 33 water conservancy projects on the YR has fragmented the YR and significantly altered its hydrological environment (Xie et al. 2018). Owing to the high sediment content of the YR, massive amounts of sediment can accumulate in water conservancy facilities. The government releases half of the annual amount of sediment from the Yellow River into the ocean within 15–20 days by creating artificial floods, leading to natural seasonal changes in the downstream water and sediment discharge and complete changes in the properties of the sediment (Bi et al. 2014; Wang et al. 2010). At the end of the last century, multiple interruptions have occurred in the mainstream of the YR. Humans have caused pollution of the YR, fragmentation of the river, changes in hydrological patterns, and river interruption; these problems have resulted in significant habitat loss of the fish in this river.

According to the “Fishes of the Yellow River” (Li 2017), 183 fish and 147 pure freshwater fish were recorded in the YR. Due to numerous revisions to species classifications, the exact number of fish species still needs to be verified. However, recent studies on the entire YR basin revealed that from 2000 to 2019, only 112 fish species were reported, and 48.9% of the local fish species in the entire basin went extinct (Wang, Chen, et al. 2021). Most of these studies involving the entire watershed used historical data, and their sampling points could not cover the entire watershed (Jia et al. 2020; Wang, Yan, et al. 2021; Xie et al. 2018). Early studies on fish species in the YR focused mostly on certain river sections and specific groups (Cui 2013; Hu 2022). Other studies were conducted based on earlier findings and integrated fragmented data, potentially underestimating the number of fish species in the YR. A study on the main stream of the YR sampling involved only 18 sampling sites and 54 species of fish (Ru et al. 2010). In the last few decades, the number of fish resources in the YR basin has decreased considerably. Researchers are unable to elucidate the current situation of YR fish resources. Given the government's implementation of a fishing ban policy in the YR, the condition of fish resources in the YR needs to be evaluated, and targeted protection policies formulated.

Owing to the phenotypic diversity and differences in life stages among fish species, traditional species identification methods require researchers to be highly knowledgeable (Chantangsi et al. 2007; Park et al. 2019). However, the number of traditional taxonomists is decreasing worldwide (Antil et al. 2023). The application and development of the DNA barcoding technique can alleviate this problem. When identifying species, a barcode is used for preliminary screening of sequences, and then, a unified diagnosis of problematic species is performed. This method greatly improves the identification efficiency (Hubert and Hanner 2015). DNA barcodes, which use a standardized DNA region as a label for rapid, accurate species identification, were first proposed in 1993 (Arnot 1993; Hubert and Gregory 2005). In fish, the standard barcode gene is the region that encodes mitochondrial cytochrome‐c oxidase 1 (COI) (Hebert et al. 2003). The successful application of DNA barcode technology worldwide has shown that it can be effectively used for identifying freshwater fish; however, it cannot be used to detect or discover hidden diversity (Chen et al. 2015; Hubert et al. 2008; Papa et al. 2021; Sonet et al. 2019; Tsoupas et al. 2022; Ward et al. 2005). The effectiveness of this method in identifying fish species in the YR basin needs to be assessed to evaluate its impact on subsequent protection programs. The unique habitat and numerous endemic species found in the YR might also hinder the application of DNA barcodes.

We constructed a YR DNA barcode database containing 3011 sequences and 110 species using 2991 newly generated DNA barcode records and 20 sequences from the NCBI database. We discuss the reliability of the database, compare it with historical lists, and finally elaborate on the current status of fish species diversity in the Yellow River Basin.

## 2 Materials and Methods

### 2.1 Sample Collection and Morphological Identification

**FIGURE 1:**
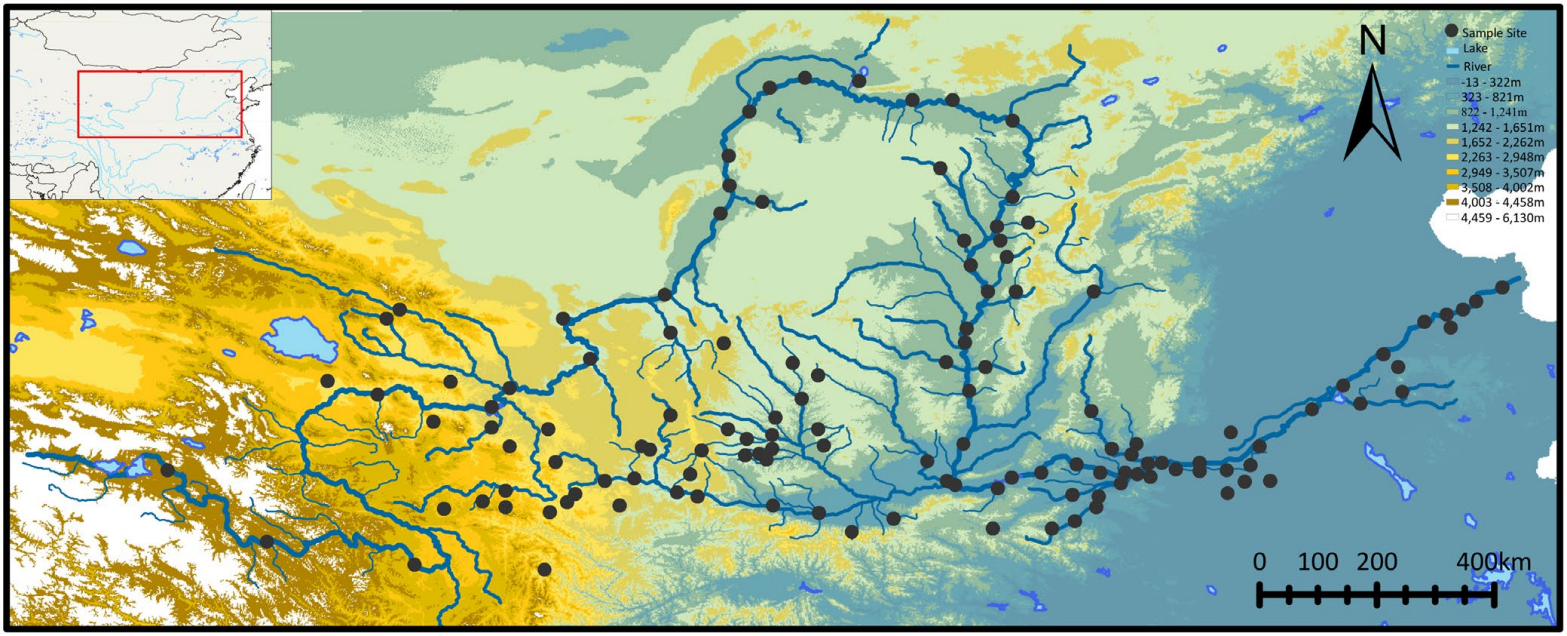
The collection sites involved in this study. Details of the 127 sites and collected samples are provided in Table S1.

From 2013 to 2023, 2990 fish samples were obtained from 127 sampling sites covering the entire YR basin (Figure 1), and only samples of *Coreius septentrionalis* were collected in 1993. The fish samples were identified based on the information presented in “Fishes of the Yellow River” (Li 2017), “Fauna Sinica” (Chen 1998), and “The Loaches of the Subfamily Nemacheilinae in China” (Zhu 1989). Species that did not match the description in published studies and could not be identified were classified as unknown species. The tissue used for DNA extraction was preserved in 95% ethanol. Voucher samples were preserved in 10% formaldehyde solution. The authors declare that all experimental protocols involving animals were approved by the Animal Research and Ethics Committee of Henan Normal University and the Laboratory Animal Guidelines for the Ethical Review of Animal Welfare (GB/T 35892‐2018), and that the research was conducted in accordance with the research standards.

**FIGURE 2:**
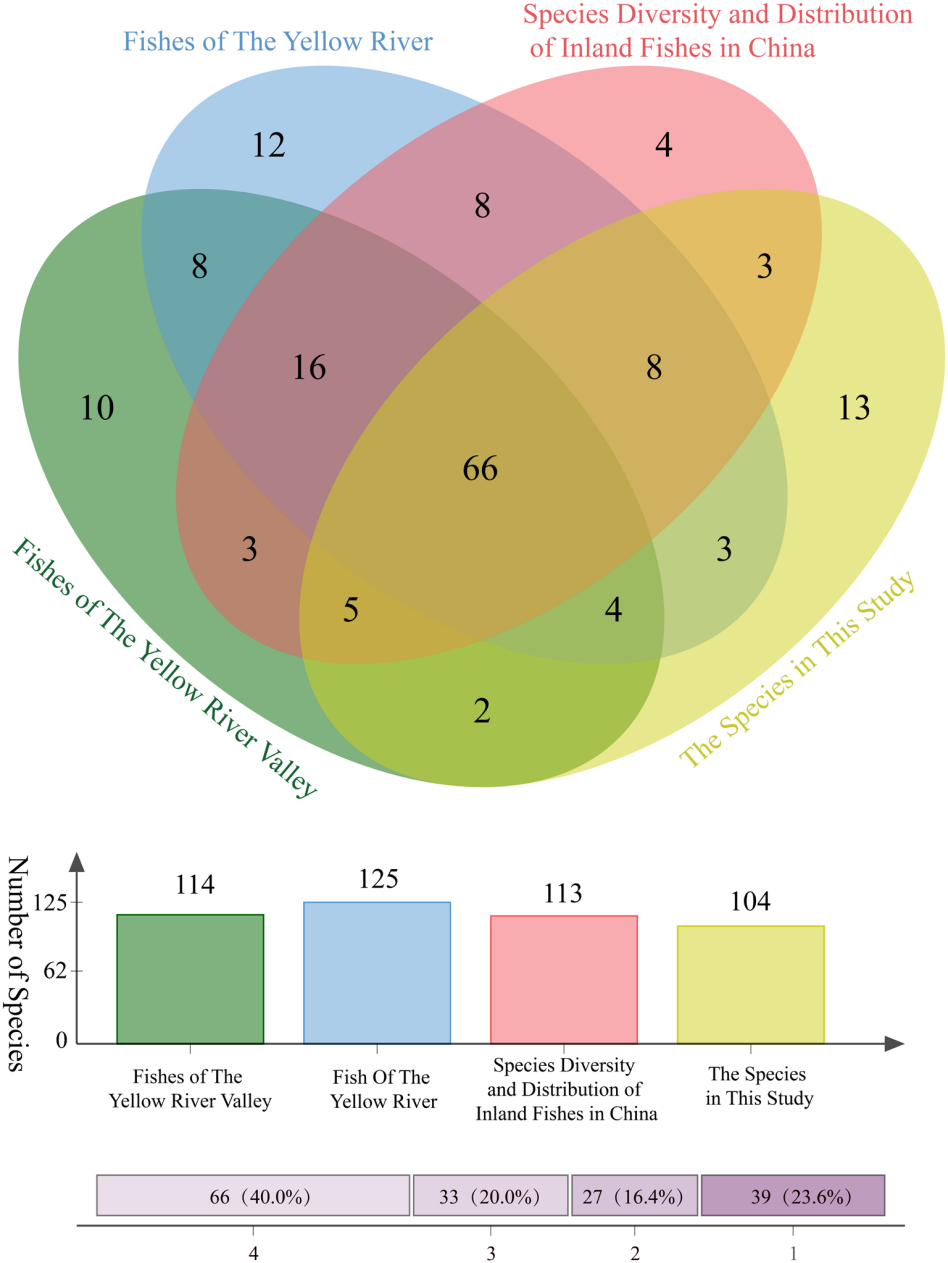
Differences in the records of freshwater fish in the YR Basin reported in different studies.

We also integrated the lists of YR fish from the “Fishes of the Yellow River” (Li 2017), “Species Diversity and Distribution of Inland Fish in China” (Zhang and Zhao 2016) and “Fish of the Yellow River Valley” (W. Cai 2013), excluding species with changed taxonomy information or without preserved specimens. The differences in YR fish species were compared between different lists, and the percentage of overlapping species in each list was calculated (Table S5). To visually display the results, we used Jvenn on the web interface (https://jvenn.toulouse.inrae.fr/app/example.html) to construct Venn diagrams of species differences between different lists (Figure 2) (Bardou et al. 2014).

### 2.2 Obtaining DNA Sequences

All the fish DNA was extracted from the fins and muscles using the standard salt extraction protocol (Reisfeld et al. 1971). Agarose gel electrophoresis was performed (using a 1% agarose gel) to assess DNA integrity, and a Nanodrop2000 was used to estimate the concentration of the extracted DNA. We used the FishF1/FishR1 (Ward et al. 2005) and Fish‐F2/Fish‐R2 (Liu et al. 2020) fish universal primers for polymerase chain reaction (PCR) amplification. The sequences of primers used were as follows: FishF1‐5′ TCAACCAACCACAAAGACATTGGCAC3′ and FishR1‐5′ TAGACTTCTGGGT GGCCAAAGAATCA3′, Fish‐F2: 5′ CATCCTACCTGTGGCAATCAC‐3′, Fish‐R2: 5′ TGGGCTCAG ACAATAAATCCT‐3′, and the expected size of the primers was 650 bp. The polymerase chain reaction (PCR) mixture (25 μL) contained 13 μL of 2X San Taq PCR Master Mix (Sangon Biotech, Shanghai, China), 10 μL of sterilized ultrapure water, 0.5 μL of each primer, and 1.0 μL of the DNA template (50–200 ng/μL). The PCR amplification conditions were as follows: 94°C for 5 min; 35 cycles at 94°C for 30 s, 50°C–58°C for 30 s, and 7°C for 1 min; and a final extension at 72°C for 10 min. The PCR products were detected via gel electrophoresis via a 1% agarose gel. The PCR products that met the requirements were selected by Sangon Biotech Company (Shanghai, China) via an Applied Biosystems 3730XL sequencer, and BigDye Terminator v3.1 was used for bidirectional sequencing. As the *C. septentrionalis* sample was preserved in formaldehyde, its DNA was extracted following a method described in another study (Yu et al. 2022). The sequences were checked and assembled using the SEQMEN procedure in the DNASTAR Lasergene package (Burland 2000). We used MEGA 11.0 (Tamura et al. 2021) to align and trim the consensus sequences to 624 bp and further translated them into amino acids to check for potential stop codons. The sequence data were submitted to BOLD (project YRFIR “Yellow River Fish Identify Research”).

### 2.3 Analysis of Genetic Distance

We used the R package Ape 5.7 (Paradis and Schliep 2019) to calculate the Kimura two‐parameter (K2P) (Kimura 1980) pairwise genetic distances. To check the presence of barcode gaps (Meyer and Paulay 2005), we used the matrix of pairwise K2P genetic distance in the R package Spider 1.5 (Brown et al. 2012) to calculate the maximum intraspecies genetic distance and the minimum interspecies genetic distance. Then, we calculated their ratio, which was defined as the taxonomic resolution ratio (TRR) (Matzen da Silva et al. 2011). Finally, we used a scatter plot to visually represent the results. A phylogenetic tree was constructed in the BOLD system using the neighbor‐joining method and the K2P distance to assess genetic distance and DNA barcodes (Figure S1).

### 2.4 Species Delimitation

Results from sequence‐based and routine specimen identification are based on different methods, and species associated with these concepts within the same framework should be distinguished (Hubert and Hanner 2015). Therefore, we defined species based on DNA sequence recognition as operational taxonomic units (OTUs), which are diagnosable molecular lineages (Avise 1994; Moritz 1994; Vogler and DeSalle 1994). We used four species identification methods, namely, refined single linkage (RESL) (Ratnasingham and Hebert 2013), automatic barcode gap discovery (ABGD) (Puillandre et al. 2012), general mixed Yule coalescent (GMYC) (Fujisawa and Barraclough 2013), and the multirate Poisson tree process (mPTP) (Kapli et al. 2017). A final delimitation scheme was established based on a 50% consensus among methods. The RESL analysis was performed through the FUSTER SEQUENCE function in the workbench of the BOLD system. The ABGD (Puillandre et al. 2012) analysis used the default value for the relative gap width (*X* = 1.5) and K2P genetic distance. We constructed a maximum likelihood (ML) tree using the default parameters of RAxML (Stamatakis 2006) on the basis of the GTR + I + Γ model for further analysis of the mPTP. The fully resolved, ultrametric gene tree required for the GMYC analysis was constructed via the BEAST (Bouckaert et al. 2019) series software. First, ALTER (Glez‐Pena et al. 2010) was used to fold the duplicated sequence into haplotypes. Two Markov chains of 50 million each were run independently using a strict‐clock model based on a canonical 1% genetic divergence per million years and a GTR + I + Γ substitution model (Bermingham et al. 1997). After an initial burn period of 10 million, trees were sampled every 5000 states, and the two runs were combined using LogCombiner 2.7.3 (Bouckaert et al. 2019). Finally, we used TreeAnnotator 2.7.3 (Bouckaert et al. 2019) to establish the maximum credibility tree. Tracer 1.7.2 was used to evaluate the convergence of the system tree, and all ESS values were greater than 200, which indicates that the system tree converged. To visualize the relationships between species that shared haplotypes, we used Tamura‐Nei's consensus sequence (TCS) network method to construct a haplotype network diagram by POPART 1.7 (Leigh et al. 2015).

## 3 Results

We established a barcode database of 3011 sequences, including 2991 newly obtained sequences from 127 sampling sites and 20 sequences downloaded from NCBI (Table S1). This database included 11 orders, 21 families, 65 genera, and 110 species, which consisted of 81 species in Cypriniformes, 10 species in Perciformes, 7 species in Siluriformes, 4 species in Salmoniformes, 2 species in Cyprinodontiformes, and 1 species in Anguilliformes, Synbranchiformes, Beloniformes, Clupeiformes, Mugiliformes, and Pleuronectiformes (Table S2). Additionally, we discovered eight unknown species, some of which might be new reports. Except for the shorter sequences extracted from the formaldehyde samples of *C. septentrionalis* (400 bp), all the sequences were at least 624 bp, and no termination codons were found. Given that the *C. septentrionalis* sequences were less than 624 bp, the data for this species were not used in the subsequent analysis. The number of sequences in a single species ranged from 1 to 218. The number of sequences for one species exceeded 200 because that species was temporarily indistinguishable morphologically but showed significant molecular differences, leading to the use of more sequences. Single species were found at up to 33 sampling points in seven provinces (Table S1).

We corrected the taxonomic information for freshwater fish reported in three studies using Eschmeyer's catalog, and then removed species without preserved samples. The final list included 125 species, 114 species, 113 species, and 104 species of freshwater fish, as recorded in the “Fishes of the Yellow River” (Li 2017), “Species Diversity and Distribution of Inland Fish in China” (Zhang and Zhao 2016), “Fish of the Yellow River Valley” (W. Cai 2013), and this study. Based on the above four lists, 165 freshwater fish species were found in the YR. There were only 66 species (40% of the total) described in all the lists, but 39 species (23.64% of the total) were described in only a single list (Table S5), indicating that there were still differences in the descriptions of fish in the YR Basin. The number of freshwater fish species identified in this list accounts for 63.03% of the total reported species in the YR basin.

**FIGURE 3:**
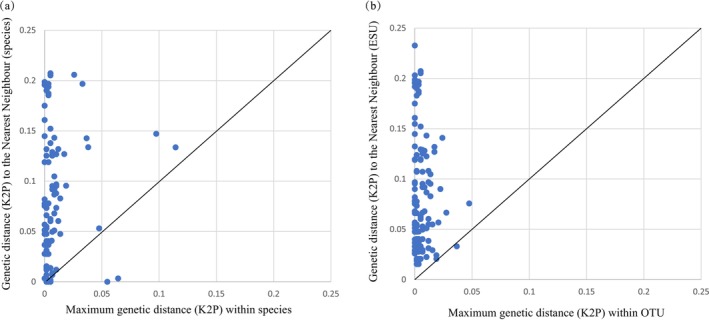
(a) Relationships between the maximum intraspecific and nearest neighbor K2P genetic distances among species. (b) Relationships between the maximum intraspecific and nearest neighbor K2P genetic distances when accounting for multiple OTUs within species.

The results of the genetic distance analysis revealed that the maximum intraspecific distance was 0.114, and the nearest‐neighbor distance was 0.233. The average value of the maximum intraspecific distance was 0.009, and the average value of the nearest neighbor distance was 0.088. On average, the nearest‐neighbor distance was 9.8‐fold greater than the maximum intraspecific distance, with an index ratio ranging between 0 and 114.8. We found that 14 fish had a minimum interspecies genetic distance of less than 1%, and 9 fish had a maximum intraspecies genetic distance of more than 2%. The maximum intraspecific genetic distance of the eight species was greater than the minimum interspecific genetic distance. The minimum interspecific genetic distance for the five fish *Gymnocypris eckloni* , *Gymnocypris przewalskii* , *Triplophysa pappenheimi* , *Triplophysa siluroides* , and *Triplophysa minxianensis* was 0 (Table S4). The overlap of these two distributions confirmed the absence of barcode gaps (Figure 3).

**FIGURE 4:**
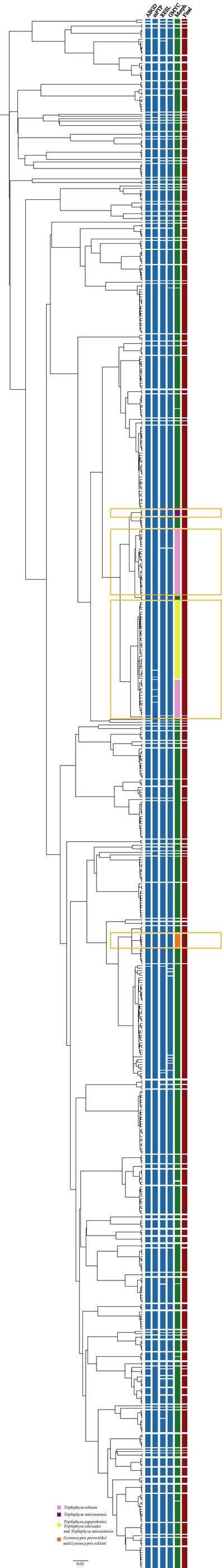
Bayesian phylogenetic tree of the 3010 DNA barcodes, species morphological diagnoses (green), and OTU delimitation schemes collected from the RESL, ABGD, mPTP, and GMYC algorithms (blue), including the consensus delimitation scheme (red). Some species are distinguished by other colors because of the significant differences in the results between the different methods. Detailed summary statistics are available in Table S3.

The four sequence‐based species delimitation methods produced different OTU counts, all of which exceeded the number of species identified morphologically. The results revealed that ABGD generated 124 OTUs, mPTP generated 117 OTUs, RESL generated 132 OTUs, and GMYC generated 136 OTUs. The consensus delimitation scheme yielded 124 OTUs (11 OTUs of unknown species), which differed from the method based on morphological recognition (Table S3). We found nine species with multiple OTUs, with a maximum of five OTUs per species; OTU sharing was observed in 16 species. In total, 25 species presented mixed genealogies (Figure 4). By plotting the maximum intraspecies and minimum interspecies genetic distances based on the final OTU definition scheme, we found that the separation between the two distributions improved, but there were missing barcode gaps (Figure 3).

**FIGURE 5:**
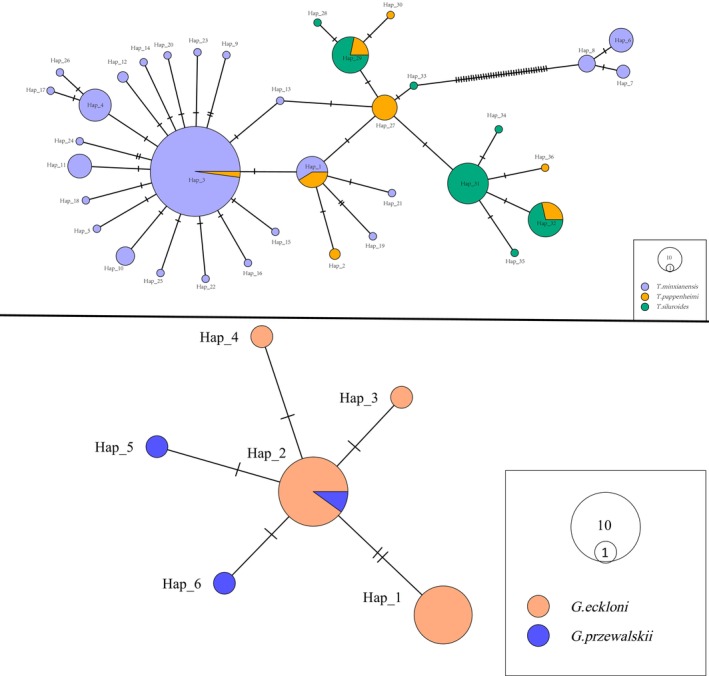
Haplotype networks reconstructed via Tamura‐Nei's consensus sequence (TCS) network method for *Gymnocypris eckloni* and *Gymnocypris przewalskii* . The haplotype networks of *Triplophysa minxianensis* , *Triplophysa pappenheimi* and *Triplophysa siluroides* are shown.

The haplotype analysis revealed that haplotype sharing occurred in three species of *Triplophysa* and two species of *Gymnocypris*. *T. pappenheimi* shares two haplotypes with *T. minxianensis* and *T. siluroides* (Figure 5). One haplotype was also shared between *G. przewalskii* and *G. eckloni* (Figure 5).

## 4 Discussion

The YR, as the fifth‐longest river in the world, has much less fish data in the NCBI and BOLD databases than other rivers of similar length. We believe that the economic backwardness of the YR basin is also an important reason for the lack of data (Perret et al. 2023). Previous research on the YR has focused mainly on investigating fish resources in certain sections of the river, with little involvement in molecular‐level research, and most related research has focused on hotspot areas (Jia et al. 2020; Wang, Chen, et al. 2021; Xie et al. 2018; Zhao et al. 2020). Biased data cannot be used to scientifically evaluate the status of fish species diversity in the YR. To address this limitation, we constructed a relatively reliable barcode database using dense sampling and a large dataset. From 2013 to 2023, we found 95 recorded indigenous fish species in the YR basin (Table S2). According to the results combined with those of other studies, the number of local species can exceed 110. Recent research on the YR has shown that only 80 or 72 species of native fish have been reported in the YR basin in the past 20 years (Wang, Chen, et al. 2021; Xie et al. 2018). Although the diversity of fish species in the YR is decreasing, more serious conclusions have been drawn due to data distortion issues. This study reported many species that have not been recorded in the YR basin over the past 20 years, including three fish species that are not listed in online databases, through large‐scale, long‐term, and multiple sampling in the Yellow River Basin. This study greatly fills the gap in fish diversity research in the YR basin. The number of invasive species surveyed in this study was lower than that reported in other studies, and most of them were small fish with no economic value. Economic farmed species such as *Oncorhynchus mykiss* and *Micropterus salmoides* were not found in this survey. This may be because the sampling points we selected are far from human settlements and there are fewer lakes and reservoirs.

The number of fish species per 10,000 km^2^ in the Huai River and Hai River is 5.91 and 3.13, respectively, whereas that in the YR, located between the two water systems, is only 1.95 (if calculated based on the list with the highest number of species, it would be 1.66) (Zhao et al. 2020). Even the number of freshwater fish in the Amur River basin, which has an average latitude 15° higher than that of the YR, is as high as 132, which is greater than that of any of the aforementioned lists of fish in the YR (He et al. 2020). These data indicate that the diversity of fish species in the YR basin may be severely underestimated. Nearly a quarter of the species in these four lists published in the past decade are described in only a single list, further supporting the above viewpoint (Figure 2). Therefore, we believe that comprehensive, sustained species surveys are needed in the YR basin to better understand the status of fish resources in the region.

In 2010, a study sampling the mainstream YR revealed only 54 fish species, but these findings may reflect the true distribution of fish in the YR (Ru et al. 2010). This study revealed that many species are distributed only in the clear water areas of the upper reaches of the YR tributary (Table S5). However, these species have small populations, and the number of preserved samples is low. In recent years, the deterioration of water quality has led to the decline and eventual disappearance of these fish species. This may lead to disputes over the records of these rare fish species in different lists. The four lists show that the unique species recorded in each study were mostly found in the watershed near their research unit. For example, the newly recorded species in this study were mostly found in the tributaries of the Henan section of the YR, whereas the newly recorded species in the “Fish of the Yellow River Valley” were mostly found in the tributaries of the Shanxi and Shaanxi sections of the YR. These findings are the result of years of local research by these teams and indicate that the existing survey methods in the YR basin have limitations. We believe that comprehensive and long‐term investigations of the YR need to focus on the upstream regions of some tributaries.

Among the 109 species recorded in this study (the *C. septentrionalis* sequence was short and was not analyzed), 84 (77.1%) matched the barcode clustering results. The consistency rate was mostly lower than that reported in many studies (April et al. 2011; Delrieu‐Trottin et al. 2019; Shen et al. 2019; Sholihah et al. 2020; Sonet et al. 2019). However, studies with samples collected from a wide geographic range and with close species relationships revealed that as distribution ranges increase, barcode recognition rates decrease, dropping to as low as 40% (Chen et al. 2022). In this study, *Rhodeus ocellatus* , *Onychostoma macrolepis* , *Abbottina rivularis* , *Rhynchocypris oxycephalus*, *Triplophysa robusta* , *T. minxianensis* , *Paramisgurnus dabryanus* , *Oryzias latipes* , *Monopterus albus* , and *Rhynchopyris oxychalus* occupied multiple OTUs, which might be due to their widespread sample distribution (Table 1). Among these species, *R. ocellatus* and *R. oxycephalus* are distributed in multiple tributaries of the YR. The fragmentation of their habitats further promoted species diversification, which might be why they evolved into multiple OTUs (Table 1) (Geiger et al. 2014). We also identified eight unknown species and distinguished some of them from existing species. Other morphological and molecular evidence will be added. The final number of OTUs exceeded the number of morphological species, and some unknown species were assigned to independent OTUs, which indicated that the barcodes could be used to identify hidden species (Durand et al. 2017; Hajibabaei et al. 2006; Winterbottom et al. 2014; Wu et al. 2023). These results also suggested that there was cryptic diversity among fish in the YR and that this diversity had previously been underestimated.

**TABLE 1:** List of the species with more than OTUs delimited and shared by multiple species including their maximum intraspecific and within OTU K2P genetic distances and K2P genetic distances to the nearest neighbor.

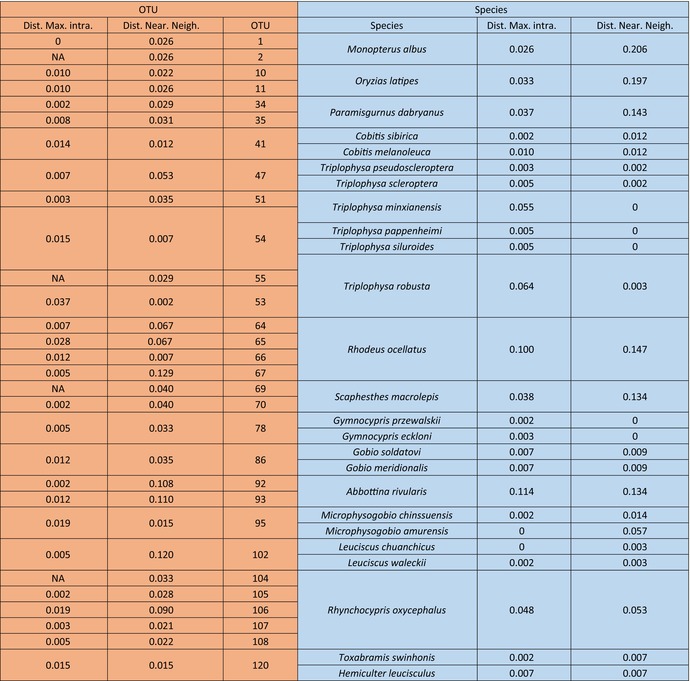

The most disorganized phylogenetic relationship in this study was that of *Triplophysa*, which is a unique group of fish that emerged after the uplift of the Qinghai‐Tibet Plateau (He et al. 2006). Owing to geological activities on the Qinghai‐Tibet Plateau, the connection and disconnection of water systems facilitated contact between different populations of *Triplophysa* in adjacent and different water systems, leading to interspecific hybridization (Favre et al. 2015; Feng et al. 2019; He et al. 2006; Qian et al. 2023; Wang et al. 2018). This might explain why the phylogenetic relationships of the *Triplophysa* strains were so chaotic. *G. przewalskii* was originally introduced as a breeding variety in the YR basin. We detected a very close genetic relationship between *G. przewalskii* and *G. eckloni* . The uplift of the Qinghai‐Tibet Plateau 150,000 years ago caused Qinghai Lake to break away from the YR system (Li et al. 2001). The *G. eckloni* from the YR isolated in Qinghai Lake gradually evolved into *G. przewalskii* , and they still share an ancient haplotype (Zhao and Duan 2007). The haplotype‐sharing phenomenon in this study was consistent with this inference. OTU sharing occurred in multiple species (Table 1). This occurred probably because of introgressive hybridization and incomplete lineage sorting (Dahruddin et al. 2017; Hubert and Hanner 2015; Sholihah et al. 2020; Sonet et al. 2019). The morphological differences between these species are relatively small, and confusion during classification and misidentification may also have led to this result. We also found artificially bred varieties of *Cyprinus carpio specularis* and *Carassius auratus gibelio* that escaped from aquatic farms during our investigation, but we could not distinguish them on the basis of sequence. This might have occurred because of extensive genetic admixture between the species (Xu et al. 2014). The above phenomenon reveals the limitations of a single barcode's recognition ability, but it provides a basis for subsequent research on increasing the number of barcodes or for using the results of this study to conduct individual identification of specific species.

## 5 Conclusions

We established the first comprehensive DNA barcode reference library for the YR ichthyofauna and provided preliminary information on the current diversity of fish species in this river. In this study, 22.9% of the fish species could not be identified through barcoding sequences, posing serious challenges to the standardization and automated molecular identification of DNA barcodes. More molecular loci are needed to understand the boundaries among these species. Since some specimens cannot be described in the literature, we believe that there is a probability of multiple new species in the YR basin; however, further studies are needed to validate this hypothesis. Combining existing data, we found that the diversity of fish species in the YR basin is severely underestimated. Therefore, this study suggested that local governments should increase investigations into fish resources in the YR basin, especially in tributaries, to better understand the current status of fish species diversity. This study provides an invasive and missing species list, standardized species identification processes, and a comprehensive database to lay the foundation for the subsequent determination, identification, and investigation of protected species.

Owing to the Chinese government's implementation of the fishing ban policy in the YR, the difficulty of large‐scale sampling will increase in the future. The advancement of sequencing technology has increased the popularity of environmental DNA (eDNA) and may mean future studies on fish no longer require traditional resource surveys. However, environmental DNA technology requires a comprehensive database, such as that used in this study, to better demonstrate its species‐identification capabilities. This study has laid a solid foundation for the future application of eDNA technology in fish in the Yellow River Basin.

## Author Contributions

**Chuanjiang Zhou:** conceptualization (equal), funding acquisition (equal), investigation (equal). **Ke Ma:** data curation (equal), formal analysis (equal), investigation (equal), methodology (equal), project administration (equal), software (equal), validation (equal), visualization (equal), writing – original draft (equal), writing – review and editing (equal). **Tai Wang:** investigation (equal), resources (equal), writing – review and editing (equal). **Yongtao Tang:** investigation (equal), resources (equal), writing – review and editing (equal). **Meng Zhang:** data curation (equal), software (equal). **Jinhui Yu:** data curation (equal). **Ruyao Liu:** investigation (equal). **Qiqi Ding:** investigation (equal). **Shangjin Qiu:** investigation (equal). **Yikai Li:** investigation (equal). **Zhigang Ma:** investigation (equal). **Guoxing Nie:** investigation (equal), resources (equal), supervision (equal), writing – review and editing (equal).

## Funding

This work was supported by the following funding bodies: the National Natural Science Foundation of China (U2004146, 31872199), the Henan Province Department of Science and Technology (182102110046, 222102110294).

## Ethics Statement

Authors declare that all experimental protocols involving animals were approved by the Animal Research and Ethics Committee of Henan Normal University and the Laboratory Animal Guidelines for the Ethical Review of Animal Welfare (GB/T 35892‐2018) and were conducted in accordance with the research standards.

## Conflicts of Interest

The authors declare no conflicts of interest.

## Supporting information


**Table S1:** List of the YR fish included in this study.

**Table S2:** List of species discovered in this study in the YR Basin.

**Table S3:** Species definition results.

**Table S4:** Results of genetic distance analysis.

**Table S5:** Comparison of freshwater fish species in the YR between this study and past studies.

**Figure S1:** ece372617‐sup‐0006‐FigueS1.pdf.

## Data Availability

All collecting and sequence data are available on the Barcode of Life Data system (BOLD) system in the project YRFIR “Yellow River Fish Identify Research” (see Table S1).
