# Supplementary material for: Assessing Current Fish Diversity in the Yellow River Basin by Integrating Large‐Scale Barcoding and Morphological Data
Source: Ecol Evol. 2025 Dec 22;15(12):e72617. doi: 10.1002/ece3.72617 (PMC12719916; doi:10.1002/ece3.72617)
Supplement: Supplementary file 6 — Figure S1: ece372617‐sup‐0006‐FigueS1.pdf. [file ECE3-15-e72617-s002.pdf]

BOLD TaxonID Tree

Title	:	Tree Result - YRFIR
Date	:	29-Oct-2023
Data Type	:	Nucleotide
Distance Model	:	Kimura 2 Parameter
Marker	:	COI-5P
Colourization	:	[blue]=Stop Codons [red]=Contamination or misidentification
Label	:	Process ID
Label	:	Taxon
Sequence Count	:	3011
Species count	:	109
Genus count	:	66
Family count	:	23
Unidentified	:	101
BIN Count	:	130

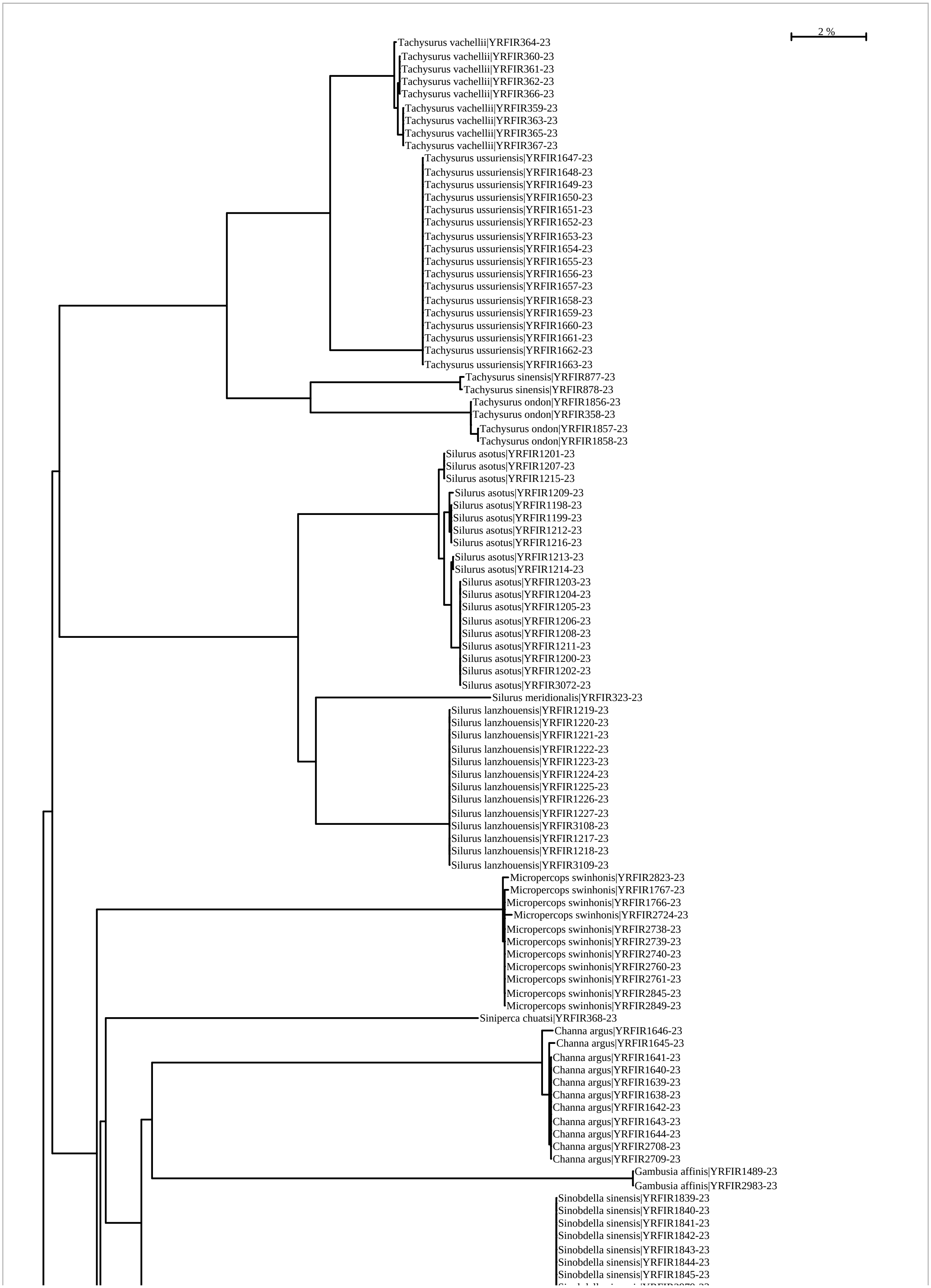
2 %
Tachysurus vachellii|YRFIR364-23
Tachysurus vachellii|YRFIR360-23
Tachysurus vachellii|YRFIR361-23
Tachysurus vachellii|YRFIR362-23
Tachysurus vachellii|YRFIR366-23
Tachysurus vachellii|YRFIR359-23
Tachysurus vachellii|YRFIR363-23
Tachysurus vachellii|YRFIR365-23
Tachysurus vachellii|YRFIR367-23
Tachysurus ussuriensis|YRFIR1647-23
Tachysurus ussuriensis|YRFIR1648-23
Tachysurus ussuriensis|YRFIR1649-23
Tachysurus ussuriensis|YRFIR1650-23
Tachysurus ussuriensis|YRFIR1651-23
Tachysurus ussuriensis|YRFIR1652-23
Tachysurus ussuriensis|YRFIR1653-23
Tachysurus ussuriensis|YRFIR1654-23
Tachysurus ussuriensis|YRFIR1655-23
Tachysurus ussuriensis|YRFIR1656-23
Tachysurus ussuriensis|YRFIR1657-23
Tachysurus ussuriensis|YRFIR1658-23
Tachysurus ussuriensis|YRFIR1659-23
Tachysurus ussuriensis|YRFIR1660-23
Tachysurus ussuriensis|YRFIR1661-23
Tachysurus ussuriensis|YRFIR1662-23
Tachysurus ussuriensis|YRFIR1663-23
Tachysurus sinensis|YRFIR877-23
Tachysurus sinensis|YRFIR878-23
Tachysurus ondon|YRFIR1856-23
Tachysurus ondon|YRFIR358-23
Tachysurus ondon|YRFIR1857-23
Tachysurus ondon|YRFIR1858-23
Silurus asotus|YRFIR1201-23
Silurus asotus|YRFIR1207-23
Silurus asotus|YRFIR1215-23
Silurus asotus|YRFIR1209-23
Silurus asotus|YRFIR1198-23
Silurus asotus|YRFIR1199-23
Silurus asotus|YRFIR1212-23
Silurus asotus|YRFIR1216-23
Silurus asotus|YRFIR1213-23
Silurus asotus|YRFIR1214-23
Silurus asotus|YRFIR1203-23
Silurus asotus|YRFIR1204-23
Silurus asotus|YRFIR1205-23
Silurus asotus|YRFIR1206-23
Silurus asotus|YRFIR1208-23
Silurus asotus|YRFIR1211-23
Silurus asotus|YRFIR1200-23
Silurus asotus|YRFIR1202-23
Silurus asotus|YRFIR3072-23
Silurus meridionalis|YRFIR323-23
Silurus lanzhouensis|YRFIR1219-23
Silurus lanzhouensis|YRFIR1220-23
Silurus lanzhouensis|YRFIR1221-23
Silurus lanzhouensis|YRFIR1222-23
Silurus lanzhouensis|YRFIR1223-23
Silurus lanzhouensis|YRFIR1224-23
Silurus lanzhouensis|YRFIR1225-23
Silurus lanzhouensis|YRFIR1226-23
Silurus lanzhouensis|YRFIR1227-23
Silurus lanzhouensis|YRFIR3108-23
Silurus lanzhouensis|YRFIR1217-23
Silurus lanzhouensis|YRFIR1218-23
Silurus lanzhouensis|YRFIR3109-23
Micropercops swinhonis|YRFIR2823-23
Micropercops swinhonis|YRFIR1767-23
Micropercops swinhonis|YRFIR1766-23
Micropercops swinhonis|YRFIR2724-23
Micropercops swinhonis|YRFIR2738-23
Micropercops swinhonis|YRFIR2739-23
Micropercops swinhonis|YRFIR2740-23
Micropercops swinhonis|YRFIR2760-23
Micropercops swinhonis|YRFIR2761-23
Micropercops swinhonis|YRFIR2845-23
Micropercops swinhonis|YRFIR2849-23
Siniperca chuatsi|YRFIR368-23
Channa argus|YRFIR1646-23
Channa argus|YRFIR1645-23
Channa argus|YRFIR1641-23
Channa argus|YRFIR1640-23
Channa argus|YRFIR1639-23
Channa argus|YRFIR1638-23
Channa argus|YRFIR1642-23
Channa argus|YRFIR1643-23
Channa argus|YRFIR1644-23
Channa argus|YRFIR2708-23
Channa argus|YRFIR2709-23
Gambusia affinis|YRFIR1489-23
Gambusia affinis|YRFIR2983-23
Sinobdella sinensis|YRFIR1839-23
Sinobdella sinensis|YRFIR1840-23
Sinobdella sinensis|YRFIR1841-23
Sinobdella sinensis|YRFIR1842-23
Sinobdella sinensis|YRFIR1843-23
Sinobdella sinensis|YRFIR1844-23
Sinobdella sinensis|YRFIR1845-23

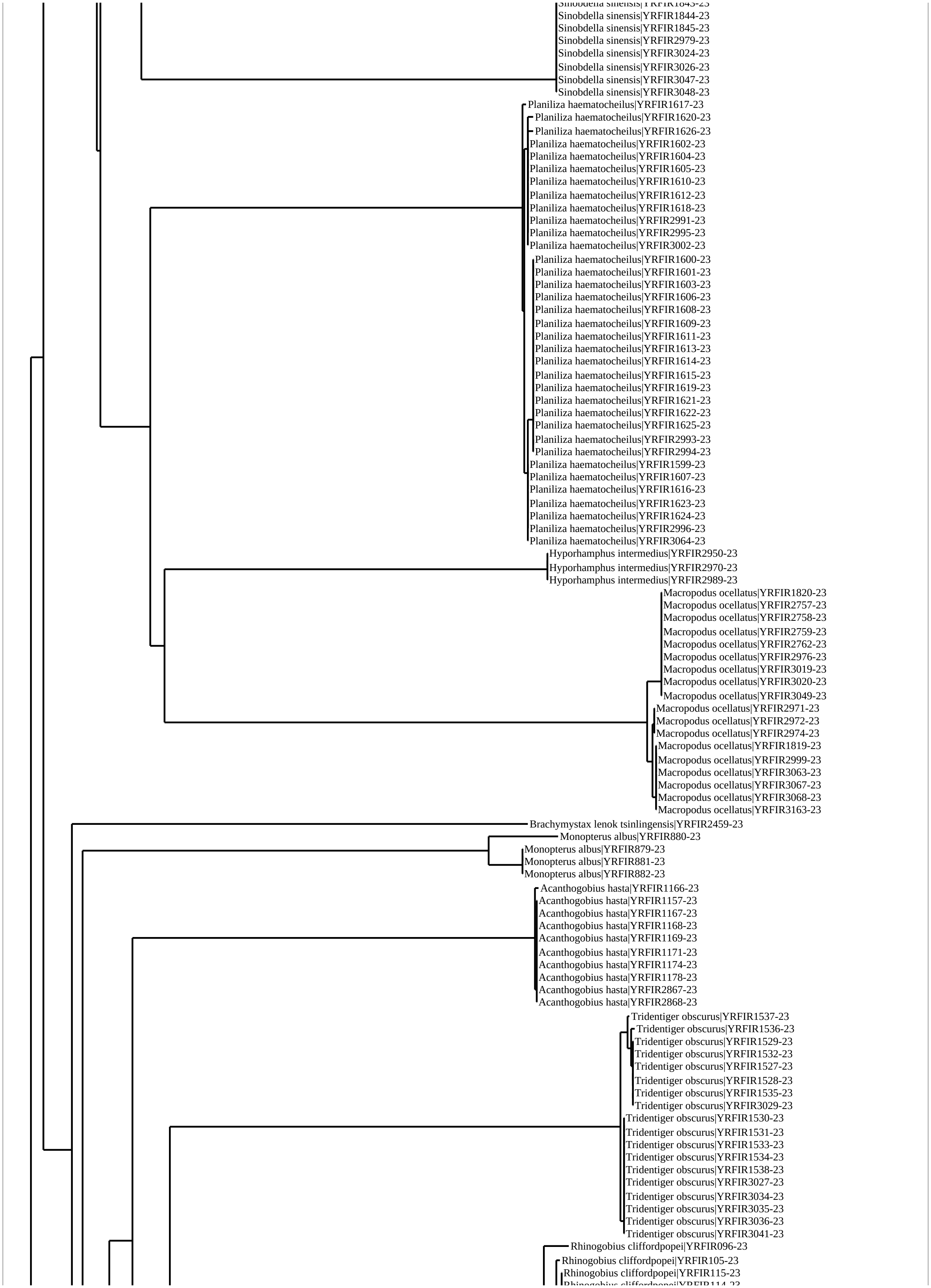
Sinobdella sinensis|YRFIR1844-23
Sinobdella sinensis|YRFIR1845-23
Sinobdella sinensis|YRFIR2979-23
Sinobdella sinensis|YRFIR3024-23
Sinobdella sinensis|YRFIR3026-23
Sinobdella sinensis|YRFIR3047-23
Sinobdella sinensis|YRFIR3048-23
Planiliza haematocheilus|YRFIR1617-23
Planiliza haematocheilus|YRFIR1620-23
Planiliza haematocheilus|YRFIR1626-23
Planiliza haematocheilus|YRFIR1602-23
Planiliza haematocheilus|YRFIR1604-23
Planiliza haematocheilus|YRFIR1605-23
Planiliza haematocheilus|YRFIR1610-23
Planiliza haematocheilus|YRFIR1612-23
Planiliza haematocheilus|YRFIR1618-23
Planiliza haematocheilus|YRFIR2991-23
Planiliza haematocheilus|YRFIR2995-23
Planiliza haematocheilus|YRFIR3002-23
Planiliza haematocheilus|YRFIR1600-23
Planiliza haematocheilus|YRFIR1601-23
Planiliza haematocheilus|YRFIR1603-23
Planiliza haematocheilus|YRFIR1606-23
Planiliza haematocheilus|YRFIR1608-23
Planiliza haematocheilus|YRFIR1609-23
Planiliza haematocheilus|YRFIR1611-23
Planiliza haematocheilus|YRFIR1613-23
Planiliza haematocheilus|YRFIR1614-23
Planiliza haematocheilus|YRFIR1615-23
Planiliza haematocheilus|YRFIR1619-23
Planiliza haematocheilus|YRFIR1621-23
Planiliza haematocheilus|YRFIR1622-23
Planiliza haematocheilus|YRFIR1625-23
Planiliza haematocheilus|YRFIR2993-23
Planiliza haematocheilus|YRFIR2994-23
Planiliza haematocheilus|YRFIR1599-23
Planiliza haematocheilus|YRFIR1607-23
Planiliza haematocheilus|YRFIR1616-23
Planiliza haematocheilus|YRFIR1623-23
Planiliza haematocheilus|YRFIR1624-23
Planiliza haematocheilus|YRFIR2996-23
Planiliza haematocheilus|YRFIR3064-23
Hyporhamphus intermedius|YRFIR2950-23
Hyporhamphus intermedius|YRFIR2970-23
Hyporhamphus intermedius|YRFIR2989-23
Macropodus ocellatus|YRFIR1820-23
Macropodus ocellatus|YRFIR2757-23
Macropodus ocellatus|YRFIR2758-23
Macropodus ocellatus|YRFIR2759-23
Macropodus ocellatus|YRFIR2762-23
Macropodus ocellatus|YRFIR2976-23
Macropodus ocellatus|YRFIR3019-23
Macropodus ocellatus|YRFIR3020-23
Macropodus ocellatus|YRFIR3049-23
Macropodus ocellatus|YRFIR2971-23
Macropodus ocellatus|YRFIR2972-23
Macropodus ocellatus|YRFIR2974-23
Macropodus ocellatus|YRFIR1819-23
Macropodus ocellatus|YRFIR2999-23
Macropodus ocellatus|YRFIR3063-23
Macropodus ocellatus|YRFIR3067-23
Macropodus ocellatus|YRFIR3068-23
Macropodus ocellatus|YRFIR3163-23
Brachymystax lenok tsinlingensis|YRFIR2459-23
Monopterus albus|YRFIR880-23
Monopterus albus|YRFIR879-23
Monopterus albus|YRFIR881-23
Monopterus albus|YRFIR882-23
Acanthogobius hasta|YRFIR1166-23
Acanthogobius hasta|YRFIR1157-23
Acanthogobius hasta|YRFIR1167-23
Acanthogobius hasta|YRFIR1168-23
Acanthogobius hasta|YRFIR1169-23
Acanthogobius hasta|YRFIR1171-23
Acanthogobius hasta|YRFIR1174-23
Acanthogobius hasta|YRFIR1178-23
Acanthogobius hasta|YRFIR2867-23
Acanthogobius hasta|YRFIR2868-23
Tridentiger obscurus|YRFIR1537-23
Tridentiger obscurus|YRFIR1536-23
Tridentiger obscurus|YRFIR1529-23
Tridentiger obscurus|YRFIR1532-23
Tridentiger obscurus|YRFIR1527-23
Tridentiger obscurus|YRFIR1528-23
Tridentiger obscurus|YRFIR1535-23
Tridentiger obscurus|YRFIR3029-23
Tridentiger obscurus|YRFIR1530-23
Tridentiger obscurus|YRFIR1531-23
Tridentiger obscurus|YRFIR1533-23
Tridentiger obscurus|YRFIR1534-23
Tridentiger obscurus|YRFIR1538-23
Tridentiger obscurus|YRFIR3027-23
Tridentiger obscurus|YRFIR3034-23
Tridentiger obscurus|YRFIR3035-23
Tridentiger obscurus|YRFIR3036-23
Tridentiger obscurus|YRFIR3041-23
Rhinogobius cliffordpopei|YRFIR096-23
Rhinogobius cliffordpopei|YRFIR105-23
Rhinogobius cliffordpopei|YRFIR115-23

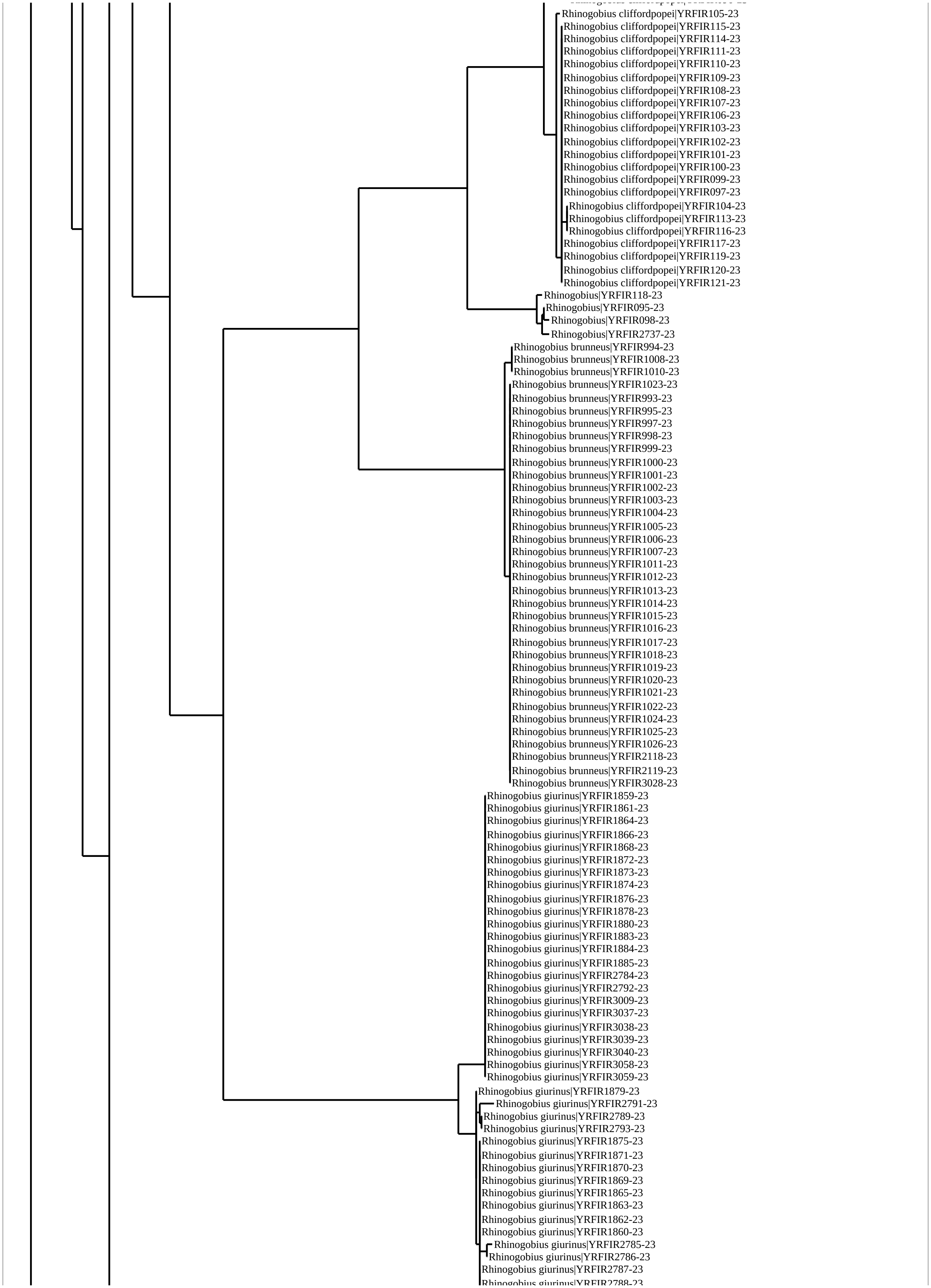
Rhinogobius cliffordpopei|YRFIR105-23
Rhinogobius cliffordpopei|YRFIR115-23
Rhinogobius cliffordpopei|YRFIR114-23
Rhinogobius cliffordpopei|YRFIR111-23
Rhinogobius cliffordpopei|YRFIR110-23
Rhinogobius cliffordpopei|YRFIR109-23
Rhinogobius cliffordpopei|YRFIR108-23
Rhinogobius cliffordpopei|YRFIR107-23
Rhinogobius cliffordpopei|YRFIR106-23
Rhinogobius cliffordpopei|YRFIR103-23
Rhinogobius cliffordpopei|YRFIR102-23
Rhinogobius cliffordpopei|YRFIR101-23
Rhinogobius cliffordpopei|YRFIR100-23
Rhinogobius cliffordpopei|YRFIR099-23
Rhinogobius cliffordpopei|YRFIR097-23
Rhinogobius cliffordpopei|YRFIR104-23
Rhinogobius cliffordpopei|YRFIR113-23
Rhinogobius cliffordpopei|YRFIR116-23
Rhinogobius cliffordpopei|YRFIR117-23
Rhinogobius cliffordpopei|YRFIR119-23
Rhinogobius cliffordpopei|YRFIR120-23
Rhinogobius cliffordpopei|YRFIR121-23
Rhinogobius|YRFIR118-23
Rhinogobius|YRFIR095-23
Rhinogobius|YRFIR098-23
Rhinogobius|YRFIR2737-23
Rhinogobius brunneus|YRFIR994-23
Rhinogobius brunneus|YRFIR1008-23
Rhinogobius brunneus|YRFIR1010-23
Rhinogobius brunneus|YRFIR1023-23
Rhinogobius brunneus|YRFIR993-23
Rhinogobius brunneus|YRFIR995-23
Rhinogobius brunneus|YRFIR997-23
Rhinogobius brunneus|YRFIR998-23
Rhinogobius brunneus|YRFIR999-23
Rhinogobius brunneus|YRFIR1000-23
Rhinogobius brunneus|YRFIR1001-23
Rhinogobius brunneus|YRFIR1002-23
Rhinogobius brunneus|YRFIR1003-23
Rhinogobius brunneus|YRFIR1004-23
Rhinogobius brunneus|YRFIR1005-23
Rhinogobius brunneus|YRFIR1006-23
Rhinogobius brunneus|YRFIR1007-23
Rhinogobius brunneus|YRFIR1011-23
Rhinogobius brunneus|YRFIR1012-23
Rhinogobius brunneus|YRFIR1013-23
Rhinogobius brunneus|YRFIR1014-23
Rhinogobius brunneus|YRFIR1015-23
Rhinogobius brunneus|YRFIR1016-23
Rhinogobius brunneus|YRFIR1017-23
Rhinogobius brunneus|YRFIR1018-23
Rhinogobius brunneus|YRFIR1019-23
Rhinogobius brunneus|YRFIR1020-23
Rhinogobius brunneus|YRFIR1021-23
Rhinogobius brunneus|YRFIR1022-23
Rhinogobius brunneus|YRFIR1024-23
Rhinogobius brunneus|YRFIR1025-23
Rhinogobius brunneus|YRFIR1026-23
Rhinogobius brunneus|YRFIR2118-23
Rhinogobius brunneus|YRFIR2119-23
Rhinogobius brunneus|YRFIR3028-23
Rhinogobius giurinus|YRFIR1859-23
Rhinogobius giurinus|YRFIR1861-23
Rhinogobius giurinus|YRFIR1864-23
Rhinogobius giurinus|YRFIR1866-23
Rhinogobius giurinus|YRFIR1868-23
Rhinogobius giurinus|YRFIR1872-23
Rhinogobius giurinus|YRFIR1873-23
Rhinogobius giurinus|YRFIR1874-23
Rhinogobius giurinus|YRFIR1876-23
Rhinogobius giurinus|YRFIR1878-23
Rhinogobius giurinus|YRFIR1880-23
Rhinogobius giurinus|YRFIR1883-23
Rhinogobius giurinus|YRFIR1884-23
Rhinogobius giurinus|YRFIR1885-23
Rhinogobius giurinus|YRFIR2784-23
Rhinogobius giurinus|YRFIR2792-23
Rhinogobius giurinus|YRFIR3009-23
Rhinogobius giurinus|YRFIR3037-23
Rhinogobius giurinus|YRFIR3038-23
Rhinogobius giurinus|YRFIR3039-23
Rhinogobius giurinus|YRFIR3040-23
Rhinogobius giurinus|YRFIR3058-23
Rhinogobius giurinus|YRFIR3059-23
Rhinogobius giurinus|YRFIR1879-23
Rhinogobius giurinus|YRFIR2791-23
Rhinogobius giurinus|YRFIR2789-23
Rhinogobius giurinus|YRFIR2793-23
Rhinogobius giurinus|YRFIR1875-23
Rhinogobius giurinus|YRFIR1871-23
Rhinogobius giurinus|YRFIR1870-23
Rhinogobius giurinus|YRFIR1869-23
Rhinogobius giurinus|YRFIR1865-23
Rhinogobius giurinus|YRFIR1863-23
Rhinogobius giurinus|YRFIR1862-23
Rhinogobius giurinus|YRFIR1860-23
Rhinogobius giurinus|YRFIR2785-23
Rhinogobius giurinus|YRFIR2786-23
Rhinogobius giurinus|YRFIR2787-23
Rhinogobius giurinus|YRFIR2788-23

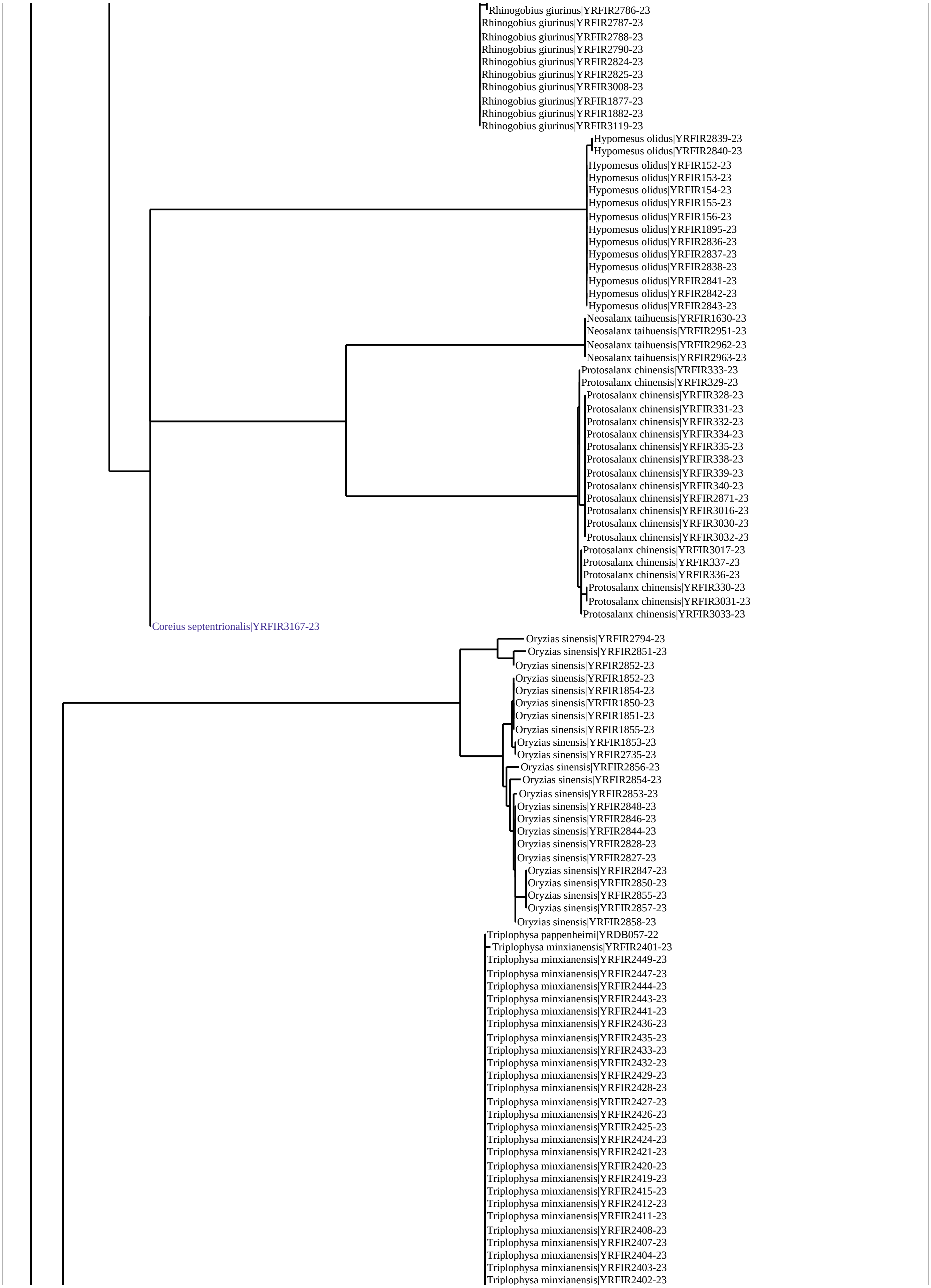

Rhinogobius giurinus|YRFIR2786-23
Rhinogobius giurinus|YRFIR2787-23
Rhinogobius giurinus|YRFIR2788-23
Rhinogobius giurinus|YRFIR2790-23
Rhinogobius giurinus|YRFIR2824-23
Rhinogobius giurinus|YRFIR2825-23
Rhinogobius giurinus|YRFIR3008-23
Rhinogobius giurinus|YRFIR1877-23
Rhinogobius giurinus|YRFIR1882-23
Rhinogobius giurinus|YRFIR3119-23
Hypomesus olidus|YRFIR2839-23
Hypomesus olidus|YRFIR2840-23
Hypomesus olidus|YRFIR152-23
Hypomesus olidus|YRFIR153-23
Hypomesus olidus|YRFIR154-23
Hypomesus olidus|YRFIR155-23
Hypomesus olidus|YRFIR156-23
Hypomesus olidus|YRFIR1895-23
Hypomesus olidus|YRFIR2836-23
Hypomesus olidus|YRFIR2837-23
Hypomesus olidus|YRFIR2838-23
Hypomesus olidus|YRFIR2841-23
Hypomesus olidus|YRFIR2842-23
Hypomesus olidus|YRFIR2843-23
Neosalanx taihuensis|YRFIR1630-23
Neosalanx taihuensis|YRFIR2951-23
Neosalanx taihuensis|YRFIR2962-23
Neosalanx taihuensis|YRFIR2963-23
Protosalanx chinensis|YRFIR333-23
Protosalanx chinensis|YRFIR329-23
Protosalanx chinensis|YRFIR328-23
Protosalanx chinensis|YRFIR331-23
Protosalanx chinensis|YRFIR332-23
Protosalanx chinensis|YRFIR334-23
Protosalanx chinensis|YRFIR335-23
Protosalanx chinensis|YRFIR338-23
Protosalanx chinensis|YRFIR339-23
Protosalanx chinensis|YRFIR340-23
Protosalanx chinensis|YRFIR2871-23
Protosalanx chinensis|YRFIR3016-23
Protosalanx chinensis|YRFIR3030-23
Protosalanx chinensis|YRFIR3032-23
Protosalanx chinensis|YRFIR3017-23
Protosalanx chinensis|YRFIR337-23
Protosalanx chinensis|YRFIR336-23
Protosalanx chinensis|YRFIR330-23
Protosalanx chinensis|YRFIR3031-23
Protosalanx chinensis|YRFIR3033-23
Coreius septentrionalis|YRFIR3167-23
Oryzias sinensis|YRFIR2794-23
Oryzias sinensis|YRFIR2851-23
Oryzias sinensis|YRFIR2852-23
Oryzias sinensis|YRFIR1852-23
Oryzias sinensis|YRFIR1854-23
Oryzias sinensis|YRFIR1850-23
Oryzias sinensis|YRFIR1851-23
Oryzias sinensis|YRFIR1855-23
Oryzias sinensis|YRFIR1853-23
Oryzias sinensis|YRFIR2735-23
Oryzias sinensis|YRFIR2856-23
Oryzias sinensis|YRFIR2854-23
Oryzias sinensis|YRFIR2853-23
Oryzias sinensis|YRFIR2848-23
Oryzias sinensis|YRFIR2846-23
Oryzias sinensis|YRFIR2844-23
Oryzias sinensis|YRFIR2828-23
Oryzias sinensis|YRFIR2827-23
Oryzias sinensis|YRFIR2847-23
Oryzias sinensis|YRFIR2850-23
Oryzias sinensis|YRFIR2855-23
Oryzias sinensis|YRFIR2857-23
Oryzias sinensis|YRFIR2858-23
Triplophysa pappenheimi|YRDB057-22
Triplophysa minxianensis|YRFIR2401-23
Triplophysa minxianensis|YRFIR2449-23
Triplophysa minxianensis|YRFIR2447-23
Triplophysa minxianensis|YRFIR2444-23
Triplophysa minxianensis|YRFIR2443-23
Triplophysa minxianensis|YRFIR2441-23
Triplophysa minxianensis|YRFIR2436-23
Triplophysa minxianensis|YRFIR2435-23
Triplophysa minxianensis|YRFIR2433-23
Triplophysa minxianensis|YRFIR2432-23
Triplophysa minxianensis|YRFIR2429-23
Triplophysa minxianensis|YRFIR2428-23
Triplophysa minxianensis|YRFIR2427-23
Triplophysa minxianensis|YRFIR2426-23
Triplophysa minxianensis|YRFIR2425-23
Triplophysa minxianensis|YRFIR2424-23
Triplophysa minxianensis|YRFIR2421-23
Triplophysa minxianensis|YRFIR2420-23
Triplophysa minxianensis|YRFIR2419-23
Triplophysa minxianensis|YRFIR2415-23
Triplophysa minxianensis|YRFIR2412-23
Triplophysa minxianensis|YRFIR2411-23
Triplophysa minxianensis|YRFIR2408-23
Triplophysa minxianensis|YRFIR2407-23
Triplophysa minxianensis|YRFIR2404-23
Triplophysa minxianensis|YRFIR2403-23
Triplophysa minxianensis|YRFIR2402-23

Triplophysa minxianensis|YRFIR2404-23
Triplophysa minxianensis|YRFIR2403-23
Triplophysa minxianensis|YRFIR2402-23
Triplophysa minxianensis|YRFIR2400-23
Triplophysa minxianensis|YRFIR2399-23
Triplophysa minxianensis|YRFIR2398-23
Triplophysa minxianensis|YRFIR2397-23
Triplophysa minxianensis|YRFIR2396-23
Triplophysa minxianensis|YRFIR2395-23
Triplophysa minxianensis|YRFIR2394-23
Triplophysa minxianensis|YRFIR2393-23
Triplophysa minxianensis|YRFIR2392-23
Triplophysa minxianensis|YRFIR2391-23
Triplophysa minxianensis|YRFIR2390-23
Triplophysa minxianensis|YRFIR2388-23
Triplophysa minxianensis|YRFIR2385-23
Triplophysa minxianensis|YRFIR2384-23
Triplophysa minxianensis|YRFIR2381-23
Triplophysa minxianensis|YRFIR2379-23
Triplophysa minxianensis|YRFIR2378-23
Triplophysa minxianensis|YRFIR2377-23
Triplophysa minxianensis|YRFIR2376-23
Triplophysa minxianensis|YRFIR2375-23
Triplophysa minxianensis|YRFIR2373-23
Triplophysa minxianensis|YRFIR2371-23
Triplophysa minxianensis|YRFIR2370-23
Triplophysa minxianensis|YRFIR2369-23
Triplophysa minxianensis|YRFIR2368-23
Triplophysa minxianensis|YRFIR2367-23
Triplophysa minxianensis|YRFIR2366-23
Triplophysa minxianensis|YRFIR2363-23
Triplophysa minxianensis|YRFIR2361-23
Triplophysa minxianensis|YRFIR2355-23
Triplophysa minxianensis|YRFIR2348-23
Triplophysa minxianensis|YRFIR2347-23
Triplophysa minxianensis|YRFIR2345-23
Triplophysa minxianensis|YRFIR2343-23
Triplophysa minxianensis|YRFIR2342-23
Triplophysa minxianensis|YRFIR2341-23
Triplophysa minxianensis|YRFIR2337-23
Triplophysa minxianensis|YRFIR2336-23
Triplophysa minxianensis|YRFIR2334-23
Triplophysa minxianensis|YRFIR2333-23
Triplophysa minxianensis|YRFIR2330-23
Triplophysa minxianensis|YRFIR2329-23
Triplophysa minxianensis|YRFIR2327-23
Triplophysa minxianensis|YRFIR2326-23
Triplophysa minxianensis|YRFIR2323-23
Triplophysa minxianensis|YRFIR2322-23
Triplophysa minxianensis|YRFIR2320-23
Triplophysa minxianensis|YRFIR2316-23
Triplophysa minxianensis|YRFIR2315-23
Triplophysa minxianensis|YRFIR2313-23
Triplophysa minxianensis|YRFIR2311-23
Triplophysa minxianensis|YRFIR2310-23
Triplophysa minxianensis|YRFIR2308-23
Triplophysa minxianensis|YRFIR2307-23
Triplophysa minxianensis|YRFIR2306-23
Triplophysa minxianensis|YRFIR2305-23
Triplophysa minxianensis|YRFIR2302-23
Triplophysa minxianensis|YRFIR2299-23
Triplophysa minxianensis|YRFIR2298-23
Triplophysa minxianensis|YRFIR2294-23
Triplophysa minxianensis|YRFIR2292-23
Triplophysa minxianensis|YRFIR2291-23
Triplophysa minxianensis|YRFIR2290-23
Triplophysa minxianensis|YRFIR2289-23
Triplophysa minxianensis|YRFIR2288-23
Triplophysa minxianensis|YRFIR2287-23
Triplophysa minxianensis|YRFIR2281-23
Triplophysa minxianensis|YRFIR2280-23
Triplophysa minxianensis|YRFIR2279-23
Triplophysa minxianensis|YRFIR2277-23
Triplophysa minxianensis|YRFIR2275-23
Triplophysa minxianensis|YRFIR2274-23
Triplophysa minxianensis|YRFIR2271-23
Triplophysa minxianensis|YRFIR2270-23
Triplophysa minxianensis|YRFIR2267-23
Triplophysa minxianensis|YRFIR2266-23
Triplophysa minxianensis|YRFIR2265-23
Triplophysa minxianensis|YRFIR2264-23
Triplophysa minxianensis|YRFIR2262-23
Triplophysa minxianensis|YRFIR2257-23
Triplophysa minxianensis|YRFIR2255-23
Triplophysa minxianensis|YRFIR2253-23
Triplophysa minxianensis|YRFIR2251-23
Triplophysa minxianensis|YRFIR2250-23
Triplophysa minxianensis|YRFIR2249-23
Triplophysa minxianensis|YRFIR2245-23
Triplophysa minxianensis|YRFIR2244-23
Triplophysa minxianensis|YRFIR2240-23
Triplophysa minxianensis|YRFIR2239-23
Triplophysa minxianensis|YRFIR2237-23
Triplophysa minxianensis|YRFIR2236-23
Triplophysa minxianensis|YRFIR2235-23
Triplophysa minxianensis|YRFIR2234-23
Triplophysa minxianensis|YRFIR2233-23
Triplophysa minxianensis|YRFIR2232-23
Triplophysa minxianensis|YRFIR2231-23
Triplophysa minxianensis|YRFIR2229-23

Triplophysa minxianensis|YRFIR2232-23
Triplophysa minxianensis|YRFIR2231-23
Triplophysa minxianensis|YRFIR2229-23
Triplophysa minxianensis|YRFIR2228-23
Triplophysa minxianensis|YRFIR2227-23
Triplophysa minxianensis|YRFIR2225-23
Triplophysa minxianensis|YRFIR2223-23
Triplophysa minxianensis|YRFIR2219-23
Triplophysa minxianensis|YRFIR2214-23
Triplophysa minxianensis|YRFIR2213-23
Triplophysa minxianensis|YRFIR2211-23
Triplophysa minxianensis|YRFIR2209-23
Triplophysa minxianensis|YRFIR2208-23
Triplophysa minxianensis|YRFIR2207-23
Triplophysa minxianensis|YRFIR2206-23
Triplophysa minxianensis|YRFIR2205-23
Triplophysa minxianensis|YRFIR2204-23
Triplophysa minxianensis|YRFIR2203-23
Triplophysa pappenheimi|YRFIR870-23
Triplophysa minxianensis|YRFIR2301-23
Triplophysa minxianensis|YRFIR2241-23
Triplophysa minxianensis|YRFIR2246-23
Triplophysa minxianensis|YRFIR2276-23
Triplophysa minxianensis|YRFIR2364-23
Triplophysa minxianensis|YRFIR2430-23
Triplophysa minxianensis|YRFIR2431-23
Triplophysa minxianensis|YRFIR2248-23
Triplophysa minxianensis|YRFIR2260-23
Triplophysa minxianensis|YRFIR2221-23
Triplophysa minxianensis|YRFIR2309-23
Triplophysa minxianensis|YRFIR2242-23
Triplophysa minxianensis|YRFIR2254-23
Triplophysa minxianensis|YRFIR2258-23
Triplophysa minxianensis|YRFIR2259-23
Triplophysa minxianensis|YRFIR2261-23
Triplophysa minxianensis|YRFIR2268-23
Triplophysa minxianensis|YRFIR2269-23
Triplophysa minxianensis|YRFIR2272-23
Triplophysa minxianensis|YRFIR2286-23
Triplophysa minxianensis|YRFIR2339-23
Triplophysa minxianensis|YRFIR2328-23
Triplophysa minxianensis|YRFIR2410-23
Triplophysa minxianensis|YRFIR2238-23
Triplophysa minxianensis|YRFIR2317-23
Triplophysa minxianensis|YRFIR2296-23
Triplophysa minxianensis|YRFIR2314-23
Triplophysa minxianensis|YRFIR2423-23
Triplophysa minxianensis|YRFIR2422-23
Triplophysa minxianensis|YRFIR2416-23
Triplophysa minxianensis|YRFIR2414-23
Triplophysa minxianensis|YRFIR2380-23
Triplophysa minxianensis|YRFIR2344-23
Triplophysa minxianensis|YRFIR2325-23
Triplophysa minxianensis|YRFIR2324-23
Triplophysa minxianensis|YRFIR2304-23
Triplophysa minxianensis|YRFIR2300-23
Triplophysa minxianensis|YRFIR2293-23
Triplophysa minxianensis|YRFIR2284-23
Triplophysa minxianensis|YRFIR2283-23
Triplophysa minxianensis|YRFIR2273-23
Triplophysa minxianensis|YRFIR2263-23
Triplophysa minxianensis|YRFIR2220-23
Triplophysa minxianensis|YRFIR2212-23
Triplophysa minxianensis|YRFIR867-23
Triplophysa minxianensis|YRFIR2448-23
Triplophysa pappenheimi|YRFIR869-23
Triplophysa minxianensis|YRFIR2374-23
Triplophysa minxianensis|YRFIR2405-23
Triplophysa minxianensis|YRFIR2278-23
Triplophysa minxianensis|YRFIR2224-23
Triplophysa minxianensis|YRFIR2285-23
Triplophysa pappenheimi|YRFIR809-23
Triplophysa pappenheimi|YRFIR817-23
Triplophysa pappenheimi|YRFIR805-23
Triplophysa pappenheimi|YRFIR807-23
Triplophysa pappenheimi|YRFIR803-23
Triplophysa pappenheimi|YRFIR844-23
Triplophysa pappenheimi|YRFIR849-23
Triplophysa pappenheimi|YRFIR866-23
Triplophysa pappenheimi|YRFIR868-23
Triplophysa minxianensis|YRFIR2201-23
Triplophysa minxianensis|YRFIR2216-23
Triplophysa minxianensis|YRFIR2217-23
Triplophysa minxianensis|YRFIR2218-23
Triplophysa minxianensis|YRFIR2295-23
Triplophysa minxianensis|YRFIR2312-23
Triplophysa minxianensis|YRFIR2321-23
Triplophysa minxianensis|YRFIR2417-23
Triplophysa minxianensis|YRFIR2340-23
Triplophysa minxianensis|YRFIR2319-23
Triplophysa pappenheimi|YRFIR2170-23
Triplophysa pappenheimi|YRDB055-22
Triplophysa pappenheimi|YRFIR2167-23
Triplophysa pappenheimi|YRFIR2168-23
Triplophysa pappenheimi|YRFIR813-23
Triplophysa pappenheimi|YRFIR2166-23
Triplophysa pappenheimi|YRDB058-22
Triplophysa pappenheimi|YRFIR812-23
Triplophysa pappenheimi|YRFIR811-23
Triplophysa pappenheimi|YRFIR781-23
[illegible]

Triplophysa pappenheimi|YRFIR812-23
Triplophysa pappenheimi|YRFIR811-23
Triplophysa pappenheimi|YRFIR781-23
Triplophysa pappenheimi|YRFIR757-23
Triplophysa siluroides|YRFIR2483-23
Triplophysa siluroides|YRFIR2499-23
Triplophysa siluroides|YRFIR2497-23
Triplophysa siluroides|YRFIR2496-23
Triplophysa siluroides|YRFIR2495-23
Triplophysa siluroides|YRFIR2494-23
Triplophysa siluroides|YRFIR2493-23
Triplophysa siluroides|YRFIR2492-23
Triplophysa siluroides|YRFIR2491-23
Triplophysa siluroides|YRFIR2490-23
Triplophysa siluroides|YRFIR2489-23
Triplophysa siluroides|YRFIR2488-23
Triplophysa siluroides|YRFIR2487-23
Triplophysa siluroides|YRFIR2486-23
Triplophysa siluroides|YRFIR2485-23
Triplophysa siluroides|YRFIR2484-23
Triplophysa siluroides|YRFIR2479-23
Triplophysa siluroides|YRFIR876-23
Triplophysa siluroides|YRFIR875-23
Triplophysa siluroides|YRFIR874-23
Triplophysa siluroides|YRFIR873-23
Triplophysa siluroides|YRFIR864-23
Triplophysa siluroides|YRFIR847-23
Triplophysa siluroides|YRFIR837-23
Triplophysa siluroides|YRFIR833-23
Triplophysa siluroides|YRFIR827-23
Triplophysa siluroides|YRFIR810-23
Triplophysa siluroides|YRFIR808-23
Triplophysa siluroides|YRFIR806-23
Triplophysa siluroides|YRFIR804-23
Triplophysa siluroides|YRFIR815-23
Triplophysa siluroides|YRFIR819-23
Triplophysa siluroides|YRFIR820-23
Triplophysa siluroides|YRFIR822-23
Triplophysa siluroides|YRFIR823-23
Triplophysa siluroides|YRFIR825-23
Triplophysa siluroides|YRFIR826-23
Triplophysa siluroides|YRFIR830-23
Triplophysa siluroides|YRFIR832-23
Triplophysa siluroides|YRFIR835-23
Triplophysa siluroides|YRFIR845-23
Triplophysa siluroides|YRFIR848-23
Triplophysa siluroides|YRFIR850-23
Triplophysa pappenheimi|YRFIR854-23
Triplophysa pappenheimi|YRFIR856-23
Triplophysa pappenheimi|YRFIR859-23
Triplophysa pappenheimi|YRFIR863-23
Triplophysa pappenheimi|YRFIR865-23
Triplophysa pappenheimi|YRFIR871-23
Triplophysa siluroides|YRFIR2480-23
Triplophysa siluroides|YRFIR2481-23
Triplophysa siluroides|YRFIR2498-23
Triplophysa pappenheimi|YRDB056-22
Triplophysa siluroides|YRFIR814-23
Triplophysa pappenheimi|YRFIR861-23
Triplophysa pappenheimi|YRFIR862-23
Triplophysa pappenheimi|YRFIR857-23
Triplophysa pappenheimi|YRFIR858-23
Triplophysa siluroides|YRFIR853-23
Triplophysa siluroides|YRFIR855-23
Triplophysa siluroides|YRFIR851-23
Triplophysa siluroides|YRFIR852-23
Triplophysa siluroides|YRFIR843-23
Triplophysa siluroides|YRFIR846-23
Triplophysa siluroides|YRFIR839-23
Triplophysa siluroides|YRFIR841-23
Triplophysa siluroides|YRFIR836-23
Triplophysa siluroides|YRFIR838-23
Triplophysa siluroides|YRFIR831-23
Triplophysa siluroides|YRFIR834-23
Triplophysa pappenheimi|YRFIR2169-23
Triplophysa siluroides|YRFIR829-23
Triplophysa siluroides|YRFIR828-23
Triplophysa siluroides|YRFIR824-23
Triplophysa siluroides|YRFIR821-23
Triplophysa siluroides|YRFIR818-23
Triplophysa siluroides|YRFIR816-23
Triplophysa pappenheimi|YRFIR2964-23
Triplophysa siluroides|YRFIR2482-23
Triplophysa robusta|YRFIR1971-23
Triplophysa robusta|YRFIR1972-23
Triplophysa robusta|YRFIR1973-23
Triplophysa robusta|YRFIR1981-23
Triplophysa robusta|YRFIR1982-23
Triplophysa robusta|YRFIR1985-23
Triplophysa robusta|YRFIR1988-23
Triplophysa robusta|YRFIR1989-23
Triplophysa robusta|YRFIR1991-23
Triplophysa robusta|YRFIR1992-23
Triplophysa robusta|YRFIR1996-23
Triplophysa robusta|YRFIR1997-23
Triplophysa robusta|YRFIR2000-23
Triplophysa robusta|YRFIR2004-23
Triplophysa robusta|YRFIR2005-23
Triplophysa robusta|YRFIR2010-23
Triplophysa robusta|YRFIR2014-23
[illegible]

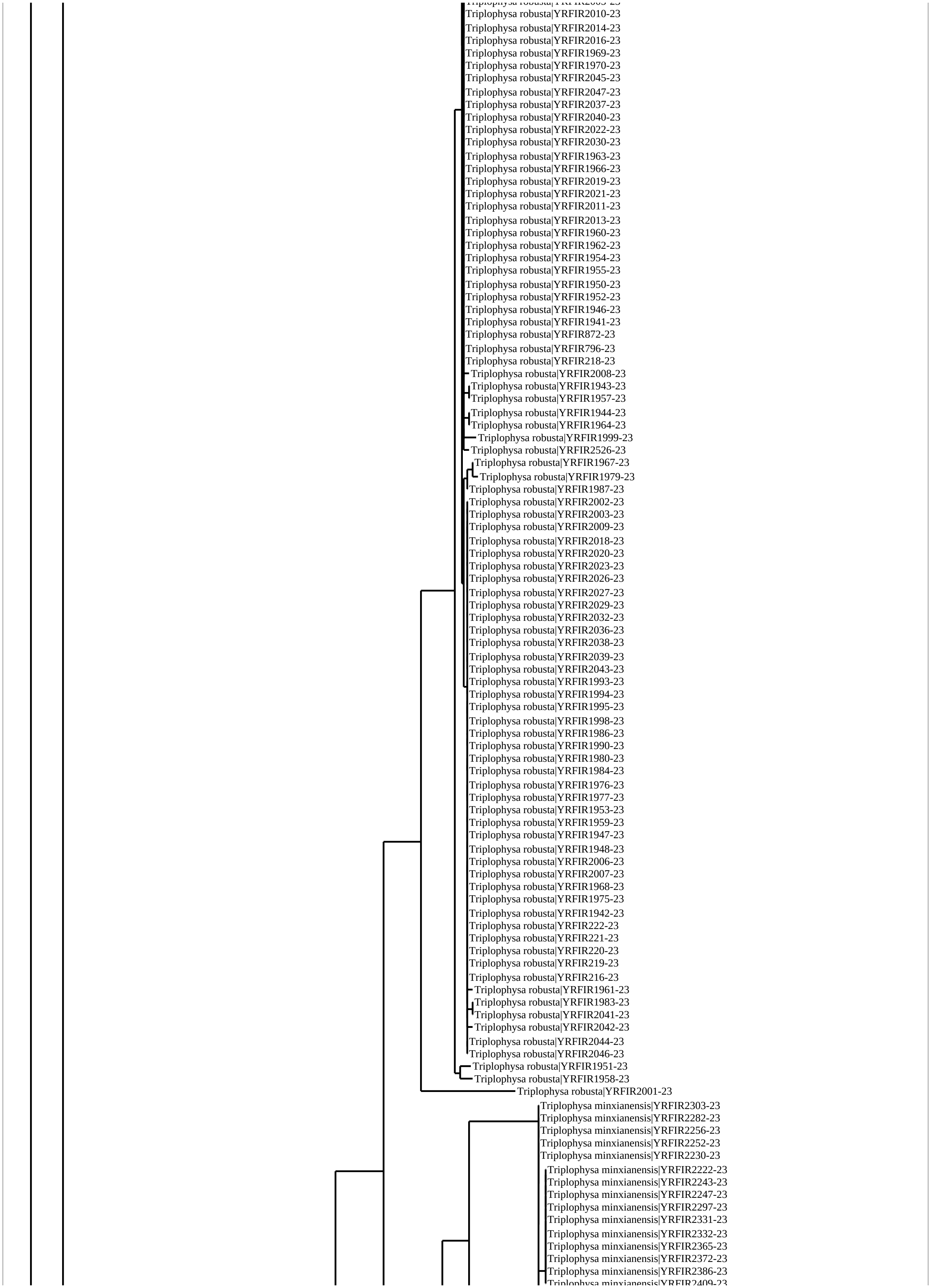

Triplophysa robusta|YRFIR2010-23
Triplophysa robusta|YRFIR2014-23
Triplophysa robusta|YRFIR2016-23
Triplophysa robusta|YRFIR1969-23
Triplophysa robusta|YRFIR1970-23
Triplophysa robusta|YRFIR2045-23
Triplophysa robusta|YRFIR2047-23
Triplophysa robusta|YRFIR2037-23
Triplophysa robusta|YRFIR2040-23
Triplophysa robusta|YRFIR2022-23
Triplophysa robusta|YRFIR2030-23
Triplophysa robusta|YRFIR1963-23
Triplophysa robusta|YRFIR1966-23
Triplophysa robusta|YRFIR2019-23
Triplophysa robusta|YRFIR2021-23
Triplophysa robusta|YRFIR2011-23
Triplophysa robusta|YRFIR2013-23
Triplophysa robusta|YRFIR1960-23
Triplophysa robusta|YRFIR1962-23
Triplophysa robusta|YRFIR1954-23
Triplophysa robusta|YRFIR1955-23
Triplophysa robusta|YRFIR1950-23
Triplophysa robusta|YRFIR1952-23
Triplophysa robusta|YRFIR1946-23
Triplophysa robusta|YRFIR1941-23
Triplophysa robusta|YRFIR872-23
Triplophysa robusta|YRFIR796-23
Triplophysa robusta|YRFIR218-23
Triplophysa robusta|YRFIR2008-23
Triplophysa robusta|YRFIR1943-23
Triplophysa robusta|YRFIR1957-23
Triplophysa robusta|YRFIR1944-23
Triplophysa robusta|YRFIR1964-23
Triplophysa robusta|YRFIR1999-23
Triplophysa robusta|YRFIR2526-23
Triplophysa robusta|YRFIR1967-23
Triplophysa robusta|YRFIR1979-23
Triplophysa robusta|YRFIR1987-23
Triplophysa robusta|YRFIR2002-23
Triplophysa robusta|YRFIR2003-23
Triplophysa robusta|YRFIR2009-23
Triplophysa robusta|YRFIR2018-23
Triplophysa robusta|YRFIR2020-23
Triplophysa robusta|YRFIR2023-23
Triplophysa robusta|YRFIR2026-23
Triplophysa robusta|YRFIR2027-23
Triplophysa robusta|YRFIR2029-23
Triplophysa robusta|YRFIR2032-23
Triplophysa robusta|YRFIR2036-23
Triplophysa robusta|YRFIR2038-23
Triplophysa robusta|YRFIR2039-23
Triplophysa robusta|YRFIR2043-23
Triplophysa robusta|YRFIR1993-23
Triplophysa robusta|YRFIR1994-23
Triplophysa robusta|YRFIR1995-23
Triplophysa robusta|YRFIR1998-23
Triplophysa robusta|YRFIR1986-23
Triplophysa robusta|YRFIR1990-23
Triplophysa robusta|YRFIR1980-23
Triplophysa robusta|YRFIR1984-23
Triplophysa robusta|YRFIR1976-23
Triplophysa robusta|YRFIR1977-23
Triplophysa robusta|YRFIR1953-23
Triplophysa robusta|YRFIR1959-23
Triplophysa robusta|YRFIR1947-23
Triplophysa robusta|YRFIR1948-23
Triplophysa robusta|YRFIR2006-23
Triplophysa robusta|YRFIR2007-23
Triplophysa robusta|YRFIR1968-23
Triplophysa robusta|YRFIR1975-23
Triplophysa robusta|YRFIR1942-23
Triplophysa robusta|YRFIR222-23
Triplophysa robusta|YRFIR221-23
Triplophysa robusta|YRFIR220-23
Triplophysa robusta|YRFIR219-23
Triplophysa robusta|YRFIR216-23
Triplophysa robusta|YRFIR1961-23
Triplophysa robusta|YRFIR1983-23
Triplophysa robusta|YRFIR2041-23
Triplophysa robusta|YRFIR2042-23
Triplophysa robusta|YRFIR2044-23
Triplophysa robusta|YRFIR2046-23
Triplophysa robusta|YRFIR1951-23
Triplophysa robusta|YRFIR1958-23
Triplophysa robusta|YRFIR2001-23
Triplophysa minxianensis|YRFIR2303-23
Triplophysa minxianensis|YRFIR2282-23
Triplophysa minxianensis|YRFIR2256-23
Triplophysa minxianensis|YRFIR2252-23
Triplophysa minxianensis|YRFIR2230-23
Triplophysa minxianensis|YRFIR2222-23
Triplophysa minxianensis|YRFIR2243-23
Triplophysa minxianensis|YRFIR2247-23
Triplophysa minxianensis|YRFIR2297-23
Triplophysa minxianensis|YRFIR2331-23
Triplophysa minxianensis|YRFIR2332-23
Triplophysa minxianensis|YRFIR2365-23
Triplophysa minxianensis|YRFIR2372-23
Triplophysa minxianensis|YRFIR2386-23

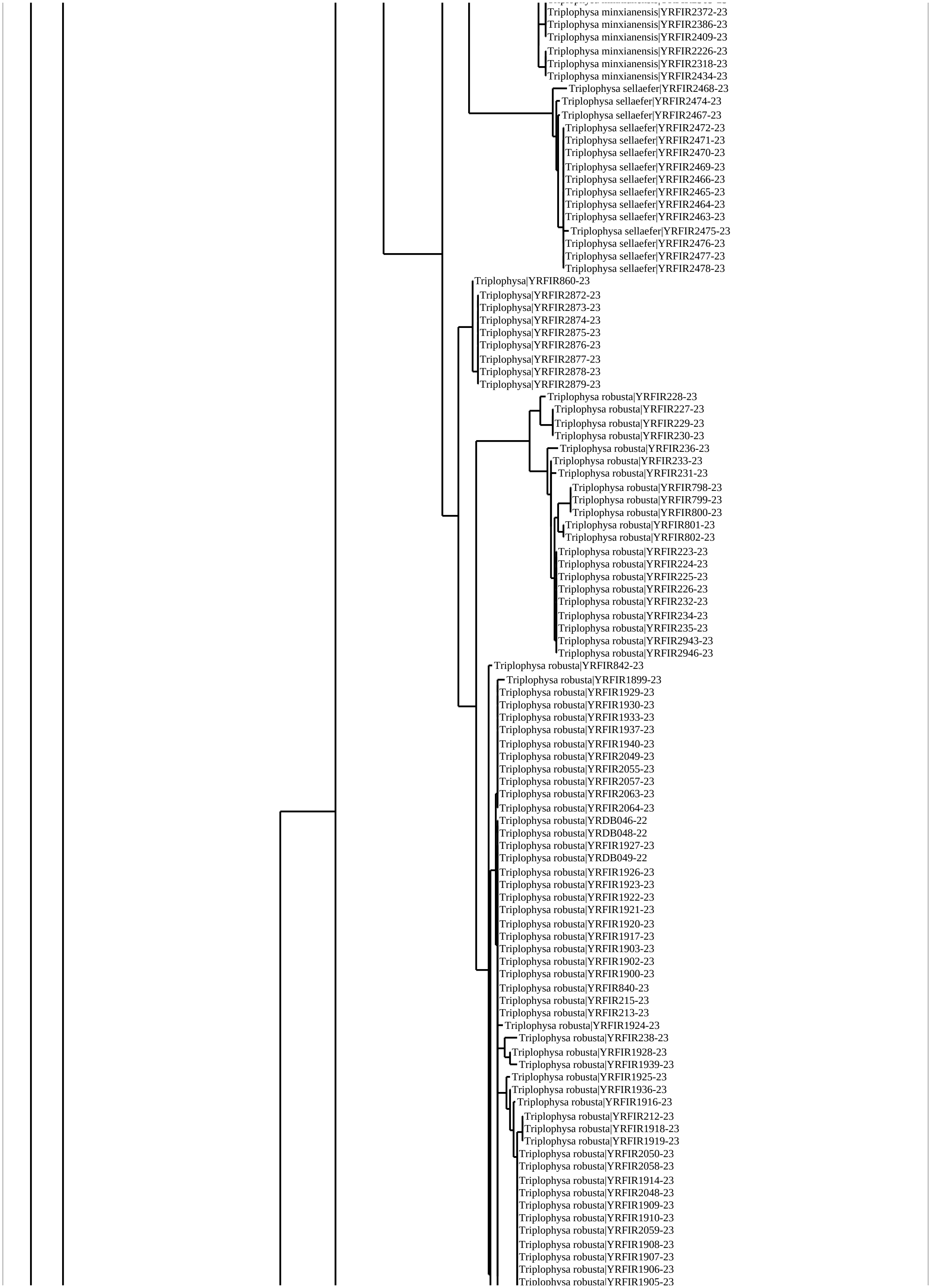
Triplophysa minxianensis|YRFIR2372-23
Triplophysa minxianensis|YRFIR2386-23
Triplophysa minxianensis|YRFIR2409-23
Triplophysa minxianensis|YRFIR2226-23
Triplophysa minxianensis|YRFIR2318-23
Triplophysa minxianensis|YRFIR2434-23
Triplophysa sellaefer|YRFIR2468-23
Triplophysa sellaefer|YRFIR2474-23
Triplophysa sellaefer|YRFIR2467-23
Triplophysa sellaefer|YRFIR2472-23
Triplophysa sellaefer|YRFIR2471-23
Triplophysa sellaefer|YRFIR2470-23
Triplophysa sellaefer|YRFIR2469-23
Triplophysa sellaefer|YRFIR2466-23
Triplophysa sellaefer|YRFIR2465-23
Triplophysa sellaefer|YRFIR2464-23
Triplophysa sellaefer|YRFIR2463-23
Triplophysa sellaefer|YRFIR2475-23
Triplophysa sellaefer|YRFIR2476-23
Triplophysa sellaefer|YRFIR2477-23
Triplophysa sellaefer|YRFIR2478-23
Triplophysa|YRFIR860-23
Triplophysa|YRFIR2872-23
Triplophysa|YRFIR2873-23
Triplophysa|YRFIR2874-23
Triplophysa|YRFIR2875-23
Triplophysa|YRFIR2876-23
Triplophysa|YRFIR2877-23
Triplophysa|YRFIR2878-23
Triplophysa|YRFIR2879-23
Triplophysa robusta|YRFIR228-23
Triplophysa robusta|YRFIR227-23
Triplophysa robusta|YRFIR229-23
Triplophysa robusta|YRFIR230-23
Triplophysa robusta|YRFIR236-23
Triplophysa robusta|YRFIR233-23
Triplophysa robusta|YRFIR231-23
Triplophysa robusta|YRFIR798-23
Triplophysa robusta|YRFIR799-23
Triplophysa robusta|YRFIR800-23
Triplophysa robusta|YRFIR801-23
Triplophysa robusta|YRFIR802-23
Triplophysa robusta|YRFIR223-23
Triplophysa robusta|YRFIR224-23
Triplophysa robusta|YRFIR225-23
Triplophysa robusta|YRFIR226-23
Triplophysa robusta|YRFIR232-23
Triplophysa robusta|YRFIR234-23
Triplophysa robusta|YRFIR235-23
Triplophysa robusta|YRFIR2943-23
Triplophysa robusta|YRFIR2946-23
Triplophysa robusta|YRFIR842-23
Triplophysa robusta|YRFIR1899-23
Triplophysa robusta|YRFIR1929-23
Triplophysa robusta|YRFIR1930-23
Triplophysa robusta|YRFIR1933-23
Triplophysa robusta|YRFIR1937-23
Triplophysa robusta|YRFIR1940-23
Triplophysa robusta|YRFIR2049-23
Triplophysa robusta|YRFIR2055-23
Triplophysa robusta|YRFIR2057-23
Triplophysa robusta|YRFIR2063-23
Triplophysa robusta|YRFIR2064-23
Triplophysa robusta|YRDB046-22
Triplophysa robusta|YRDB048-22
Triplophysa robusta|YRFIR1927-23
Triplophysa robusta|YRDB049-22
Triplophysa robusta|YRFIR1926-23
Triplophysa robusta|YRFIR1923-23
Triplophysa robusta|YRFIR1922-23
Triplophysa robusta|YRFIR1921-23
Triplophysa robusta|YRFIR1920-23
Triplophysa robusta|YRFIR1917-23
Triplophysa robusta|YRFIR1903-23
Triplophysa robusta|YRFIR1902-23
Triplophysa robusta|YRFIR1900-23
Triplophysa robusta|YRFIR840-23
Triplophysa robusta|YRFIR215-23
Triplophysa robusta|YRFIR213-23
Triplophysa robusta|YRFIR1924-23
Triplophysa robusta|YRFIR238-23
Triplophysa robusta|YRFIR1928-23
Triplophysa robusta|YRFIR1939-23
Triplophysa robusta|YRFIR1925-23
Triplophysa robusta|YRFIR1936-23
Triplophysa robusta|YRFIR1916-23
Triplophysa robusta|YRFIR212-23
Triplophysa robusta|YRFIR1918-23
Triplophysa robusta|YRFIR1919-23
Triplophysa robusta|YRFIR2050-23
Triplophysa robusta|YRFIR2058-23
Triplophysa robusta|YRFIR1914-23
Triplophysa robusta|YRFIR2048-23
Triplophysa robusta|YRFIR1909-23
Triplophysa robusta|YRFIR1910-23
Triplophysa robusta|YRFIR2059-23
Triplophysa robusta|YRFIR1908-23
Triplophysa robusta|YRFIR1907-23
Triplophysa robusta|YRFIR1906-23
Triplophysa robusta|YRFIR1905-23

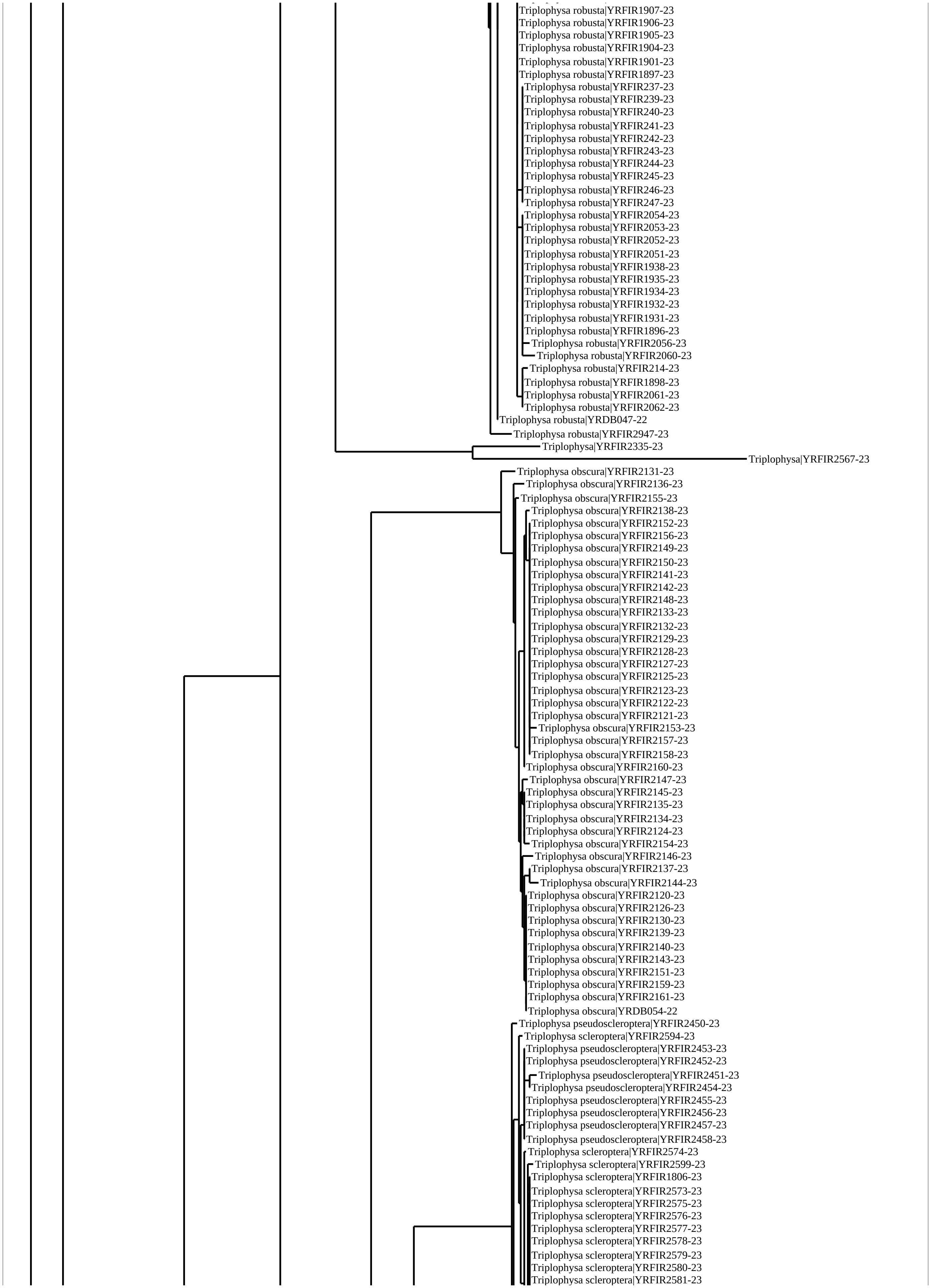
Triplophysa robusta|YRFIR1907-23
Triplophysa robusta|YRFIR1906-23
Triplophysa robusta|YRFIR1905-23
Triplophysa robusta|YRFIR1904-23
Triplophysa robusta|YRFIR1901-23
Triplophysa robusta|YRFIR1897-23
Triplophysa robusta|YRFIR237-23
Triplophysa robusta|YRFIR239-23
Triplophysa robusta|YRFIR240-23
Triplophysa robusta|YRFIR241-23
Triplophysa robusta|YRFIR242-23
Triplophysa robusta|YRFIR243-23
Triplophysa robusta|YRFIR244-23
Triplophysa robusta|YRFIR245-23
Triplophysa robusta|YRFIR246-23
Triplophysa robusta|YRFIR247-23
Triplophysa robusta|YRFIR2054-23
Triplophysa robusta|YRFIR2053-23
Triplophysa robusta|YRFIR2052-23
Triplophysa robusta|YRFIR2051-23
Triplophysa robusta|YRFIR1938-23
Triplophysa robusta|YRFIR1935-23
Triplophysa robusta|YRFIR1934-23
Triplophysa robusta|YRFIR1932-23
Triplophysa robusta|YRFIR1931-23
Triplophysa robusta|YRFIR1896-23
Triplophysa robusta|YRFIR2056-23
Triplophysa robusta|YRFIR2060-23
Triplophysa robusta|YRFIR214-23
Triplophysa robusta|YRFIR1898-23
Triplophysa robusta|YRFIR2061-23
Triplophysa robusta|YRFIR2062-23
Triplophysa robusta|YRDB047-22
Triplophysa robusta|YRFIR2947-23
Triplophysa|YRFIR2335-23
Triplophysa|YRFIR2567-23
Triplophysa obscura|YRFIR2131-23
Triplophysa obscura|YRFIR2136-23
Triplophysa obscura|YRFIR2155-23
Triplophysa obscura|YRFIR2138-23
Triplophysa obscura|YRFIR2152-23
Triplophysa obscura|YRFIR2156-23
Triplophysa obscura|YRFIR2149-23
Triplophysa obscura|YRFIR2150-23
Triplophysa obscura|YRFIR2141-23
Triplophysa obscura|YRFIR2142-23
Triplophysa obscura|YRFIR2148-23
Triplophysa obscura|YRFIR2133-23
Triplophysa obscura|YRFIR2132-23
Triplophysa obscura|YRFIR2129-23
Triplophysa obscura|YRFIR2128-23
Triplophysa obscura|YRFIR2127-23
Triplophysa obscura|YRFIR2125-23
Triplophysa obscura|YRFIR2123-23
Triplophysa obscura|YRFIR2122-23
Triplophysa obscura|YRFIR2121-23
Triplophysa obscura|YRFIR2153-23
Triplophysa obscura|YRFIR2157-23
Triplophysa obscura|YRFIR2158-23
Triplophysa obscura|YRFIR2160-23
Triplophysa obscura|YRFIR2147-23
Triplophysa obscura|YRFIR2145-23
Triplophysa obscura|YRFIR2135-23
Triplophysa obscura|YRFIR2134-23
Triplophysa obscura|YRFIR2124-23
Triplophysa obscura|YRFIR2154-23
Triplophysa obscura|YRFIR2146-23
Triplophysa obscura|YRFIR2137-23
Triplophysa obscura|YRFIR2144-23
Triplophysa obscura|YRFIR2120-23
Triplophysa obscura|YRFIR2126-23
Triplophysa obscura|YRFIR2130-23
Triplophysa obscura|YRFIR2139-23
Triplophysa obscura|YRFIR2140-23
Triplophysa obscura|YRFIR2143-23
Triplophysa obscura|YRFIR2151-23
Triplophysa obscura|YRFIR2159-23
Triplophysa obscura|YRFIR2161-23
Triplophysa obscura|YRDB054-22
Triplophysa pseudoscleroptera|YRFIR2450-23
Triplophysa scleroptera|YRFIR2594-23
Triplophysa pseudoscleroptera|YRFIR2453-23
Triplophysa pseudoscleroptera|YRFIR2452-23
Triplophysa pseudoscleroptera|YRFIR2451-23
Triplophysa pseudoscleroptera|YRFIR2454-23
Triplophysa pseudoscleroptera|YRFIR2455-23
Triplophysa pseudoscleroptera|YRFIR2456-23
Triplophysa pseudoscleroptera|YRFIR2457-23
Triplophysa pseudoscleroptera|YRFIR2458-23
Triplophysa scleroptera|YRFIR2574-23
Triplophysa scleroptera|YRFIR2599-23
Triplophysa scleroptera|YRFIR1806-23
Triplophysa scleroptera|YRFIR2573-23
Triplophysa scleroptera|YRFIR2575-23
Triplophysa scleroptera|YRFIR2576-23
Triplophysa scleroptera|YRFIR2577-23
Triplophysa scleroptera|YRFIR2578-23
Triplophysa scleroptera|YRFIR2579-23
Triplophysa scleroptera|YRFIR2580-23
Triplophysa scleroptera|YRFIR2581-23

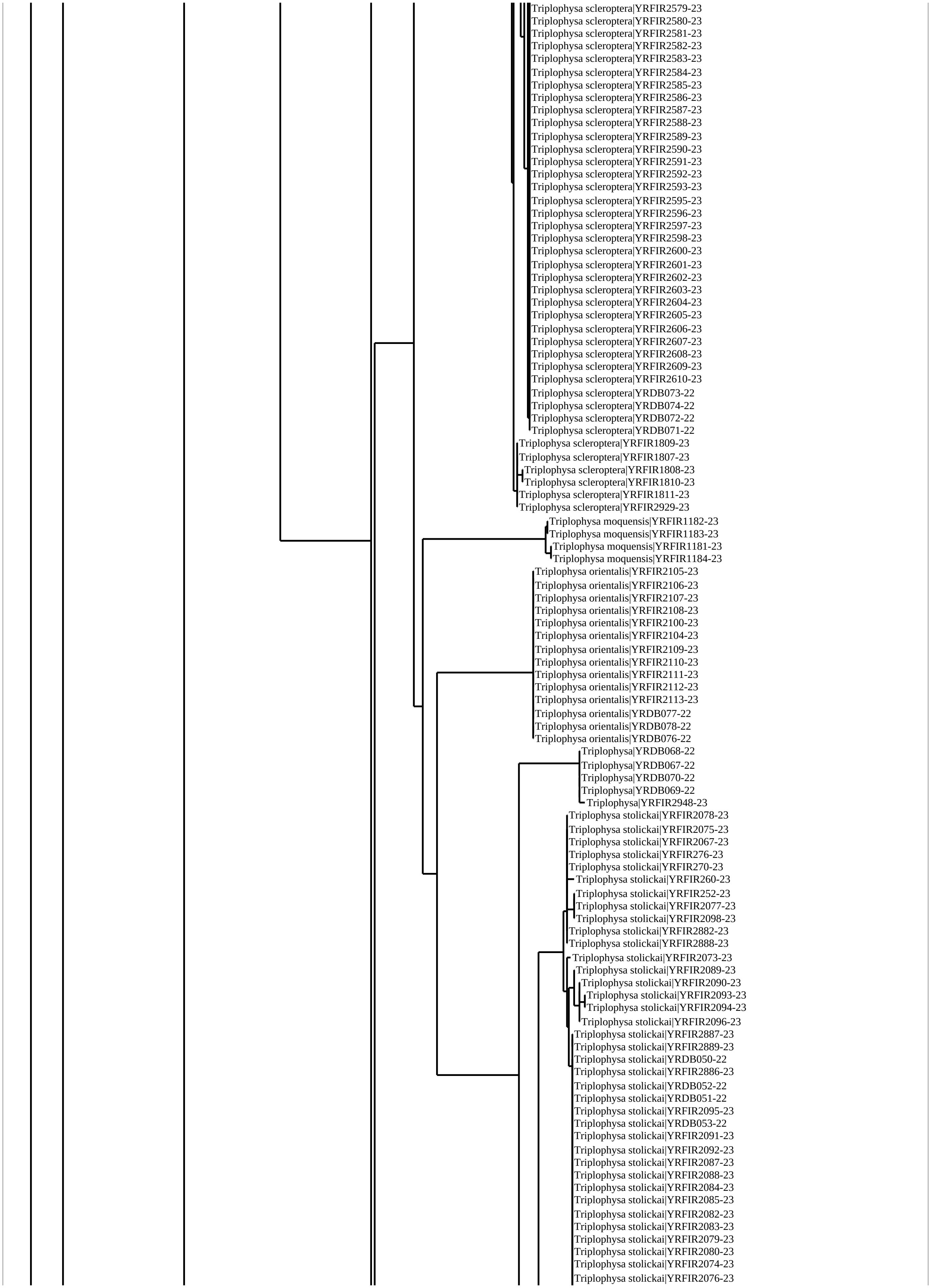
Triplophysa scleroptera|YRFIR2579-23
Triplophysa scleroptera|YRFIR2580-23
Triplophysa scleroptera|YRFIR2581-23
Triplophysa scleroptera|YRFIR2582-23
Triplophysa scleroptera|YRFIR2583-23
Triplophysa scleroptera|YRFIR2584-23
Triplophysa scleroptera|YRFIR2585-23
Triplophysa scleroptera|YRFIR2586-23
Triplophysa scleroptera|YRFIR2587-23
Triplophysa scleroptera|YRFIR2588-23
Triplophysa scleroptera|YRFIR2589-23
Triplophysa scleroptera|YRFIR2590-23
Triplophysa scleroptera|YRFIR2591-23
Triplophysa scleroptera|YRFIR2592-23
Triplophysa scleroptera|YRFIR2593-23
Triplophysa scleroptera|YRFIR2595-23
Triplophysa scleroptera|YRFIR2596-23
Triplophysa scleroptera|YRFIR2597-23
Triplophysa scleroptera|YRFIR2598-23
Triplophysa scleroptera|YRFIR2600-23
Triplophysa scleroptera|YRFIR2601-23
Triplophysa scleroptera|YRFIR2602-23
Triplophysa scleroptera|YRFIR2603-23
Triplophysa scleroptera|YRFIR2604-23
Triplophysa scleroptera|YRFIR2605-23
Triplophysa scleroptera|YRFIR2606-23
Triplophysa scleroptera|YRFIR2607-23
Triplophysa scleroptera|YRFIR2608-23
Triplophysa scleroptera|YRFIR2609-23
Triplophysa scleroptera|YRFIR2610-23
Triplophysa scleroptera|YRDB073-22
Triplophysa scleroptera|YRDB074-22
Triplophysa scleroptera|YRDB072-22
Triplophysa scleroptera|YRDB071-22
Triplophysa scleroptera|YRFIR1809-23
Triplophysa scleroptera|YRFIR1807-23
Triplophysa scleroptera|YRFIR1808-23
Triplophysa scleroptera|YRFIR1810-23
Triplophysa scleroptera|YRFIR1811-23
Triplophysa scleroptera|YRFIR2929-23
Triplophysa moquensis|YRFIR1182-23
Triplophysa moquensis|YRFIR1183-23
Triplophysa moquensis|YRFIR1181-23
Triplophysa moquensis|YRFIR1184-23
Triplophysa orientalis|YRFIR2105-23
Triplophysa orientalis|YRFIR2106-23
Triplophysa orientalis|YRFIR2107-23
Triplophysa orientalis|YRFIR2108-23
Triplophysa orientalis|YRFIR2100-23
Triplophysa orientalis|YRFIR2104-23
Triplophysa orientalis|YRFIR2109-23
Triplophysa orientalis|YRFIR2110-23
Triplophysa orientalis|YRFIR2111-23
Triplophysa orientalis|YRFIR2112-23
Triplophysa orientalis|YRFIR2113-23
Triplophysa orientalis|YRDB077-22
Triplophysa orientalis|YRDB078-22
Triplophysa orientalis|YRDB076-22
Triplophysa|YRDB068-22
Triplophysa|YRDB067-22
Triplophysa|YRDB070-22
Triplophysa|YRDB069-22
Triplophysa|YRFIR2948-23
Triplophysa stolickai|YRFIR2078-23
Triplophysa stolickai|YRFIR2075-23
Triplophysa stolickai|YRFIR2067-23
Triplophysa stolickai|YRFIR276-23
Triplophysa stolickai|YRFIR270-23
Triplophysa stolickai|YRFIR260-23
Triplophysa stolickai|YRFIR252-23
Triplophysa stolickai|YRFIR2077-23
Triplophysa stolickai|YRFIR2098-23
Triplophysa stolickai|YRFIR2882-23
Triplophysa stolickai|YRFIR2888-23
Triplophysa stolickai|YRFIR2073-23
Triplophysa stolickai|YRFIR2089-23
Triplophysa stolickai|YRFIR2090-23
Triplophysa stolickai|YRFIR2093-23
Triplophysa stolickai|YRFIR2094-23
Triplophysa stolickai|YRFIR2096-23
Triplophysa stolickai|YRFIR2887-23
Triplophysa stolickai|YRFIR2889-23
Triplophysa stolickai|YRDB050-22
Triplophysa stolickai|YRFIR2886-23
Triplophysa stolickai|YRDB052-22
Triplophysa stolickai|YRDB051-22
Triplophysa stolickai|YRFIR2095-23
Triplophysa stolickai|YRDB053-22
Triplophysa stolickai|YRFIR2091-23
Triplophysa stolickai|YRFIR2092-23
Triplophysa stolickai|YRFIR2087-23
Triplophysa stolickai|YRFIR2088-23
Triplophysa stolickai|YRFIR2084-23
Triplophysa stolickai|YRFIR2085-23
Triplophysa stolickai|YRFIR2082-23
Triplophysa stolickai|YRFIR2083-23
Triplophysa stolickai|YRFIR2079-23
Triplophysa stolickai|YRFIR2080-23
Triplophysa stolickai|YRFIR2074-23
Triplophysa stolickai|YRFIR2076-23

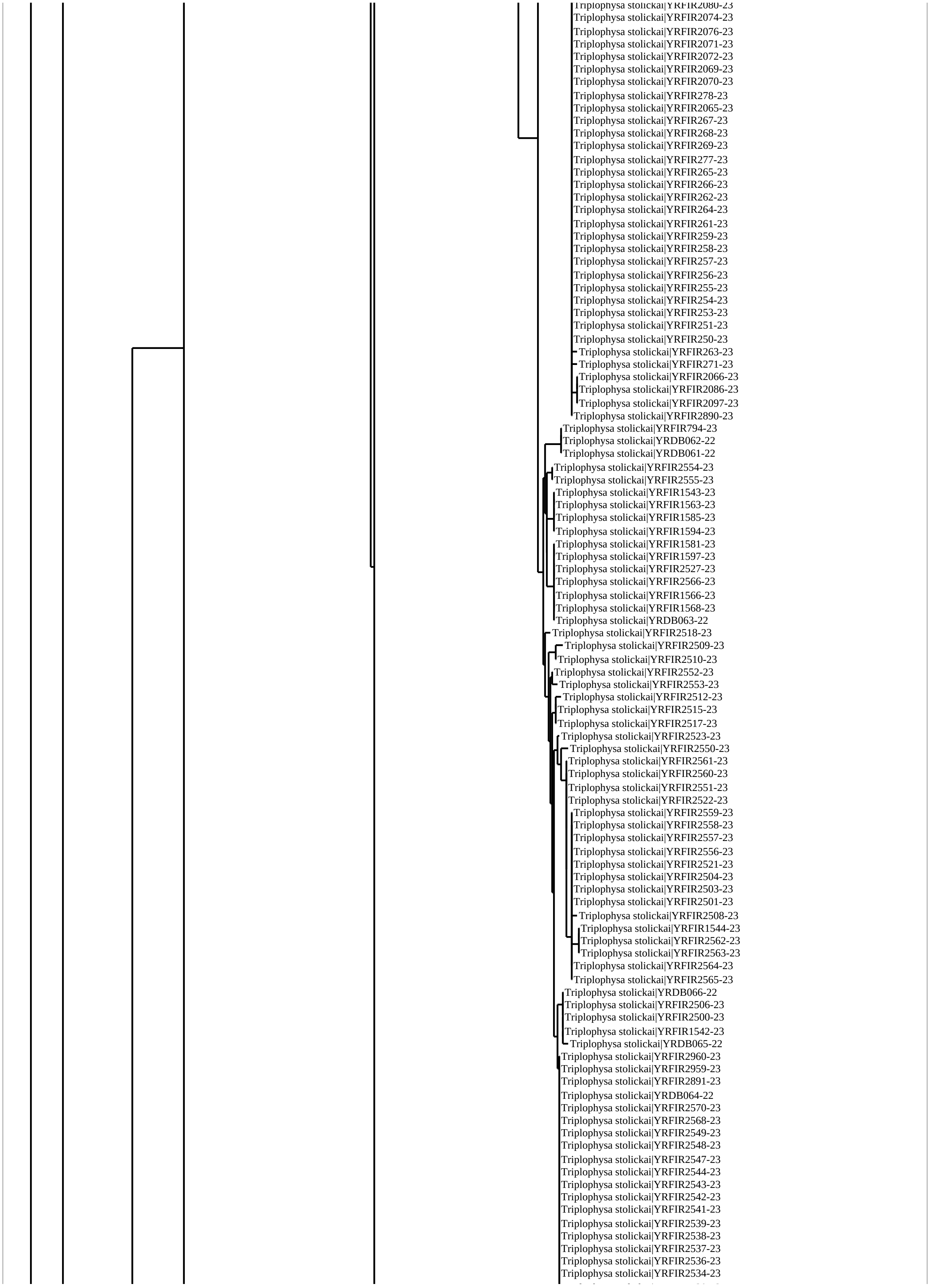
Triplophysa stolickai|YRFIR2080-23
Triplophysa stolickai|YRFIR2074-23
Triplophysa stolickai|YRFIR2076-23
Triplophysa stolickai|YRFIR2071-23
Triplophysa stolickai|YRFIR2072-23
Triplophysa stolickai|YRFIR2069-23
Triplophysa stolickai|YRFIR2070-23
Triplophysa stolickai|YRFIR278-23
Triplophysa stolickai|YRFIR2065-23
Triplophysa stolickai|YRFIR267-23
Triplophysa stolickai|YRFIR268-23
Triplophysa stolickai|YRFIR269-23
Triplophysa stolickai|YRFIR277-23
Triplophysa stolickai|YRFIR265-23
Triplophysa stolickai|YRFIR266-23
Triplophysa stolickai|YRFIR262-23
Triplophysa stolickai|YRFIR264-23
Triplophysa stolickai|YRFIR261-23
Triplophysa stolickai|YRFIR259-23
Triplophysa stolickai|YRFIR258-23
Triplophysa stolickai|YRFIR257-23
Triplophysa stolickai|YRFIR256-23
Triplophysa stolickai|YRFIR255-23
Triplophysa stolickai|YRFIR254-23
Triplophysa stolickai|YRFIR253-23
Triplophysa stolickai|YRFIR251-23
Triplophysa stolickai|YRFIR250-23
Triplophysa stolickai|YRFIR263-23
Triplophysa stolickai|YRFIR271-23
Triplophysa stolickai|YRFIR2066-23
Triplophysa stolickai|YRFIR2086-23
Triplophysa stolickai|YRFIR2097-23
Triplophysa stolickai|YRFIR2890-23
Triplophysa stolickai|YRFIR794-23
Triplophysa stolickai|YRDB062-22
Triplophysa stolickai|YRDB061-22
Triplophysa stolickai|YRFIR2554-23
Triplophysa stolickai|YRFIR2555-23
Triplophysa stolickai|YRFIR1543-23
Triplophysa stolickai|YRFIR1563-23
Triplophysa stolickai|YRFIR1585-23
Triplophysa stolickai|YRFIR1594-23
Triplophysa stolickai|YRFIR1581-23
Triplophysa stolickai|YRFIR1597-23
Triplophysa stolickai|YRFIR2527-23
Triplophysa stolickai|YRFIR2566-23
Triplophysa stolickai|YRFIR1566-23
Triplophysa stolickai|YRFIR1568-23
Triplophysa stolickai|YRDB063-22
Triplophysa stolickai|YRFIR2518-23
Triplophysa stolickai|YRFIR2509-23
Triplophysa stolickai|YRFIR2510-23
Triplophysa stolickai|YRFIR2552-23
Triplophysa stolickai|YRFIR2553-23
Triplophysa stolickai|YRFIR2512-23
Triplophysa stolickai|YRFIR2515-23
Triplophysa stolickai|YRFIR2517-23
Triplophysa stolickai|YRFIR2523-23
Triplophysa stolickai|YRFIR2550-23
Triplophysa stolickai|YRFIR2561-23
Triplophysa stolickai|YRFIR2560-23
Triplophysa stolickai|YRFIR2551-23
Triplophysa stolickai|YRFIR2522-23
Triplophysa stolickai|YRFIR2559-23
Triplophysa stolickai|YRFIR2558-23
Triplophysa stolickai|YRFIR2557-23
Triplophysa stolickai|YRFIR2556-23
Triplophysa stolickai|YRFIR2521-23
Triplophysa stolickai|YRFIR2504-23
Triplophysa stolickai|YRFIR2503-23
Triplophysa stolickai|YRFIR2501-23
Triplophysa stolickai|YRFIR2508-23
Triplophysa stolickai|YRFIR1544-23
Triplophysa stolickai|YRFIR2562-23
Triplophysa stolickai|YRFIR2563-23
Triplophysa stolickai|YRFIR2564-23
Triplophysa stolickai|YRFIR2565-23
Triplophysa stolickai|YRDB066-22
Triplophysa stolickai|YRFIR2506-23
Triplophysa stolickai|YRFIR2500-23
Triplophysa stolickai|YRFIR1542-23
Triplophysa stolickai|YRDB065-22
Triplophysa stolickai|YRFIR2960-23
Triplophysa stolickai|YRFIR2959-23
Triplophysa stolickai|YRFIR2891-23
Triplophysa stolickai|YRDB064-22
Triplophysa stolickai|YRFIR2570-23
Triplophysa stolickai|YRFIR2568-23
Triplophysa stolickai|YRFIR2549-23
Triplophysa stolickai|YRFIR2548-23
Triplophysa stolickai|YRFIR2547-23
Triplophysa stolickai|YRFIR2544-23
Triplophysa stolickai|YRFIR2543-23
Triplophysa stolickai|YRFIR2542-23
Triplophysa stolickai|YRFIR2541-23
Triplophysa stolickai|YRFIR2539-23
Triplophysa stolickai|YRFIR2538-23
Triplophysa stolickai|YRFIR2537-23
Triplophysa stolickai|YRFIR2536-23
Triplophysa stolickai|YRFIR2534-23

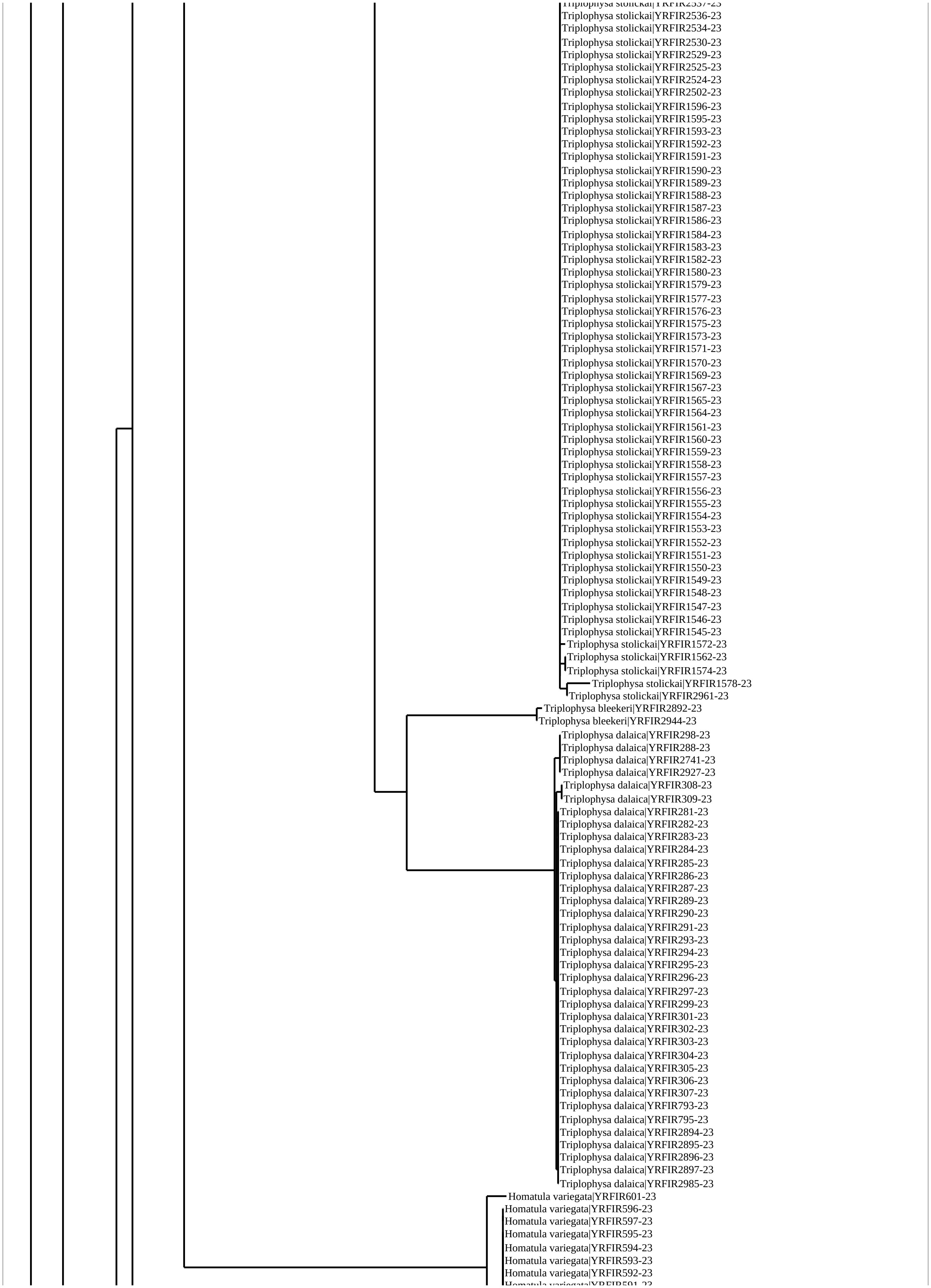
Triplophysa stolickai|YRFIR2536-23
Triplophysa stolickai|YRFIR2534-23
Triplophysa stolickai|YRFIR2530-23
Triplophysa stolickai|YRFIR2529-23
Triplophysa stolickai|YRFIR2525-23
Triplophysa stolickai|YRFIR2524-23
Triplophysa stolickai|YRFIR2502-23
Triplophysa stolickai|YRFIR1596-23
Triplophysa stolickai|YRFIR1595-23
Triplophysa stolickai|YRFIR1593-23
Triplophysa stolickai|YRFIR1592-23
Triplophysa stolickai|YRFIR1591-23
Triplophysa stolickai|YRFIR1590-23
Triplophysa stolickai|YRFIR1589-23
Triplophysa stolickai|YRFIR1588-23
Triplophysa stolickai|YRFIR1587-23
Triplophysa stolickai|YRFIR1586-23
Triplophysa stolickai|YRFIR1584-23
Triplophysa stolickai|YRFIR1583-23
Triplophysa stolickai|YRFIR1582-23
Triplophysa stolickai|YRFIR1580-23
Triplophysa stolickai|YRFIR1579-23
Triplophysa stolickai|YRFIR1577-23
Triplophysa stolickai|YRFIR1576-23
Triplophysa stolickai|YRFIR1575-23
Triplophysa stolickai|YRFIR1573-23
Triplophysa stolickai|YRFIR1571-23
Triplophysa stolickai|YRFIR1570-23
Triplophysa stolickai|YRFIR1569-23
Triplophysa stolickai|YRFIR1567-23
Triplophysa stolickai|YRFIR1565-23
Triplophysa stolickai|YRFIR1564-23
Triplophysa stolickai|YRFIR1561-23
Triplophysa stolickai|YRFIR1560-23
Triplophysa stolickai|YRFIR1559-23
Triplophysa stolickai|YRFIR1558-23
Triplophysa stolickai|YRFIR1557-23
Triplophysa stolickai|YRFIR1556-23
Triplophysa stolickai|YRFIR1555-23
Triplophysa stolickai|YRFIR1554-23
Triplophysa stolickai|YRFIR1553-23
Triplophysa stolickai|YRFIR1552-23
Triplophysa stolickai|YRFIR1551-23
Triplophysa stolickai|YRFIR1550-23
Triplophysa stolickai|YRFIR1549-23
Triplophysa stolickai|YRFIR1548-23
Triplophysa stolickai|YRFIR1547-23
Triplophysa stolickai|YRFIR1546-23
Triplophysa stolickai|YRFIR1545-23
Triplophysa stolickai|YRFIR1572-23
Triplophysa stolickai|YRFIR1562-23
Triplophysa stolickai|YRFIR1574-23
Triplophysa stolickai|YRFIR1578-23
Triplophysa stolickai|YRFIR2961-23
Triplophysa bleekeri|YRFIR2892-23
Triplophysa bleekeri|YRFIR2944-23
Triplophysa dalaica|YRFIR298-23
Triplophysa dalaica|YRFIR288-23
Triplophysa dalaica|YRFIR2741-23
Triplophysa dalaica|YRFIR2927-23
Triplophysa dalaica|YRFIR308-23
Triplophysa dalaica|YRFIR309-23
Triplophysa dalaica|YRFIR281-23
Triplophysa dalaica|YRFIR282-23
Triplophysa dalaica|YRFIR283-23
Triplophysa dalaica|YRFIR284-23
Triplophysa dalaica|YRFIR285-23
Triplophysa dalaica|YRFIR286-23
Triplophysa dalaica|YRFIR287-23
Triplophysa dalaica|YRFIR289-23
Triplophysa dalaica|YRFIR290-23
Triplophysa dalaica|YRFIR291-23
Triplophysa dalaica|YRFIR293-23
Triplophysa dalaica|YRFIR294-23
Triplophysa dalaica|YRFIR295-23
Triplophysa dalaica|YRFIR296-23
Triplophysa dalaica|YRFIR297-23
Triplophysa dalaica|YRFIR299-23
Triplophysa dalaica|YRFIR301-23
Triplophysa dalaica|YRFIR302-23
Triplophysa dalaica|YRFIR303-23
Triplophysa dalaica|YRFIR304-23
Triplophysa dalaica|YRFIR305-23
Triplophysa dalaica|YRFIR306-23
Triplophysa dalaica|YRFIR307-23
Triplophysa dalaica|YRFIR793-23
Triplophysa dalaica|YRFIR795-23
Triplophysa dalaica|YRFIR2894-23
Triplophysa dalaica|YRFIR2895-23
Triplophysa dalaica|YRFIR2896-23
Triplophysa dalaica|YRFIR2897-23
Triplophysa dalaica|YRFIR2985-23
Homatula variegata|YRFIR601-23
Homatula variegata|YRFIR596-23
Homatula variegata|YRFIR597-23
Homatula variegata|YRFIR595-23
Homatula variegata|YRFIR594-23
Homatula variegata|YRFIR593-23
Homatula variegata|YRFIR592-23

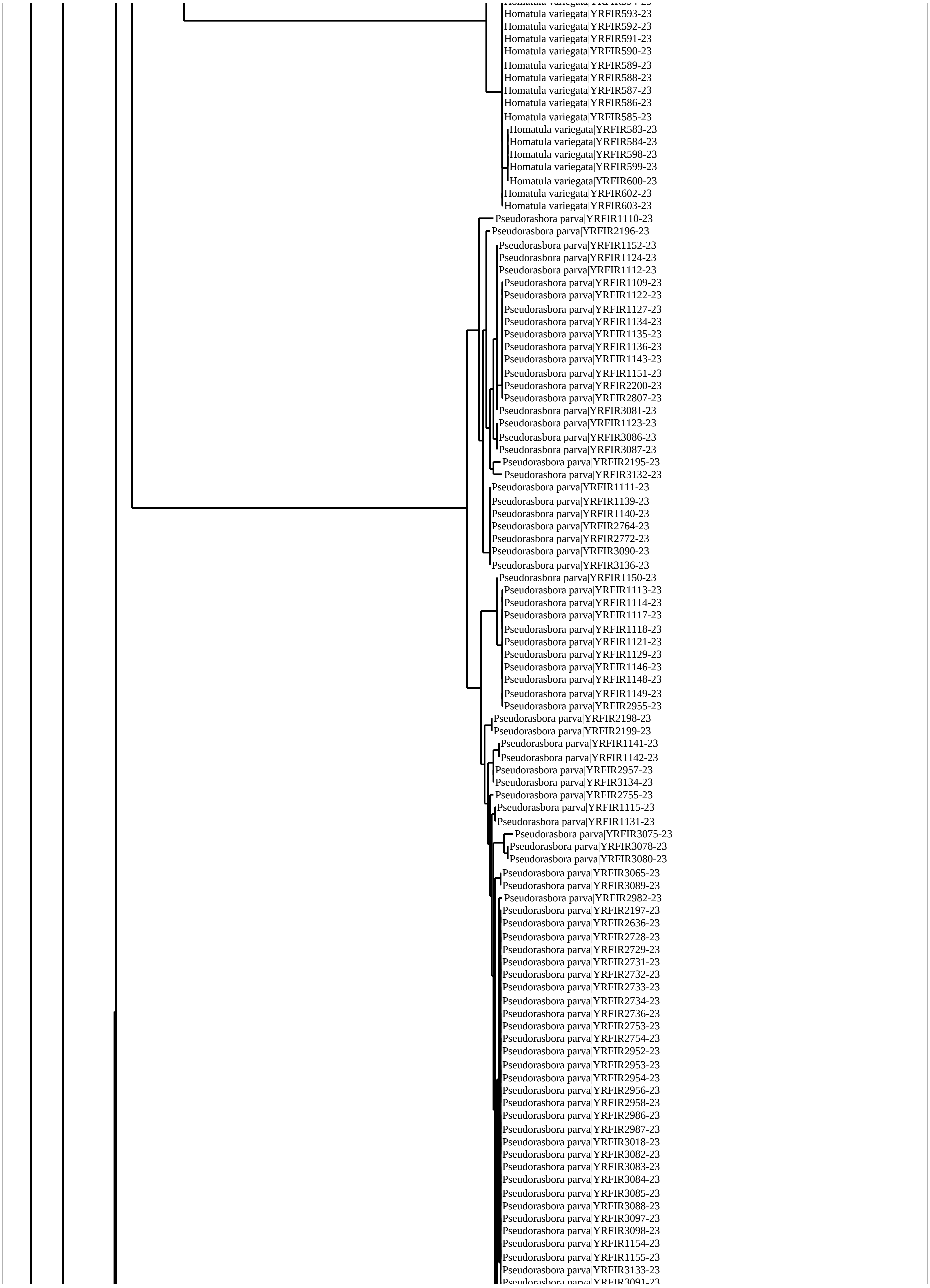
Homatula variegata|YRFIR593-23
Homatula variegata|YRFIR592-23
Homatula variegata|YRFIR591-23
Homatula variegata|YRFIR590-23
Homatula variegata|YRFIR589-23
Homatula variegata|YRFIR588-23
Homatula variegata|YRFIR587-23
Homatula variegata|YRFIR586-23
Homatula variegata|YRFIR585-23
Homatula variegata|YRFIR583-23
Homatula variegata|YRFIR584-23
Homatula variegata|YRFIR598-23
Homatula variegata|YRFIR599-23
Homatula variegata|YRFIR600-23
Homatula variegata|YRFIR602-23
Homatula variegata|YRFIR603-23
Pseudorasbora parva|YRFIR1110-23
Pseudorasbora parva|YRFIR2196-23
Pseudorasbora parva|YRFIR1152-23
Pseudorasbora parva|YRFIR1124-23
Pseudorasbora parva|YRFIR1112-23
Pseudorasbora parva|YRFIR1109-23
Pseudorasbora parva|YRFIR1122-23
Pseudorasbora parva|YRFIR1127-23
Pseudorasbora parva|YRFIR1134-23
Pseudorasbora parva|YRFIR1135-23
Pseudorasbora parva|YRFIR1136-23
Pseudorasbora parva|YRFIR1143-23
Pseudorasbora parva|YRFIR1151-23
Pseudorasbora parva|YRFIR2200-23
Pseudorasbora parva|YRFIR2807-23
Pseudorasbora parva|YRFIR3081-23
Pseudorasbora parva|YRFIR1123-23
Pseudorasbora parva|YRFIR3086-23
Pseudorasbora parva|YRFIR3087-23
Pseudorasbora parva|YRFIR2195-23
Pseudorasbora parva|YRFIR3132-23
Pseudorasbora parva|YRFIR1111-23
Pseudorasbora parva|YRFIR1139-23
Pseudorasbora parva|YRFIR1140-23
Pseudorasbora parva|YRFIR2764-23
Pseudorasbora parva|YRFIR2772-23
Pseudorasbora parva|YRFIR3090-23
Pseudorasbora parva|YRFIR3136-23
Pseudorasbora parva|YRFIR1150-23
Pseudorasbora parva|YRFIR1113-23
Pseudorasbora parva|YRFIR1114-23
Pseudorasbora parva|YRFIR1117-23
Pseudorasbora parva|YRFIR1118-23
Pseudorasbora parva|YRFIR1121-23
Pseudorasbora parva|YRFIR1129-23
Pseudorasbora parva|YRFIR1146-23
Pseudorasbora parva|YRFIR1148-23
Pseudorasbora parva|YRFIR1149-23
Pseudorasbora parva|YRFIR2955-23
Pseudorasbora parva|YRFIR2198-23
Pseudorasbora parva|YRFIR2199-23
Pseudorasbora parva|YRFIR1141-23
Pseudorasbora parva|YRFIR1142-23
Pseudorasbora parva|YRFIR2957-23
Pseudorasbora parva|YRFIR3134-23
Pseudorasbora parva|YRFIR2755-23
Pseudorasbora parva|YRFIR1115-23
Pseudorasbora parva|YRFIR1131-23
Pseudorasbora parva|YRFIR3075-23
Pseudorasbora parva|YRFIR3078-23
Pseudorasbora parva|YRFIR3080-23
Pseudorasbora parva|YRFIR3065-23
Pseudorasbora parva|YRFIR3089-23
Pseudorasbora parva|YRFIR2982-23
Pseudorasbora parva|YRFIR2197-23
Pseudorasbora parva|YRFIR2636-23
Pseudorasbora parva|YRFIR2728-23
Pseudorasbora parva|YRFIR2729-23
Pseudorasbora parva|YRFIR2731-23
Pseudorasbora parva|YRFIR2732-23
Pseudorasbora parva|YRFIR2733-23
Pseudorasbora parva|YRFIR2734-23
Pseudorasbora parva|YRFIR2736-23
Pseudorasbora parva|YRFIR2753-23
Pseudorasbora parva|YRFIR2754-23
Pseudorasbora parva|YRFIR2952-23
Pseudorasbora parva|YRFIR2953-23
Pseudorasbora parva|YRFIR2954-23
Pseudorasbora parva|YRFIR2956-23
Pseudorasbora parva|YRFIR2958-23
Pseudorasbora parva|YRFIR2986-23
Pseudorasbora parva|YRFIR2987-23
Pseudorasbora parva|YRFIR3018-23
Pseudorasbora parva|YRFIR3082-23
Pseudorasbora parva|YRFIR3083-23
Pseudorasbora parva|YRFIR3084-23
Pseudorasbora parva|YRFIR3085-23
Pseudorasbora parva|YRFIR3088-23
Pseudorasbora parva|YRFIR3097-23
Pseudorasbora parva|YRFIR3098-23
Pseudorasbora parva|YRFIR1154-23
Pseudorasbora parva|YRFIR1155-23
Pseudorasbora parva|YRFIR3133-23
Pseudorasbora parva|YRFIR3091-23

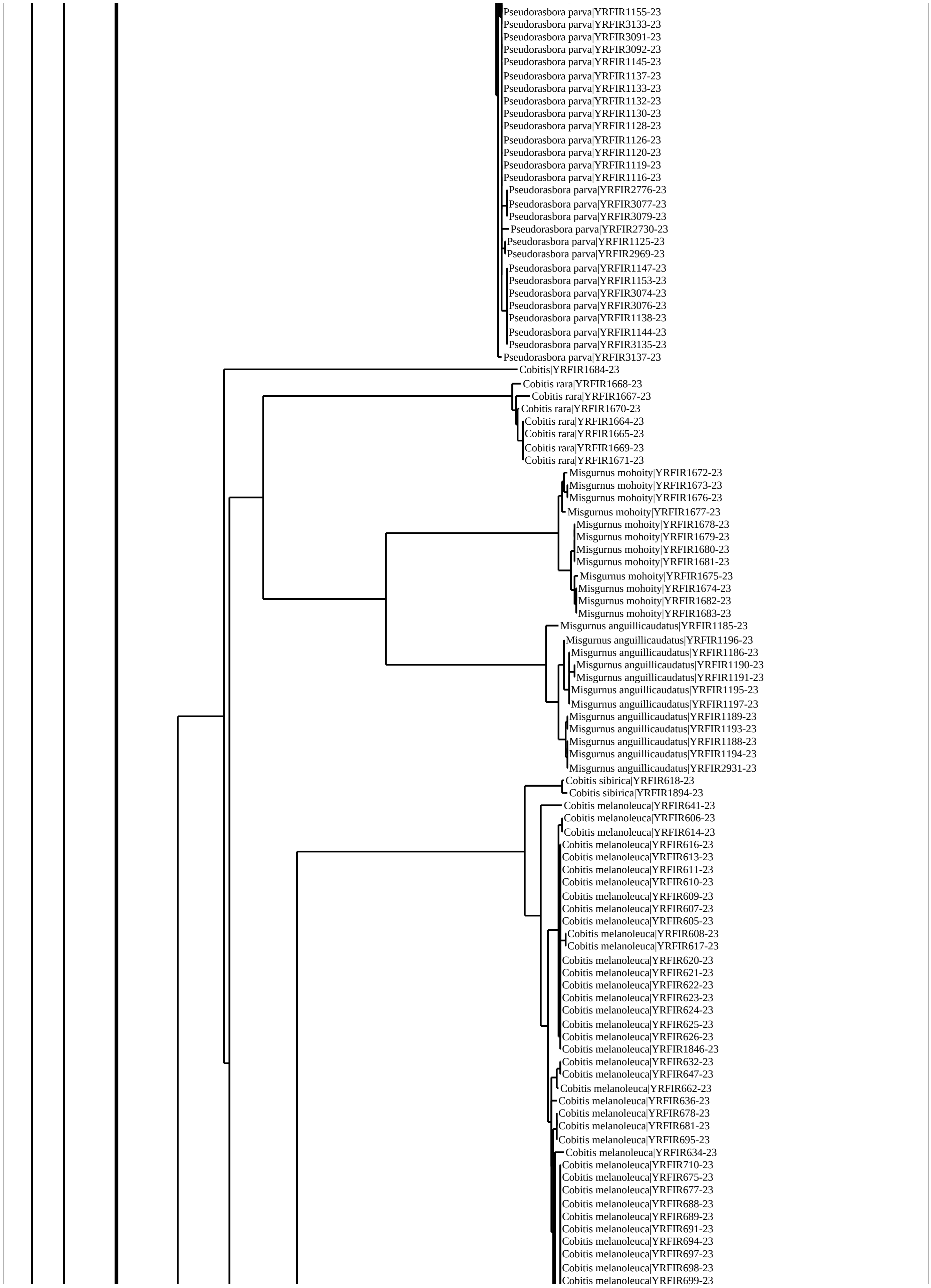
Pseudorasbora parva|YRFIR1155-23
Pseudorasbora parva|YRFIR3133-23
Pseudorasbora parva|YRFIR3091-23
Pseudorasbora parva|YRFIR3092-23
Pseudorasbora parva|YRFIR1145-23
Pseudorasbora parva|YRFIR1137-23
Pseudorasbora parva|YRFIR1133-23
Pseudorasbora parva|YRFIR1132-23
Pseudorasbora parva|YRFIR1130-23
Pseudorasbora parva|YRFIR1128-23
Pseudorasbora parva|YRFIR1126-23
Pseudorasbora parva|YRFIR1120-23
Pseudorasbora parva|YRFIR1119-23
Pseudorasbora parva|YRFIR1116-23
Pseudorasbora parva|YRFIR2776-23
Pseudorasbora parva|YRFIR3077-23
Pseudorasbora parva|YRFIR3079-23
Pseudorasbora parva|YRFIR2730-23
Pseudorasbora parva|YRFIR1125-23
Pseudorasbora parva|YRFIR2969-23
Pseudorasbora parva|YRFIR1147-23
Pseudorasbora parva|YRFIR1153-23
Pseudorasbora parva|YRFIR3074-23
Pseudorasbora parva|YRFIR3076-23
Pseudorasbora parva|YRFIR1138-23
Pseudorasbora parva|YRFIR1144-23
Pseudorasbora parva|YRFIR3135-23
Pseudorasbora parva|YRFIR3137-23
Cobitis|YRFIR1684-23
Cobitis rara|YRFIR1668-23
Cobitis rara|YRFIR1667-23
Cobitis rara|YRFIR1670-23
Cobitis rara|YRFIR1664-23
Cobitis rara|YRFIR1665-23
Cobitis rara|YRFIR1669-23
Cobitis rara|YRFIR1671-23
Misgurnus mohoity|YRFIR1672-23
Misgurnus mohoity|YRFIR1673-23
Misgurnus mohoity|YRFIR1676-23
Misgurnus mohoity|YRFIR1677-23
Misgurnus mohoity|YRFIR1678-23
Misgurnus mohoity|YRFIR1679-23
Misgurnus mohoity|YRFIR1680-23
Misgurnus mohoity|YRFIR1681-23
Misgurnus mohoity|YRFIR1675-23
Misgurnus mohoity|YRFIR1674-23
Misgurnus mohoity|YRFIR1682-23
Misgurnus mohoity|YRFIR1683-23
Misgurnus anguillicaudatus|YRFIR1185-23
Misgurnus anguillicaudatus|YRFIR1196-23
Misgurnus anguillicaudatus|YRFIR1186-23
Misgurnus anguillicaudatus|YRFIR1190-23
Misgurnus anguillicaudatus|YRFIR1191-23
Misgurnus anguillicaudatus|YRFIR1195-23
Misgurnus anguillicaudatus|YRFIR1197-23
Misgurnus anguillicaudatus|YRFIR1189-23
Misgurnus anguillicaudatus|YRFIR1193-23
Misgurnus anguillicaudatus|YRFIR1188-23
Misgurnus anguillicaudatus|YRFIR1194-23
Misgurnus anguillicaudatus|YRFIR2931-23
Cobitis sibirica|YRFIR618-23
Cobitis sibirica|YRFIR1894-23
Cobitis melanoleuca|YRFIR641-23
Cobitis melanoleuca|YRFIR606-23
Cobitis melanoleuca|YRFIR614-23
Cobitis melanoleuca|YRFIR616-23
Cobitis melanoleuca|YRFIR613-23
Cobitis melanoleuca|YRFIR611-23
Cobitis melanoleuca|YRFIR610-23
Cobitis melanoleuca|YRFIR609-23
Cobitis melanoleuca|YRFIR607-23
Cobitis melanoleuca|YRFIR605-23
Cobitis melanoleuca|YRFIR608-23
Cobitis melanoleuca|YRFIR617-23
Cobitis melanoleuca|YRFIR620-23
Cobitis melanoleuca|YRFIR621-23
Cobitis melanoleuca|YRFIR622-23
Cobitis melanoleuca|YRFIR623-23
Cobitis melanoleuca|YRFIR624-23
Cobitis melanoleuca|YRFIR625-23
Cobitis melanoleuca|YRFIR626-23
Cobitis melanoleuca|YRFIR1846-23
Cobitis melanoleuca|YRFIR632-23
Cobitis melanoleuca|YRFIR647-23
Cobitis melanoleuca|YRFIR662-23
Cobitis melanoleuca|YRFIR636-23
Cobitis melanoleuca|YRFIR678-23
Cobitis melanoleuca|YRFIR681-23
Cobitis melanoleuca|YRFIR695-23
Cobitis melanoleuca|YRFIR634-23
Cobitis melanoleuca|YRFIR710-23
Cobitis melanoleuca|YRFIR675-23
Cobitis melanoleuca|YRFIR677-23
Cobitis melanoleuca|YRFIR688-23
Cobitis melanoleuca|YRFIR689-23
Cobitis melanoleuca|YRFIR691-23
Cobitis melanoleuca|YRFIR694-23
Cobitis melanoleuca|YRFIR697-23
Cobitis melanoleuca|YRFIR698-23
Cobitis melanoleuca|YRFIR699-23

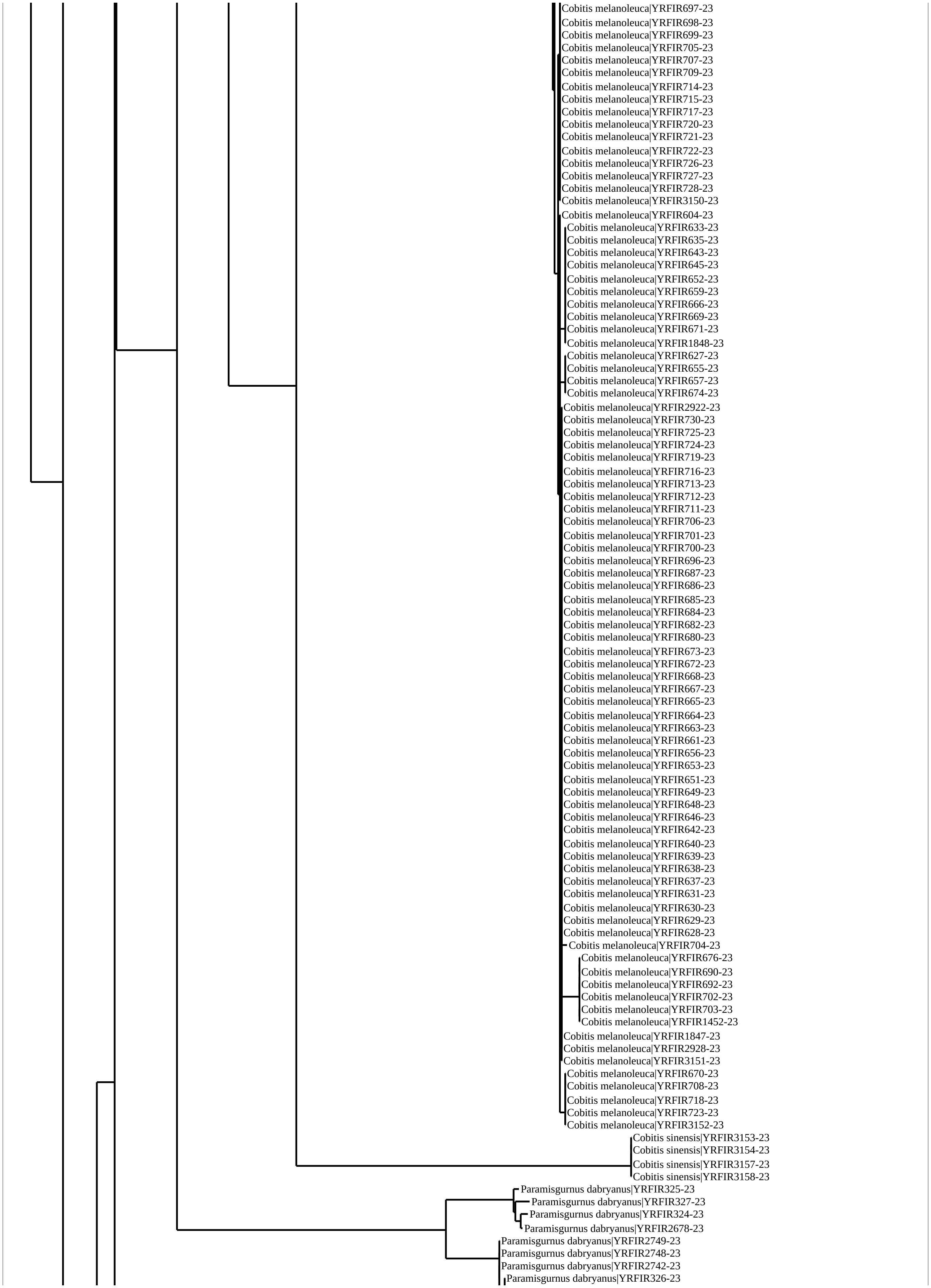
Cobitis melanoleuca|YRFIR697-23
Cobitis melanoleuca|YRFIR698-23
Cobitis melanoleuca|YRFIR699-23
Cobitis melanoleuca|YRFIR705-23
Cobitis melanoleuca|YRFIR707-23
Cobitis melanoleuca|YRFIR709-23
Cobitis melanoleuca|YRFIR714-23
Cobitis melanoleuca|YRFIR715-23
Cobitis melanoleuca|YRFIR717-23
Cobitis melanoleuca|YRFIR720-23
Cobitis melanoleuca|YRFIR721-23
Cobitis melanoleuca|YRFIR722-23
Cobitis melanoleuca|YRFIR726-23
Cobitis melanoleuca|YRFIR727-23
Cobitis melanoleuca|YRFIR728-23
Cobitis melanoleuca|YRFIR3150-23
Cobitis melanoleuca|YRFIR604-23
Cobitis melanoleuca|YRFIR633-23
Cobitis melanoleuca|YRFIR635-23
Cobitis melanoleuca|YRFIR643-23
Cobitis melanoleuca|YRFIR645-23
Cobitis melanoleuca|YRFIR652-23
Cobitis melanoleuca|YRFIR659-23
Cobitis melanoleuca|YRFIR666-23
Cobitis melanoleuca|YRFIR669-23
Cobitis melanoleuca|YRFIR671-23
Cobitis melanoleuca|YRFIR1848-23
Cobitis melanoleuca|YRFIR627-23
Cobitis melanoleuca|YRFIR655-23
Cobitis melanoleuca|YRFIR657-23
Cobitis melanoleuca|YRFIR674-23
Cobitis melanoleuca|YRFIR2922-23
Cobitis melanoleuca|YRFIR730-23
Cobitis melanoleuca|YRFIR725-23
Cobitis melanoleuca|YRFIR724-23
Cobitis melanoleuca|YRFIR719-23
Cobitis melanoleuca|YRFIR716-23
Cobitis melanoleuca|YRFIR713-23
Cobitis melanoleuca|YRFIR712-23
Cobitis melanoleuca|YRFIR711-23
Cobitis melanoleuca|YRFIR706-23
Cobitis melanoleuca|YRFIR701-23
Cobitis melanoleuca|YRFIR700-23
Cobitis melanoleuca|YRFIR696-23
Cobitis melanoleuca|YRFIR687-23
Cobitis melanoleuca|YRFIR686-23
Cobitis melanoleuca|YRFIR685-23
Cobitis melanoleuca|YRFIR684-23
Cobitis melanoleuca|YRFIR682-23
Cobitis melanoleuca|YRFIR680-23
Cobitis melanoleuca|YRFIR673-23
Cobitis melanoleuca|YRFIR672-23
Cobitis melanoleuca|YRFIR668-23
Cobitis melanoleuca|YRFIR667-23
Cobitis melanoleuca|YRFIR665-23
Cobitis melanoleuca|YRFIR664-23
Cobitis melanoleuca|YRFIR663-23
Cobitis melanoleuca|YRFIR661-23
Cobitis melanoleuca|YRFIR656-23
Cobitis melanoleuca|YRFIR653-23
Cobitis melanoleuca|YRFIR651-23
Cobitis melanoleuca|YRFIR649-23
Cobitis melanoleuca|YRFIR648-23
Cobitis melanoleuca|YRFIR646-23
Cobitis melanoleuca|YRFIR642-23
Cobitis melanoleuca|YRFIR640-23
Cobitis melanoleuca|YRFIR639-23
Cobitis melanoleuca|YRFIR638-23
Cobitis melanoleuca|YRFIR637-23
Cobitis melanoleuca|YRFIR631-23
Cobitis melanoleuca|YRFIR630-23
Cobitis melanoleuca|YRFIR629-23
Cobitis melanoleuca|YRFIR628-23
Cobitis melanoleuca|YRFIR704-23
Cobitis melanoleuca|YRFIR676-23
Cobitis melanoleuca|YRFIR690-23
Cobitis melanoleuca|YRFIR692-23
Cobitis melanoleuca|YRFIR702-23
Cobitis melanoleuca|YRFIR703-23
Cobitis melanoleuca|YRFIR1452-23
Cobitis melanoleuca|YRFIR1847-23
Cobitis melanoleuca|YRFIR2928-23
Cobitis melanoleuca|YRFIR3151-23
Cobitis melanoleuca|YRFIR670-23
Cobitis melanoleuca|YRFIR708-23
Cobitis melanoleuca|YRFIR718-23
Cobitis melanoleuca|YRFIR723-23
Cobitis melanoleuca|YRFIR3152-23
Cobitis sinensis|YRFIR3153-23
Cobitis sinensis|YRFIR3154-23
Cobitis sinensis|YRFIR3157-23
Cobitis sinensis|YRFIR3158-23
Paramisgurnus dabryanus|YRFIR325-23
Paramisgurnus dabryanus|YRFIR327-23
Paramisgurnus dabryanus|YRFIR324-23
Paramisgurnus dabryanus|YRFIR2678-23
Paramisgurnus dabryanus|YRFIR2749-23
Paramisgurnus dabryanus|YRFIR2748-23
Paramisgurnus dabryanus|YRFIR2742-23
Paramisgurnus dabryanus|YRFIR326-23

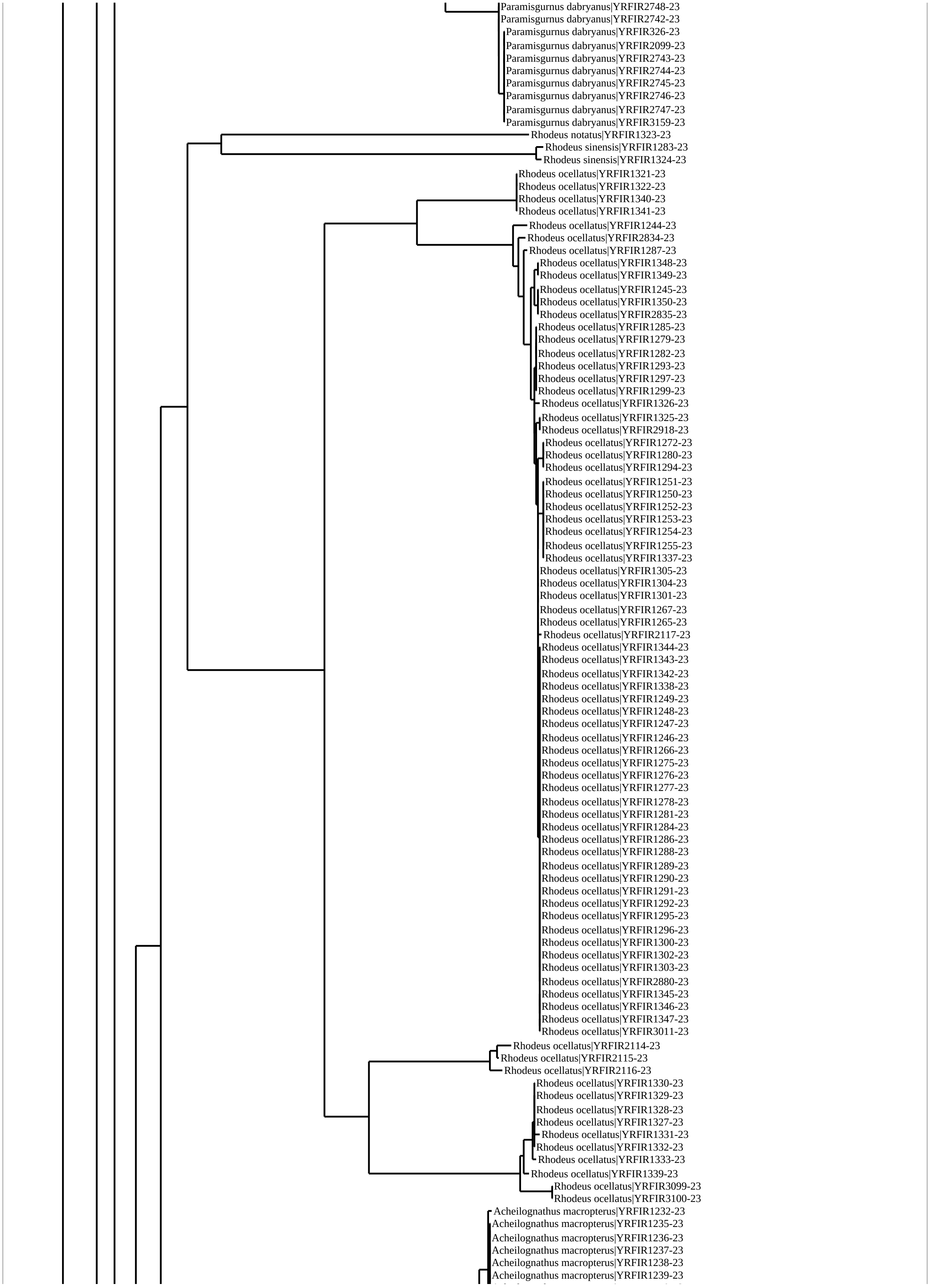
Paramisgurnus dabryanus|YRFIR2748-23
Paramisgurnus dabryanus|YRFIR2742-23
Paramisgurnus dabryanus|YRFIR326-23
Paramisgurnus dabryanus|YRFIR2099-23
Paramisgurnus dabryanus|YRFIR2743-23
Paramisgurnus dabryanus|YRFIR2744-23
Paramisgurnus dabryanus|YRFIR2745-23
Paramisgurnus dabryanus|YRFIR2746-23
Paramisgurnus dabryanus|YRFIR2747-23
Paramisgurnus dabryanus|YRFIR3159-23
Rhodeus notatus|YRFIR1323-23
Rhodeus sinensis|YRFIR1283-23
Rhodeus sinensis|YRFIR1324-23
Rhodeus ocellatus|YRFIR1321-23
Rhodeus ocellatus|YRFIR1322-23
Rhodeus ocellatus|YRFIR1340-23
Rhodeus ocellatus|YRFIR1341-23
Rhodeus ocellatus|YRFIR1244-23
Rhodeus ocellatus|YRFIR2834-23
Rhodeus ocellatus|YRFIR1287-23
Rhodeus ocellatus|YRFIR1348-23
Rhodeus ocellatus|YRFIR1349-23
Rhodeus ocellatus|YRFIR1245-23
Rhodeus ocellatus|YRFIR1350-23
Rhodeus ocellatus|YRFIR2835-23
Rhodeus ocellatus|YRFIR1285-23
Rhodeus ocellatus|YRFIR1279-23
Rhodeus ocellatus|YRFIR1282-23
Rhodeus ocellatus|YRFIR1293-23
Rhodeus ocellatus|YRFIR1297-23
Rhodeus ocellatus|YRFIR1299-23
Rhodeus ocellatus|YRFIR1326-23
Rhodeus ocellatus|YRFIR1325-23
Rhodeus ocellatus|YRFIR2918-23
Rhodeus ocellatus|YRFIR1272-23
Rhodeus ocellatus|YRFIR1280-23
Rhodeus ocellatus|YRFIR1294-23
Rhodeus ocellatus|YRFIR1251-23
Rhodeus ocellatus|YRFIR1250-23
Rhodeus ocellatus|YRFIR1252-23
Rhodeus ocellatus|YRFIR1253-23
Rhodeus ocellatus|YRFIR1254-23
Rhodeus ocellatus|YRFIR1255-23
Rhodeus ocellatus|YRFIR1337-23
Rhodeus ocellatus|YRFIR1305-23
Rhodeus ocellatus|YRFIR1304-23
Rhodeus ocellatus|YRFIR1301-23
Rhodeus ocellatus|YRFIR1267-23
Rhodeus ocellatus|YRFIR1265-23
Rhodeus ocellatus|YRFIR2117-23
Rhodeus ocellatus|YRFIR1344-23
Rhodeus ocellatus|YRFIR1343-23
Rhodeus ocellatus|YRFIR1342-23
Rhodeus ocellatus|YRFIR1338-23
Rhodeus ocellatus|YRFIR1249-23
Rhodeus ocellatus|YRFIR1248-23
Rhodeus ocellatus|YRFIR1247-23
Rhodeus ocellatus|YRFIR1246-23
Rhodeus ocellatus|YRFIR1266-23
Rhodeus ocellatus|YRFIR1275-23
Rhodeus ocellatus|YRFIR1276-23
Rhodeus ocellatus|YRFIR1277-23
Rhodeus ocellatus|YRFIR1278-23
Rhodeus ocellatus|YRFIR1281-23
Rhodeus ocellatus|YRFIR1284-23
Rhodeus ocellatus|YRFIR1286-23
Rhodeus ocellatus|YRFIR1288-23
Rhodeus ocellatus|YRFIR1289-23
Rhodeus ocellatus|YRFIR1290-23
Rhodeus ocellatus|YRFIR1291-23
Rhodeus ocellatus|YRFIR1292-23
Rhodeus ocellatus|YRFIR1295-23
Rhodeus ocellatus|YRFIR1296-23
Rhodeus ocellatus|YRFIR1300-23
Rhodeus ocellatus|YRFIR1302-23
Rhodeus ocellatus|YRFIR1303-23
Rhodeus ocellatus|YRFIR2880-23
Rhodeus ocellatus|YRFIR1345-23
Rhodeus ocellatus|YRFIR1346-23
Rhodeus ocellatus|YRFIR1347-23
Rhodeus ocellatus|YRFIR3011-23
Rhodeus ocellatus|YRFIR2114-23
Rhodeus ocellatus|YRFIR2115-23
Rhodeus ocellatus|YRFIR2116-23
Rhodeus ocellatus|YRFIR1330-23
Rhodeus ocellatus|YRFIR1329-23
Rhodeus ocellatus|YRFIR1328-23
Rhodeus ocellatus|YRFIR1327-23
Rhodeus ocellatus|YRFIR1331-23
Rhodeus ocellatus|YRFIR1332-23
Rhodeus ocellatus|YRFIR1333-23
Rhodeus ocellatus|YRFIR1339-23
Rhodeus ocellatus|YRFIR3099-23
Rhodeus ocellatus|YRFIR3100-23
Acheilognathus macropterus|YRFIR1232-23
Acheilognathus macropterus|YRFIR1235-23
Acheilognathus macropterus|YRFIR1236-23
Acheilognathus macropterus|YRFIR1237-23
Acheilognathus macropterus|YRFIR1238-23
Acheilognathus macropterus|YRFIR1239-23

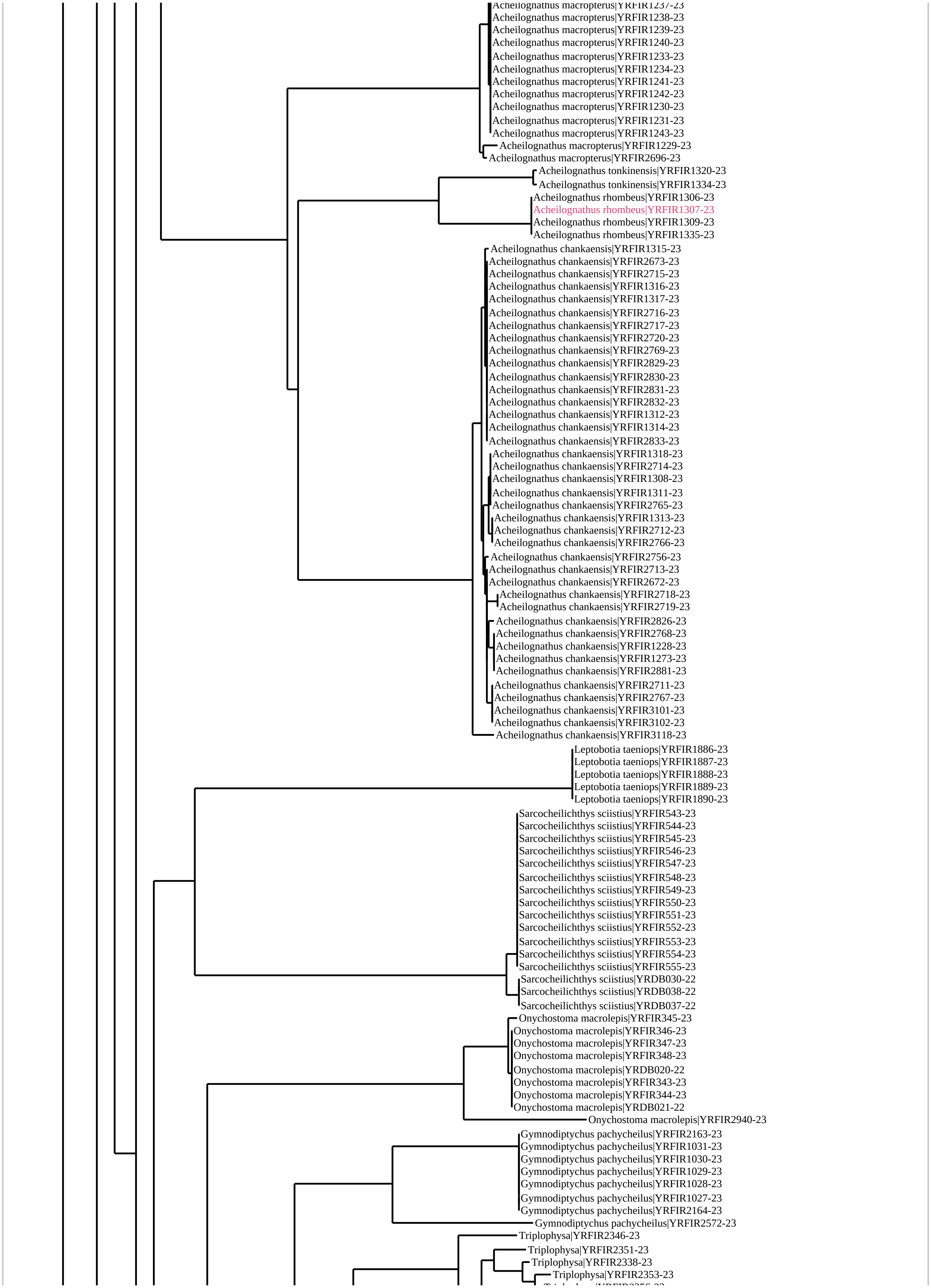
Acheilognathus macropterus|YRFIR1237-23
Acheilognathus macropterus|YRFIR1238-23
Acheilognathus macropterus|YRFIR1239-23
Acheilognathus macropterus|YRFIR1240-23
Acheilognathus macropterus|YRFIR1233-23
Acheilognathus macropterus|YRFIR1234-23
Acheilognathus macropterus|YRFIR1241-23
Acheilognathus macropterus|YRFIR1242-23
Acheilognathus macropterus|YRFIR1230-23
Acheilognathus macropterus|YRFIR1231-23
Acheilognathus macropterus|YRFIR1243-23
Acheilognathus macropterus|YRFIR1229-23
Acheilognathus macropterus|YRFIR2696-23
Acheilognathus tonkinensis|YRFIR1320-23
Acheilognathus tonkinensis|YRFIR1334-23
Acheilognathus rhombeus|YRFIR1306-23
Acheilognathus rhombeus|YRFIR1307-23
Acheilognathus rhombeus|YRFIR1309-23
Acheilognathus rhombeus|YRFIR1335-23
Acheilognathus chankaensis|YRFIR1315-23
Acheilognathus chankaensis|YRFIR2673-23
Acheilognathus chankaensis|YRFIR2715-23
Acheilognathus chankaensis|YRFIR1316-23
Acheilognathus chankaensis|YRFIR1317-23
Acheilognathus chankaensis|YRFIR2716-23
Acheilognathus chankaensis|YRFIR2717-23
Acheilognathus chankaensis|YRFIR2720-23
Acheilognathus chankaensis|YRFIR2769-23
Acheilognathus chankaensis|YRFIR2829-23
Acheilognathus chankaensis|YRFIR2830-23
Acheilognathus chankaensis|YRFIR2831-23
Acheilognathus chankaensis|YRFIR2832-23
Acheilognathus chankaensis|YRFIR1312-23
Acheilognathus chankaensis|YRFIR1314-23
Acheilognathus chankaensis|YRFIR2833-23
Acheilognathus chankaensis|YRFIR1318-23
Acheilognathus chankaensis|YRFIR2714-23
Acheilognathus chankaensis|YRFIR1308-23
Acheilognathus chankaensis|YRFIR1311-23
Acheilognathus chankaensis|YRFIR2765-23
Acheilognathus chankaensis|YRFIR1313-23
Acheilognathus chankaensis|YRFIR2712-23
Acheilognathus chankaensis|YRFIR2766-23
Acheilognathus chankaensis|YRFIR2756-23
Acheilognathus chankaensis|YRFIR2713-23
Acheilognathus chankaensis|YRFIR2672-23
Acheilognathus chankaensis|YRFIR2718-23
Acheilognathus chankaensis|YRFIR2719-23
Acheilognathus chankaensis|YRFIR2826-23
Acheilognathus chankaensis|YRFIR2768-23
Acheilognathus chankaensis|YRFIR1228-23
Acheilognathus chankaensis|YRFIR1273-23
Acheilognathus chankaensis|YRFIR2881-23
Acheilognathus chankaensis|YRFIR2711-23
Acheilognathus chankaensis|YRFIR2767-23
Acheilognathus chankaensis|YRFIR3101-23
Acheilognathus chankaensis|YRFIR3102-23
Acheilognathus chankaensis|YRFIR3118-23
Leptobotia taeniops|YRFIR1886-23
Leptobotia taeniops|YRFIR1887-23
Leptobotia taeniops|YRFIR1888-23
Leptobotia taeniops|YRFIR1889-23
Leptobotia taeniops|YRFIR1890-23
Sarcocheilichthys sciistius|YRFIR543-23
Sarcocheilichthys sciistius|YRFIR544-23
Sarcocheilichthys sciistius|YRFIR545-23
Sarcocheilichthys sciistius|YRFIR546-23
Sarcocheilichthys sciistius|YRFIR547-23
Sarcocheilichthys sciistius|YRFIR548-23
Sarcocheilichthys sciistius|YRFIR549-23
Sarcocheilichthys sciistius|YRFIR550-23
Sarcocheilichthys sciistius|YRFIR551-23
Sarcocheilichthys sciistius|YRFIR552-23
Sarcocheilichthys sciistius|YRFIR553-23
Sarcocheilichthys sciistius|YRFIR554-23
Sarcocheilichthys sciistius|YRFIR555-23
Sarcocheilichthys sciistius|YRDB030-22
Sarcocheilichthys sciistius|YRDB038-22
Sarcocheilichthys sciistius|YRDB037-22
Onychostoma macrolepis|YRFIR345-23
Onychostoma macrolepis|YRFIR346-23
Onychostoma macrolepis|YRFIR347-23
Onychostoma macrolepis|YRFIR348-23
Onychostoma macrolepis|YRDB020-22
Onychostoma macrolepis|YRFIR343-23
Onychostoma macrolepis|YRFIR344-23
Onychostoma macrolepis|YRDB021-22
Onychostoma macrolepis|YRFIR2940-23
Gymnodiptychus pachycheilus|YRFIR2163-23
Gymnodiptychus pachycheilus|YRFIR1031-23
Gymnodiptychus pachycheilus|YRFIR1030-23
Gymnodiptychus pachycheilus|YRFIR1029-23
Gymnodiptychus pachycheilus|YRFIR1028-23
Gymnodiptychus pachycheilus|YRFIR1027-23
Gymnodiptychus pachycheilus|YRFIR2164-23
Gymnodiptychus pachycheilus|YRFIR2572-23
Triplophysa|YRFIR2346-23
Triplophysa|YRFIR2351-23
Triplophysa|YRFIR2338-23
Triplophysa|YRFIR2353-23

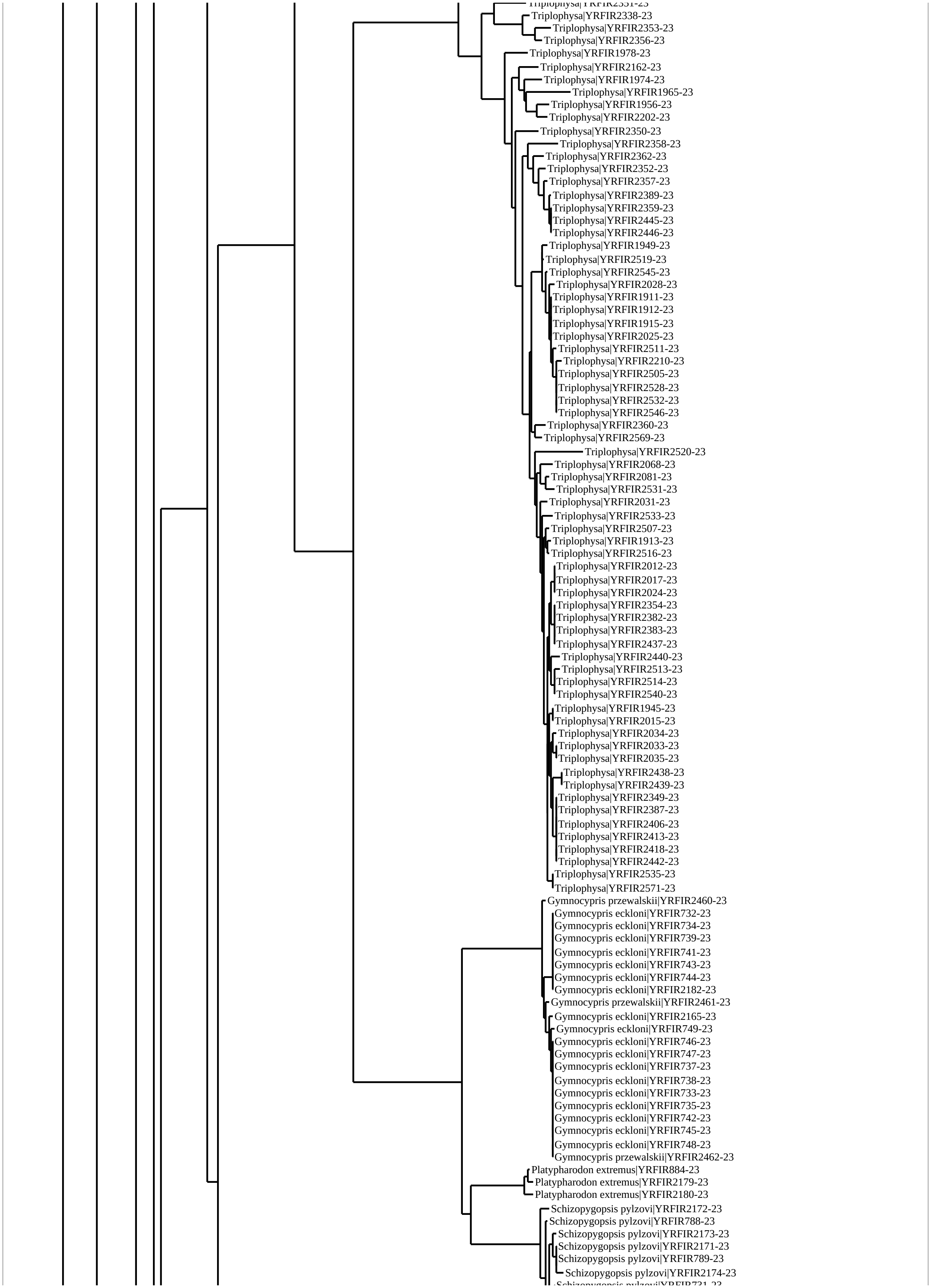
Triplophysa|YRFIR2338-23
Triplophysa|YRFIR2353-23
Triplophysa|YRFIR2356-23
Triplophysa|YRFIR1978-23
Triplophysa|YRFIR2162-23
Triplophysa|YRFIR1974-23
Triplophysa|YRFIR1965-23
Triplophysa|YRFIR1956-23
Triplophysa|YRFIR2202-23
Triplophysa|YRFIR2350-23
Triplophysa|YRFIR2358-23
Triplophysa|YRFIR2362-23
Triplophysa|YRFIR2352-23
Triplophysa|YRFIR2357-23
Triplophysa|YRFIR2389-23
Triplophysa|YRFIR2359-23
Triplophysa|YRFIR2445-23
Triplophysa|YRFIR2446-23
Triplophysa|YRFIR1949-23
Triplophysa|YRFIR2519-23
Triplophysa|YRFIR2545-23
Triplophysa|YRFIR2028-23
Triplophysa|YRFIR1911-23
Triplophysa|YRFIR1912-23
Triplophysa|YRFIR1915-23
Triplophysa|YRFIR2025-23
Triplophysa|YRFIR2511-23
Triplophysa|YRFIR2210-23
Triplophysa|YRFIR2505-23
Triplophysa|YRFIR2528-23
Triplophysa|YRFIR2532-23
Triplophysa|YRFIR2546-23
Triplophysa|YRFIR2360-23
Triplophysa|YRFIR2569-23
Triplophysa|YRFIR2520-23
Triplophysa|YRFIR2068-23
Triplophysa|YRFIR2081-23
Triplophysa|YRFIR2531-23
Triplophysa|YRFIR2031-23
Triplophysa|YRFIR2533-23
Triplophysa|YRFIR2507-23
Triplophysa|YRFIR1913-23
Triplophysa|YRFIR2516-23
Triplophysa|YRFIR2012-23
Triplophysa|YRFIR2017-23
Triplophysa|YRFIR2024-23
Triplophysa|YRFIR2354-23
Triplophysa|YRFIR2382-23
Triplophysa|YRFIR2383-23
Triplophysa|YRFIR2437-23
Triplophysa|YRFIR2440-23
Triplophysa|YRFIR2513-23
Triplophysa|YRFIR2514-23
Triplophysa|YRFIR2540-23
Triplophysa|YRFIR1945-23
Triplophysa|YRFIR2015-23
Triplophysa|YRFIR2034-23
Triplophysa|YRFIR2033-23
Triplophysa|YRFIR2035-23
Triplophysa|YRFIR2438-23
Triplophysa|YRFIR2439-23
Triplophysa|YRFIR2349-23
Triplophysa|YRFIR2387-23
Triplophysa|YRFIR2406-23
Triplophysa|YRFIR2413-23
Triplophysa|YRFIR2418-23
Triplophysa|YRFIR2442-23
Triplophysa|YRFIR2535-23
Triplophysa|YRFIR2571-23
Gymnocypris przewalskii|YRFIR2460-23
Gymnocypris eckloni|YRFIR732-23
Gymnocypris eckloni|YRFIR734-23
Gymnocypris eckloni|YRFIR739-23
Gymnocypris eckloni|YRFIR741-23
Gymnocypris eckloni|YRFIR743-23
Gymnocypris eckloni|YRFIR744-23
Gymnocypris eckloni|YRFIR2182-23
Gymnocypris przewalskii|YRFIR2461-23
Gymnocypris eckloni|YRFIR2165-23
Gymnocypris eckloni|YRFIR749-23
Gymnocypris eckloni|YRFIR746-23
Gymnocypris eckloni|YRFIR747-23
Gymnocypris eckloni|YRFIR737-23
Gymnocypris eckloni|YRFIR738-23
Gymnocypris eckloni|YRFIR733-23
Gymnocypris eckloni|YRFIR735-23
Gymnocypris eckloni|YRFIR742-23
Gymnocypris eckloni|YRFIR745-23
Gymnocypris eckloni|YRFIR748-23
Gymnocypris przewalskii|YRFIR2462-23
Platypharodon extremus|YRFIR884-23
Platypharodon extremus|YRFIR2179-23
Platypharodon extremus|YRFIR2180-23
Schizopygopsis pylzovi|YRFIR2172-23
Schizopygopsis pylzovi|YRFIR788-23
Schizopygopsis pylzovi|YRFIR2173-23
Schizopygopsis pylzovi|YRFIR2171-23
Schizopygopsis pylzovi|YRFIR789-23
Schizopygopsis pylzovi|YRFIR2174-23

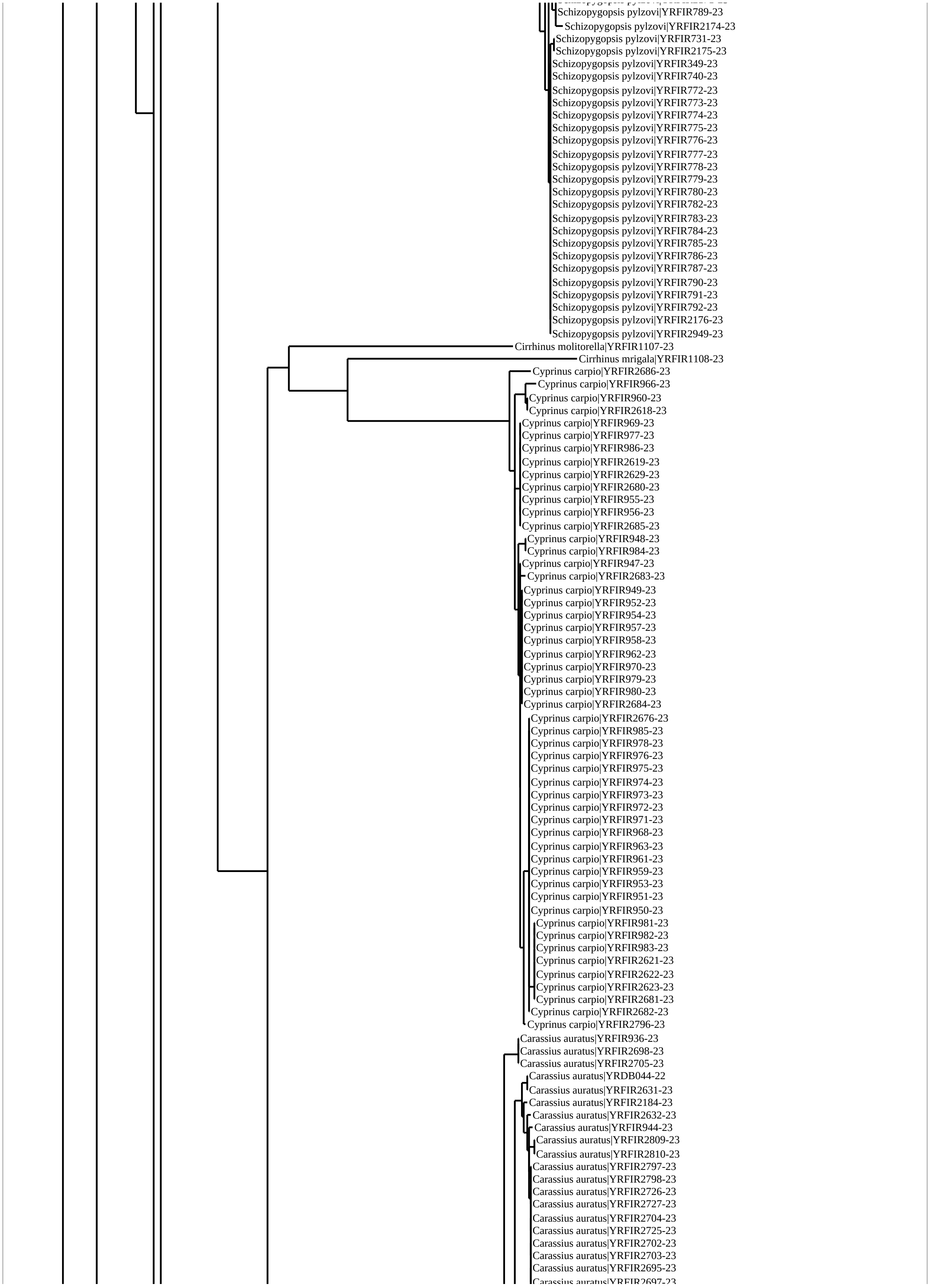
Schizopygopsis pylzovi|YRFIR789-23
Schizopygopsis pylzovi|YRFIR2174-23
Schizopygopsis pylzovi|YRFIR731-23
Schizopygopsis pylzovi|YRFIR2175-23
Schizopygopsis pylzovi|YRFIR349-23
Schizopygopsis pylzovi|YRFIR740-23
Schizopygopsis pylzovi|YRFIR772-23
Schizopygopsis pylzovi|YRFIR773-23
Schizopygopsis pylzovi|YRFIR774-23
Schizopygopsis pylzovi|YRFIR775-23
Schizopygopsis pylzovi|YRFIR776-23
Schizopygopsis pylzovi|YRFIR777-23
Schizopygopsis pylzovi|YRFIR778-23
Schizopygopsis pylzovi|YRFIR779-23
Schizopygopsis pylzovi|YRFIR780-23
Schizopygopsis pylzovi|YRFIR782-23
Schizopygopsis pylzovi|YRFIR783-23
Schizopygopsis pylzovi|YRFIR784-23
Schizopygopsis pylzovi|YRFIR785-23
Schizopygopsis pylzovi|YRFIR786-23
Schizopygopsis pylzovi|YRFIR787-23
Schizopygopsis pylzovi|YRFIR790-23
Schizopygopsis pylzovi|YRFIR791-23
Schizopygopsis pylzovi|YRFIR792-23
Schizopygopsis pylzovi|YRFIR2176-23
Schizopygopsis pylzovi|YRFIR2949-23
Cirrhinus molitorella|YRFIR1107-23
Cirrhinus mrigala|YRFIR1108-23
Cyprinus carpio|YRFIR2686-23
Cyprinus carpio|YRFIR966-23
Cyprinus carpio|YRFIR960-23
Cyprinus carpio|YRFIR2618-23
Cyprinus carpio|YRFIR969-23
Cyprinus carpio|YRFIR977-23
Cyprinus carpio|YRFIR986-23
Cyprinus carpio|YRFIR2619-23
Cyprinus carpio|YRFIR2629-23
Cyprinus carpio|YRFIR2680-23
Cyprinus carpio|YRFIR955-23
Cyprinus carpio|YRFIR956-23
Cyprinus carpio|YRFIR2685-23
Cyprinus carpio|YRFIR948-23
Cyprinus carpio|YRFIR984-23
Cyprinus carpio|YRFIR947-23
Cyprinus carpio|YRFIR2683-23
Cyprinus carpio|YRFIR949-23
Cyprinus carpio|YRFIR952-23
Cyprinus carpio|YRFIR954-23
Cyprinus carpio|YRFIR957-23
Cyprinus carpio|YRFIR958-23
Cyprinus carpio|YRFIR962-23
Cyprinus carpio|YRFIR970-23
Cyprinus carpio|YRFIR979-23
Cyprinus carpio|YRFIR980-23
Cyprinus carpio|YRFIR2684-23
Cyprinus carpio|YRFIR2676-23
Cyprinus carpio|YRFIR985-23
Cyprinus carpio|YRFIR978-23
Cyprinus carpio|YRFIR976-23
Cyprinus carpio|YRFIR975-23
Cyprinus carpio|YRFIR974-23
Cyprinus carpio|YRFIR973-23
Cyprinus carpio|YRFIR972-23
Cyprinus carpio|YRFIR971-23
Cyprinus carpio|YRFIR968-23
Cyprinus carpio|YRFIR963-23
Cyprinus carpio|YRFIR961-23
Cyprinus carpio|YRFIR959-23
Cyprinus carpio|YRFIR953-23
Cyprinus carpio|YRFIR951-23
Cyprinus carpio|YRFIR950-23
Cyprinus carpio|YRFIR981-23
Cyprinus carpio|YRFIR982-23
Cyprinus carpio|YRFIR983-23
Cyprinus carpio|YRFIR2621-23
Cyprinus carpio|YRFIR2622-23
Cyprinus carpio|YRFIR2623-23
Cyprinus carpio|YRFIR2681-23
Cyprinus carpio|YRFIR2682-23
Cyprinus carpio|YRFIR2796-23
Carassius auratus|YRFIR936-23
Carassius auratus|YRFIR2698-23
Carassius auratus|YRFIR2705-23
Carassius auratus|YRDB044-22
Carassius auratus|YRFIR2631-23
Carassius auratus|YRFIR2184-23
Carassius auratus|YRFIR2632-23
Carassius auratus|YRFIR944-23
Carassius auratus|YRFIR2809-23
Carassius auratus|YRFIR2810-23
Carassius auratus|YRFIR2797-23
Carassius auratus|YRFIR2798-23
Carassius auratus|YRFIR2726-23
Carassius auratus|YRFIR2727-23
Carassius auratus|YRFIR2704-23
Carassius auratus|YRFIR2725-23
Carassius auratus|YRFIR2702-23
Carassius auratus|YRFIR2703-23
Carassius auratus|YRFIR2695-23
Carassius auratus|YRFIR2697-23

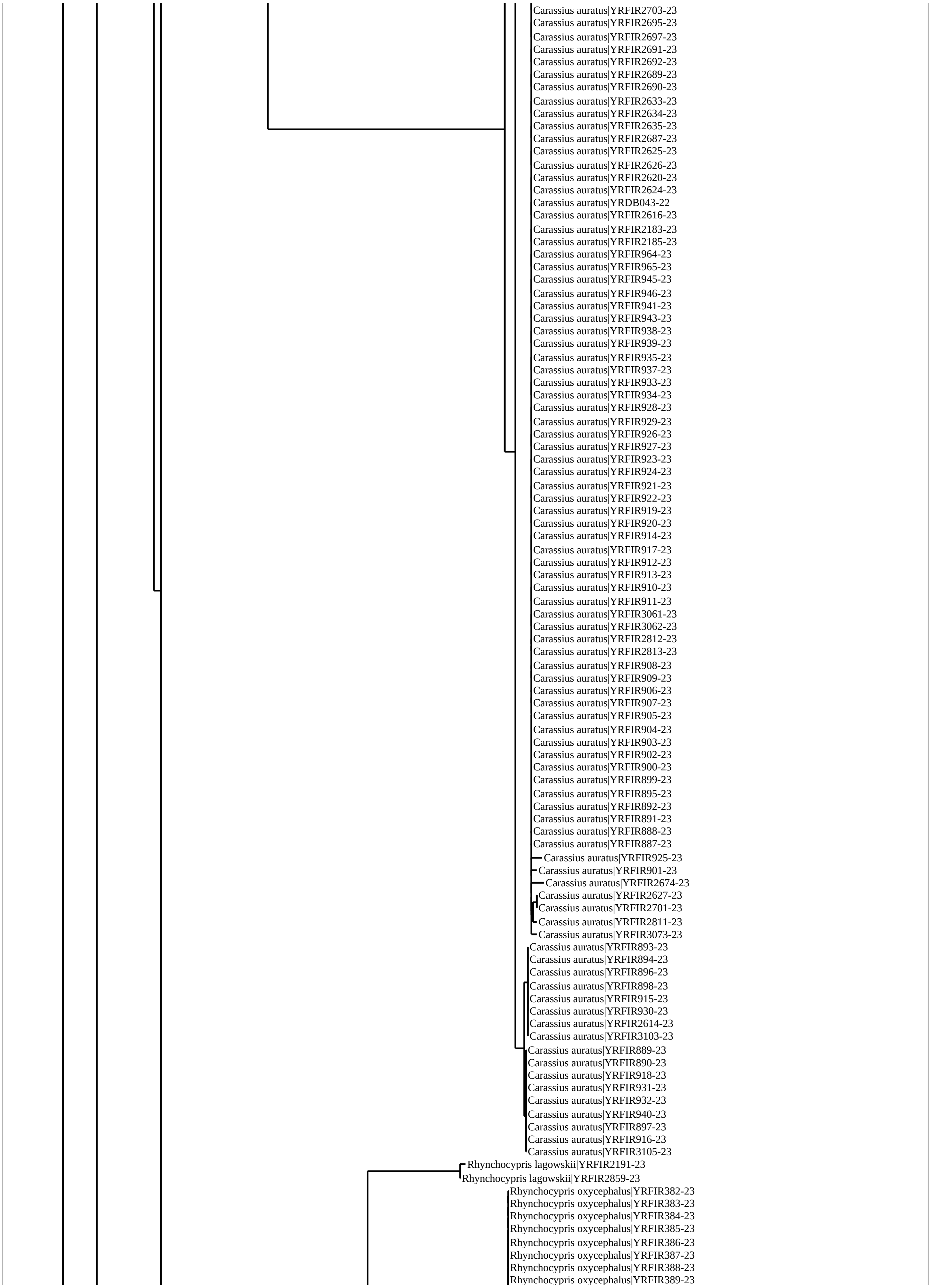

Carassius auratus|YRFIR2703-23
Carassius auratus|YRFIR2695-23
Carassius auratus|YRFIR2697-23
Carassius auratus|YRFIR2691-23
Carassius auratus|YRFIR2692-23
Carassius auratus|YRFIR2689-23
Carassius auratus|YRFIR2690-23
Carassius auratus|YRFIR2633-23
Carassius auratus|YRFIR2634-23
Carassius auratus|YRFIR2635-23
Carassius auratus|YRFIR2687-23
Carassius auratus|YRFIR2625-23
Carassius auratus|YRFIR2626-23
Carassius auratus|YRFIR2620-23
Carassius auratus|YRFIR2624-23
Carassius auratus|YRDB043-22
Carassius auratus|YRFIR2616-23
Carassius auratus|YRFIR2183-23
Carassius auratus|YRFIR2185-23
Carassius auratus|YRFIR964-23
Carassius auratus|YRFIR965-23
Carassius auratus|YRFIR945-23
Carassius auratus|YRFIR946-23
Carassius auratus|YRFIR941-23
Carassius auratus|YRFIR943-23
Carassius auratus|YRFIR938-23
Carassius auratus|YRFIR939-23
Carassius auratus|YRFIR935-23
Carassius auratus|YRFIR937-23
Carassius auratus|YRFIR933-23
Carassius auratus|YRFIR934-23
Carassius auratus|YRFIR928-23
Carassius auratus|YRFIR929-23
Carassius auratus|YRFIR926-23
Carassius auratus|YRFIR927-23
Carassius auratus|YRFIR923-23
Carassius auratus|YRFIR924-23
Carassius auratus|YRFIR921-23
Carassius auratus|YRFIR922-23
Carassius auratus|YRFIR919-23
Carassius auratus|YRFIR920-23
Carassius auratus|YRFIR914-23
Carassius auratus|YRFIR917-23
Carassius auratus|YRFIR912-23
Carassius auratus|YRFIR913-23
Carassius auratus|YRFIR910-23
Carassius auratus|YRFIR911-23
Carassius auratus|YRFIR3061-23
Carassius auratus|YRFIR3062-23
Carassius auratus|YRFIR2812-23
Carassius auratus|YRFIR2813-23
Carassius auratus|YRFIR908-23
Carassius auratus|YRFIR909-23
Carassius auratus|YRFIR906-23
Carassius auratus|YRFIR907-23
Carassius auratus|YRFIR905-23
Carassius auratus|YRFIR904-23
Carassius auratus|YRFIR903-23
Carassius auratus|YRFIR902-23
Carassius auratus|YRFIR900-23
Carassius auratus|YRFIR899-23
Carassius auratus|YRFIR895-23
Carassius auratus|YRFIR892-23
Carassius auratus|YRFIR891-23
Carassius auratus|YRFIR888-23
Carassius auratus|YRFIR887-23
Carassius auratus|YRFIR925-23
Carassius auratus|YRFIR901-23
Carassius auratus|YRFIR2674-23
Carassius auratus|YRFIR2627-23
Carassius auratus|YRFIR2701-23
Carassius auratus|YRFIR2811-23
Carassius auratus|YRFIR3073-23
Carassius auratus|YRFIR893-23
Carassius auratus|YRFIR894-23
Carassius auratus|YRFIR896-23
Carassius auratus|YRFIR898-23
Carassius auratus|YRFIR915-23
Carassius auratus|YRFIR930-23
Carassius auratus|YRFIR2614-23
Carassius auratus|YRFIR3103-23
Carassius auratus|YRFIR889-23
Carassius auratus|YRFIR890-23
Carassius auratus|YRFIR918-23
Carassius auratus|YRFIR931-23
Carassius auratus|YRFIR932-23
Carassius auratus|YRFIR940-23
Carassius auratus|YRFIR897-23
Carassius auratus|YRFIR916-23
Carassius auratus|YRFIR3105-23
Rhynchocypris lagowskii|YRFIR2191-23
Rhynchocypris lagowskii|YRFIR2859-23
Rhynchocypris oxycephalus|YRFIR382-23
Rhynchocypris oxycephalus|YRFIR383-23
Rhynchocypris oxycephalus|YRFIR384-23
Rhynchocypris oxycephalus|YRFIR385-23
Rhynchocypris oxycephalus|YRFIR386-23
Rhynchocypris oxycephalus|YRFIR387-23
Rhynchocypris oxycephalus|YRFIR388-23
Rhynchocypris oxycephalus|YRFIR389-23

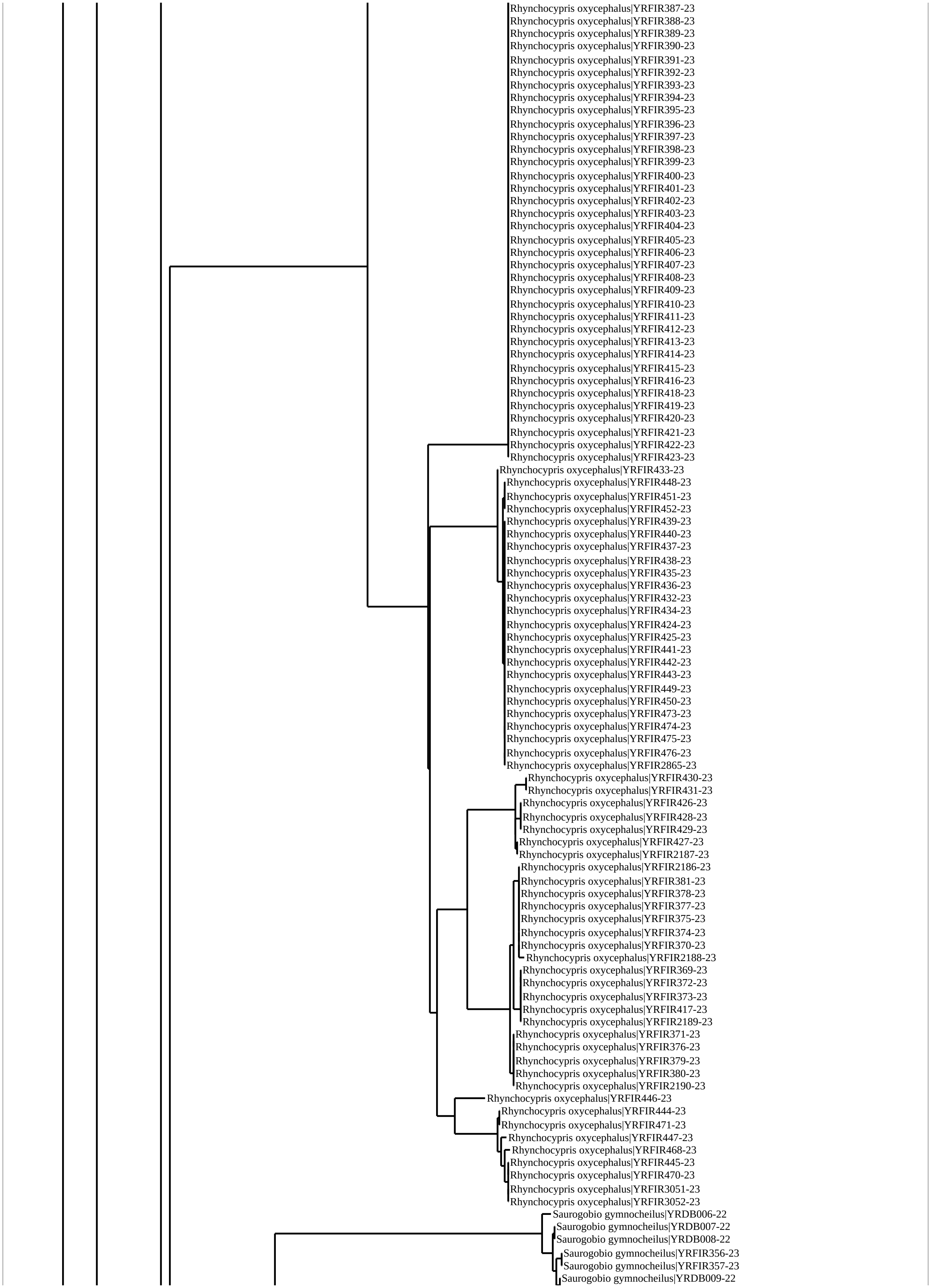
Rhynchocypris oxycephalus|YRFIR387-23
Rhynchocypris oxycephalus|YRFIR388-23
Rhynchocypris oxycephalus|YRFIR389-23
Rhynchocypris oxycephalus|YRFIR390-23
Rhynchocypris oxycephalus|YRFIR391-23
Rhynchocypris oxycephalus|YRFIR392-23
Rhynchocypris oxycephalus|YRFIR393-23
Rhynchocypris oxycephalus|YRFIR394-23
Rhynchocypris oxycephalus|YRFIR395-23
Rhynchocypris oxycephalus|YRFIR396-23
Rhynchocypris oxycephalus|YRFIR397-23
Rhynchocypris oxycephalus|YRFIR398-23
Rhynchocypris oxycephalus|YRFIR399-23
Rhynchocypris oxycephalus|YRFIR400-23
Rhynchocypris oxycephalus|YRFIR401-23
Rhynchocypris oxycephalus|YRFIR402-23
Rhynchocypris oxycephalus|YRFIR403-23
Rhynchocypris oxycephalus|YRFIR404-23
Rhynchocypris oxycephalus|YRFIR405-23
Rhynchocypris oxycephalus|YRFIR406-23
Rhynchocypris oxycephalus|YRFIR407-23
Rhynchocypris oxycephalus|YRFIR408-23
Rhynchocypris oxycephalus|YRFIR409-23
Rhynchocypris oxycephalus|YRFIR410-23
Rhynchocypris oxycephalus|YRFIR411-23
Rhynchocypris oxycephalus|YRFIR412-23
Rhynchocypris oxycephalus|YRFIR413-23
Rhynchocypris oxycephalus|YRFIR414-23
Rhynchocypris oxycephalus|YRFIR415-23
Rhynchocypris oxycephalus|YRFIR416-23
Rhynchocypris oxycephalus|YRFIR418-23
Rhynchocypris oxycephalus|YRFIR419-23
Rhynchocypris oxycephalus|YRFIR420-23
Rhynchocypris oxycephalus|YRFIR421-23
Rhynchocypris oxycephalus|YRFIR422-23
Rhynchocypris oxycephalus|YRFIR423-23
Rhynchocypris oxycephalus|YRFIR433-23
Rhynchocypris oxycephalus|YRFIR448-23
Rhynchocypris oxycephalus|YRFIR451-23
Rhynchocypris oxycephalus|YRFIR452-23
Rhynchocypris oxycephalus|YRFIR439-23
Rhynchocypris oxycephalus|YRFIR440-23
Rhynchocypris oxycephalus|YRFIR437-23
Rhynchocypris oxycephalus|YRFIR438-23
Rhynchocypris oxycephalus|YRFIR435-23
Rhynchocypris oxycephalus|YRFIR436-23
Rhynchocypris oxycephalus|YRFIR432-23
Rhynchocypris oxycephalus|YRFIR434-23
Rhynchocypris oxycephalus|YRFIR424-23
Rhynchocypris oxycephalus|YRFIR425-23
Rhynchocypris oxycephalus|YRFIR441-23
Rhynchocypris oxycephalus|YRFIR442-23
Rhynchocypris oxycephalus|YRFIR443-23
Rhynchocypris oxycephalus|YRFIR449-23
Rhynchocypris oxycephalus|YRFIR450-23
Rhynchocypris oxycephalus|YRFIR473-23
Rhynchocypris oxycephalus|YRFIR474-23
Rhynchocypris oxycephalus|YRFIR475-23
Rhynchocypris oxycephalus|YRFIR476-23
Rhynchocypris oxycephalus|YRFIR2865-23
Rhynchocypris oxycephalus|YRFIR430-23
Rhynchocypris oxycephalus|YRFIR431-23
Rhynchocypris oxycephalus|YRFIR426-23
Rhynchocypris oxycephalus|YRFIR428-23
Rhynchocypris oxycephalus|YRFIR429-23
Rhynchocypris oxycephalus|YRFIR427-23
Rhynchocypris oxycephalus|YRFIR2187-23
Rhynchocypris oxycephalus|YRFIR2186-23
Rhynchocypris oxycephalus|YRFIR381-23
Rhynchocypris oxycephalus|YRFIR378-23
Rhynchocypris oxycephalus|YRFIR377-23
Rhynchocypris oxycephalus|YRFIR375-23
Rhynchocypris oxycephalus|YRFIR374-23
Rhynchocypris oxycephalus|YRFIR370-23
Rhynchocypris oxycephalus|YRFIR2188-23
Rhynchocypris oxycephalus|YRFIR369-23
Rhynchocypris oxycephalus|YRFIR372-23
Rhynchocypris oxycephalus|YRFIR373-23
Rhynchocypris oxycephalus|YRFIR417-23
Rhynchocypris oxycephalus|YRFIR2189-23
Rhynchocypris oxycephalus|YRFIR371-23
Rhynchocypris oxycephalus|YRFIR376-23
Rhynchocypris oxycephalus|YRFIR379-23
Rhynchocypris oxycephalus|YRFIR380-23
Rhynchocypris oxycephalus|YRFIR2190-23
Rhynchocypris oxycephalus|YRFIR446-23
Rhynchocypris oxycephalus|YRFIR444-23
Rhynchocypris oxycephalus|YRFIR471-23
Rhynchocypris oxycephalus|YRFIR447-23
Rhynchocypris oxycephalus|YRFIR468-23
Rhynchocypris oxycephalus|YRFIR445-23
Rhynchocypris oxycephalus|YRFIR470-23
Rhynchocypris oxycephalus|YRFIR3051-23
Rhynchocypris oxycephalus|YRFIR3052-23
Saurogobio gymnocheilus|YRDB006-22
Saurogobio gymnocheilus|YRDB007-22
Saurogobio gymnocheilus|YRDB008-22
Saurogobio gymnocheilus|YRFIR356-23
Saurogobio gymnocheilus|YRFIR357-23
Saurogobio gymnocheilus|YRDB009-22

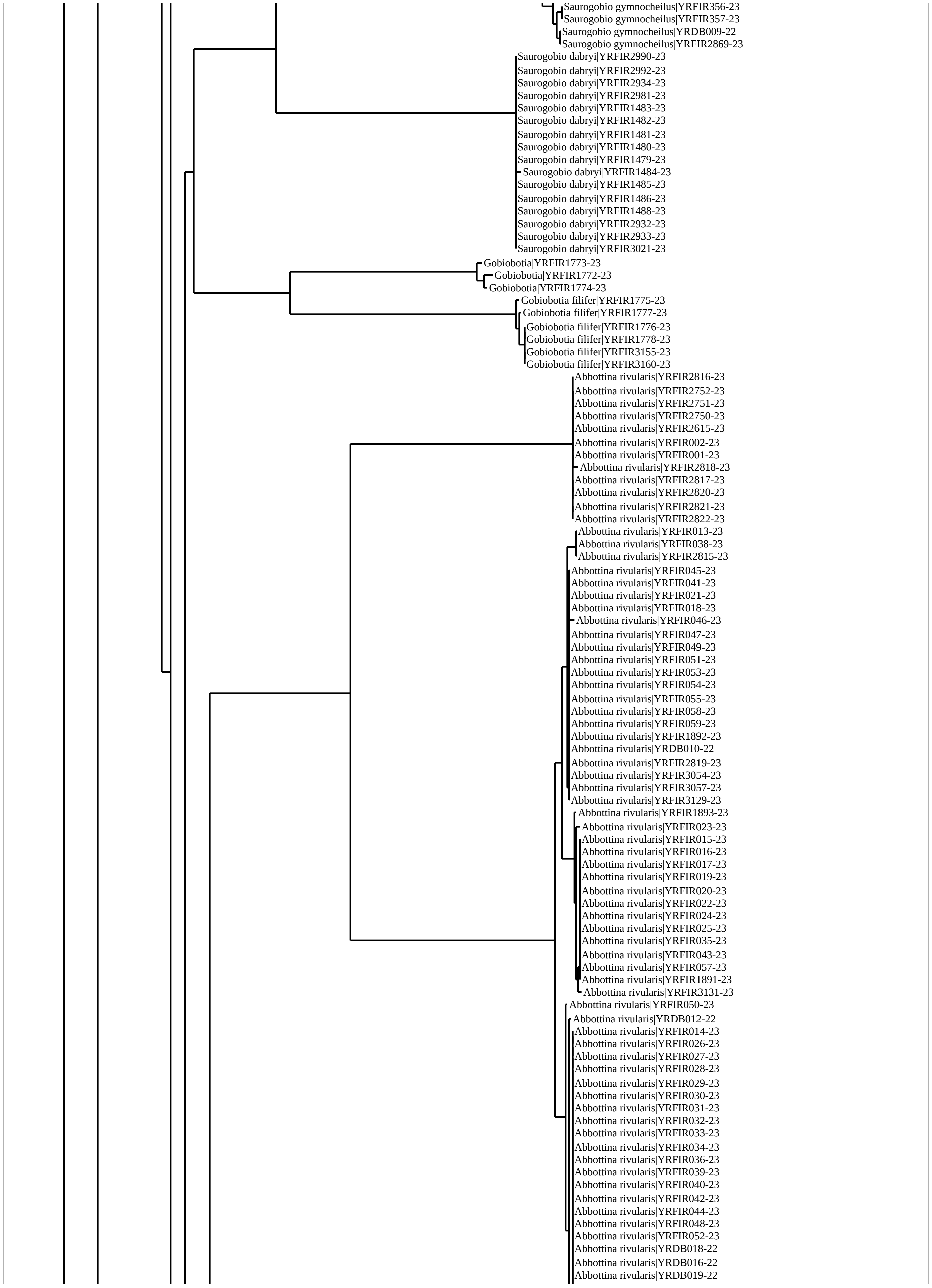
Saurogobio gymnocheilus|YRFIR356-23
Saurogobio gymnocheilus|YRFIR357-23
Saurogobio gymnocheilus|YRDB009-22
Saurogobio gymnocheilus|YRFIR2869-23
Saurogobio dabryi|YRFIR2990-23
Saurogobio dabryi|YRFIR2992-23
Saurogobio dabryi|YRFIR2934-23
Saurogobio dabryi|YRFIR2981-23
Saurogobio dabryi|YRFIR1483-23
Saurogobio dabryi|YRFIR1482-23
Saurogobio dabryi|YRFIR1481-23
Saurogobio dabryi|YRFIR1480-23
Saurogobio dabryi|YRFIR1479-23
Saurogobio dabryi|YRFIR1484-23
Saurogobio dabryi|YRFIR1485-23
Saurogobio dabryi|YRFIR1486-23
Saurogobio dabryi|YRFIR1488-23
Saurogobio dabryi|YRFIR2932-23
Saurogobio dabryi|YRFIR2933-23
Saurogobio dabryi|YRFIR3021-23
Gobiobotia|YRFIR1773-23
Gobiobotia|YRFIR1772-23
Gobiobotia|YRFIR1774-23
Gobiobotia filifer|YRFIR1775-23
Gobiobotia filifer|YRFIR1777-23
Gobiobotia filifer|YRFIR1776-23
Gobiobotia filifer|YRFIR1778-23
Gobiobotia filifer|YRFIR3155-23
Gobiobotia filifer|YRFIR3160-23
Abbottina rivularis|YRFIR2816-23
Abbottina rivularis|YRFIR2752-23
Abbottina rivularis|YRFIR2751-23
Abbottina rivularis|YRFIR2750-23
Abbottina rivularis|YRFIR2615-23
Abbottina rivularis|YRFIR002-23
Abbottina rivularis|YRFIR001-23
Abbottina rivularis|YRFIR2818-23
Abbottina rivularis|YRFIR2817-23
Abbottina rivularis|YRFIR2820-23
Abbottina rivularis|YRFIR2821-23
Abbottina rivularis|YRFIR2822-23
Abbottina rivularis|YRFIR013-23
Abbottina rivularis|YRFIR038-23
Abbottina rivularis|YRFIR2815-23
Abbottina rivularis|YRFIR045-23
Abbottina rivularis|YRFIR041-23
Abbottina rivularis|YRFIR021-23
Abbottina rivularis|YRFIR018-23
Abbottina rivularis|YRFIR046-23
Abbottina rivularis|YRFIR047-23
Abbottina rivularis|YRFIR049-23
Abbottina rivularis|YRFIR051-23
Abbottina rivularis|YRFIR053-23
Abbottina rivularis|YRFIR054-23
Abbottina rivularis|YRFIR055-23
Abbottina rivularis|YRFIR058-23
Abbottina rivularis|YRFIR059-23
Abbottina rivularis|YRFIR1892-23
Abbottina rivularis|YRDB010-22
Abbottina rivularis|YRFIR2819-23
Abbottina rivularis|YRFIR3054-23
Abbottina rivularis|YRFIR3057-23
Abbottina rivularis|YRFIR3129-23
Abbottina rivularis|YRFIR1893-23
Abbottina rivularis|YRFIR023-23
Abbottina rivularis|YRFIR015-23
Abbottina rivularis|YRFIR016-23
Abbottina rivularis|YRFIR017-23
Abbottina rivularis|YRFIR019-23
Abbottina rivularis|YRFIR020-23
Abbottina rivularis|YRFIR022-23
Abbottina rivularis|YRFIR024-23
Abbottina rivularis|YRFIR025-23
Abbottina rivularis|YRFIR035-23
Abbottina rivularis|YRFIR043-23
Abbottina rivularis|YRFIR057-23
Abbottina rivularis|YRFIR1891-23
Abbottina rivularis|YRFIR3131-23
Abbottina rivularis|YRFIR050-23
Abbottina rivularis|YRDB012-22
Abbottina rivularis|YRFIR014-23
Abbottina rivularis|YRFIR026-23
Abbottina rivularis|YRFIR027-23
Abbottina rivularis|YRFIR028-23
Abbottina rivularis|YRFIR029-23
Abbottina rivularis|YRFIR030-23
Abbottina rivularis|YRFIR031-23
Abbottina rivularis|YRFIR032-23
Abbottina rivularis|YRFIR033-23
Abbottina rivularis|YRFIR034-23
Abbottina rivularis|YRFIR036-23
Abbottina rivularis|YRFIR039-23
Abbottina rivularis|YRFIR040-23
Abbottina rivularis|YRFIR042-23
Abbottina rivularis|YRFIR044-23
Abbottina rivularis|YRFIR048-23
Abbottina rivularis|YRFIR052-23
Abbottina rivularis|YRDB018-22
Abbottina rivularis|YRDB016-22
Abbottina rivularis|YRDB019-22

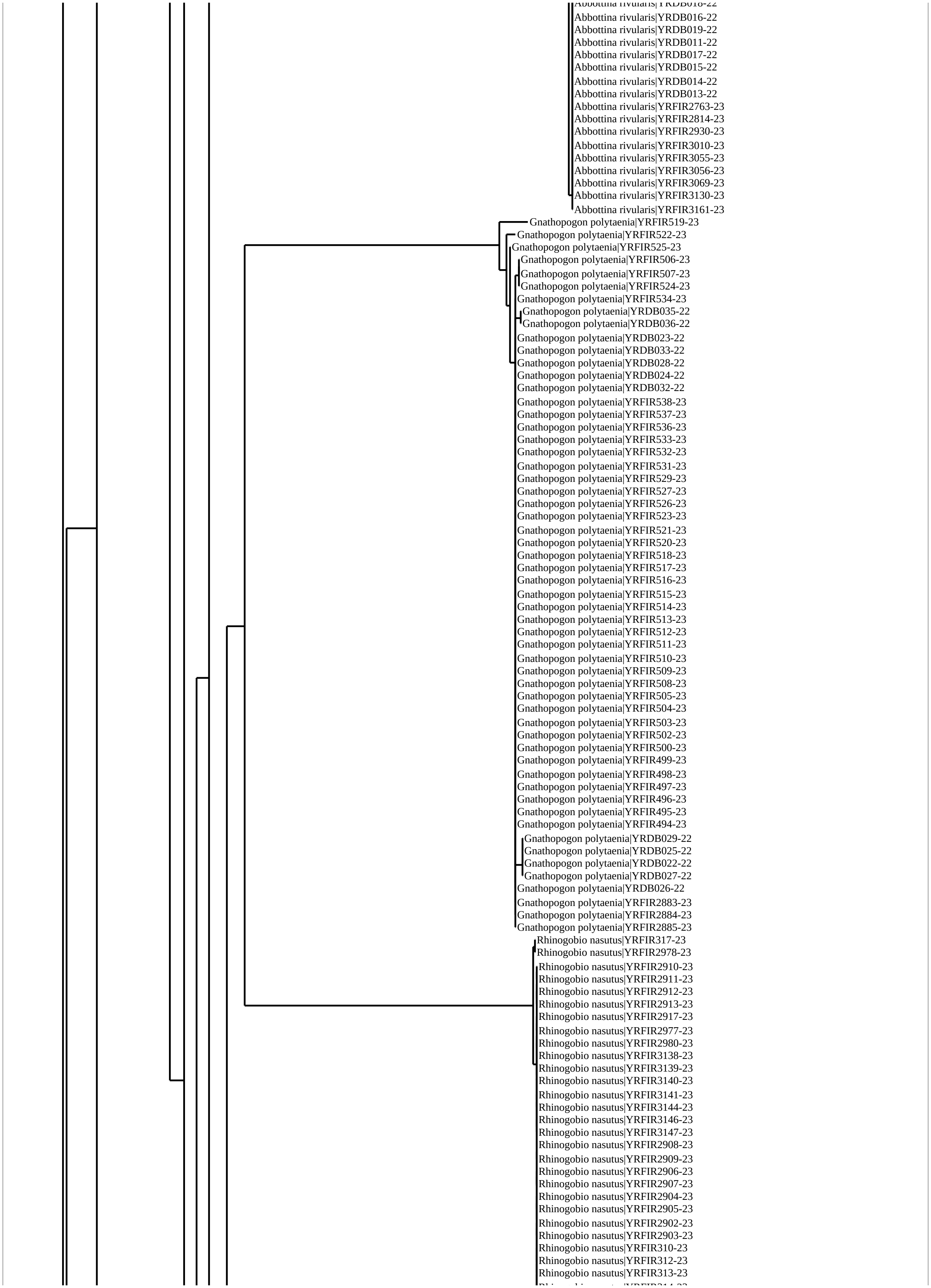
Abbottina rivularis|YRDB016-22
Abbottina rivularis|YRDB019-22
Abbottina rivularis|YRDB011-22
Abbottina rivularis|YRDB017-22
Abbottina rivularis|YRDB015-22
Abbottina rivularis|YRDB014-22
Abbottina rivularis|YRDB013-22
Abbottina rivularis|YRFIR2763-23
Abbottina rivularis|YRFIR2814-23
Abbottina rivularis|YRFIR2930-23
Abbottina rivularis|YRFIR3010-23
Abbottina rivularis|YRFIR3055-23
Abbottina rivularis|YRFIR3056-23
Abbottina rivularis|YRFIR3069-23
Abbottina rivularis|YRFIR3130-23
Abbottina rivularis|YRFIR3161-23
Gnathopogon polytaenia|YRFIR519-23
Gnathopogon polytaenia|YRFIR522-23
Gnathopogon polytaenia|YRFIR525-23
Gnathopogon polytaenia|YRFIR506-23
Gnathopogon polytaenia|YRFIR507-23
Gnathopogon polytaenia|YRFIR524-23
Gnathopogon polytaenia|YRFIR534-23
Gnathopogon polytaenia|YRDB035-22
Gnathopogon polytaenia|YRDB036-22
Gnathopogon polytaenia|YRDB023-22
Gnathopogon polytaenia|YRDB033-22
Gnathopogon polytaenia|YRDB028-22
Gnathopogon polytaenia|YRDB024-22
Gnathopogon polytaenia|YRDB032-22
Gnathopogon polytaenia|YRFIR538-23
Gnathopogon polytaenia|YRFIR537-23
Gnathopogon polytaenia|YRFIR536-23
Gnathopogon polytaenia|YRFIR533-23
Gnathopogon polytaenia|YRFIR532-23
Gnathopogon polytaenia|YRFIR531-23
Gnathopogon polytaenia|YRFIR529-23
Gnathopogon polytaenia|YRFIR527-23
Gnathopogon polytaenia|YRFIR526-23
Gnathopogon polytaenia|YRFIR523-23
Gnathopogon polytaenia|YRFIR521-23
Gnathopogon polytaenia|YRFIR520-23
Gnathopogon polytaenia|YRFIR518-23
Gnathopogon polytaenia|YRFIR517-23
Gnathopogon polytaenia|YRFIR516-23
Gnathopogon polytaenia|YRFIR515-23
Gnathopogon polytaenia|YRFIR514-23
Gnathopogon polytaenia|YRFIR513-23
Gnathopogon polytaenia|YRFIR512-23
Gnathopogon polytaenia|YRFIR511-23
Gnathopogon polytaenia|YRFIR510-23
Gnathopogon polytaenia|YRFIR509-23
Gnathopogon polytaenia|YRFIR508-23
Gnathopogon polytaenia|YRFIR505-23
Gnathopogon polytaenia|YRFIR504-23
Gnathopogon polytaenia|YRFIR503-23
Gnathopogon polytaenia|YRFIR502-23
Gnathopogon polytaenia|YRFIR500-23
Gnathopogon polytaenia|YRFIR499-23
Gnathopogon polytaenia|YRFIR498-23
Gnathopogon polytaenia|YRFIR497-23
Gnathopogon polytaenia|YRFIR496-23
Gnathopogon polytaenia|YRFIR495-23
Gnathopogon polytaenia|YRFIR494-23
Gnathopogon polytaenia|YRDB029-22
Gnathopogon polytaenia|YRDB025-22
Gnathopogon polytaenia|YRDB022-22
Gnathopogon polytaenia|YRDB027-22
Gnathopogon polytaenia|YRDB026-22
Gnathopogon polytaenia|YRFIR2883-23
Gnathopogon polytaenia|YRFIR2884-23
Gnathopogon polytaenia|YRFIR2885-23
Rhinogobio nasutus|YRFIR317-23
Rhinogobio nasutus|YRFIR2978-23
Rhinogobio nasutus|YRFIR2910-23
Rhinogobio nasutus|YRFIR2911-23
Rhinogobio nasutus|YRFIR2912-23
Rhinogobio nasutus|YRFIR2913-23
Rhinogobio nasutus|YRFIR2917-23
Rhinogobio nasutus|YRFIR2977-23
Rhinogobio nasutus|YRFIR2980-23
Rhinogobio nasutus|YRFIR3138-23
Rhinogobio nasutus|YRFIR3139-23
Rhinogobio nasutus|YRFIR3140-23
Rhinogobio nasutus|YRFIR3141-23
Rhinogobio nasutus|YRFIR3144-23
Rhinogobio nasutus|YRFIR3146-23
Rhinogobio nasutus|YRFIR3147-23
Rhinogobio nasutus|YRFIR2908-23
Rhinogobio nasutus|YRFIR2909-23
Rhinogobio nasutus|YRFIR2906-23
Rhinogobio nasutus|YRFIR2907-23
Rhinogobio nasutus|YRFIR2904-23
Rhinogobio nasutus|YRFIR2905-23
Rhinogobio nasutus|YRFIR2902-23
Rhinogobio nasutus|YRFIR2903-23
Rhinogobio nasutus|YRFIR310-23
Rhinogobio nasutus|YRFIR312-23
Rhinogobio nasutus|YRFIR313-23

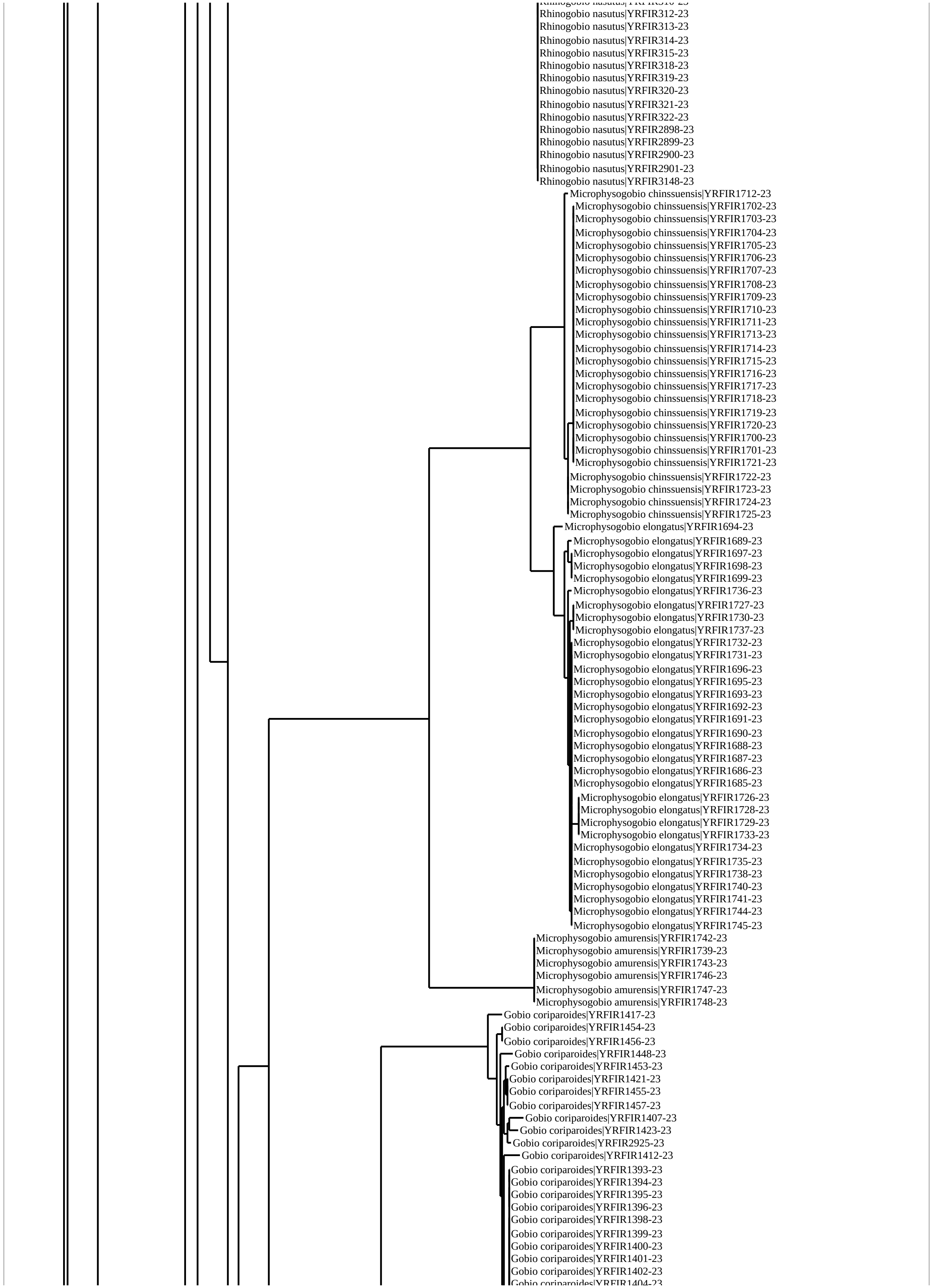
Rhinogobio nasutus|YRFIR312-23
Rhinogobio nasutus|YRFIR313-23
Rhinogobio nasutus|YRFIR314-23
Rhinogobio nasutus|YRFIR315-23
Rhinogobio nasutus|YRFIR318-23
Rhinogobio nasutus|YRFIR319-23
Rhinogobio nasutus|YRFIR320-23
Rhinogobio nasutus|YRFIR321-23
Rhinogobio nasutus|YRFIR322-23
Rhinogobio nasutus|YRFIR2898-23
Rhinogobio nasutus|YRFIR2899-23
Rhinogobio nasutus|YRFIR2900-23
Rhinogobio nasutus|YRFIR2901-23
Rhinogobio nasutus|YRFIR3148-23
Microphysogobio chinssuensis|YRFIR1712-23
Microphysogobio chinssuensis|YRFIR1702-23
Microphysogobio chinssuensis|YRFIR1703-23
Microphysogobio chinssuensis|YRFIR1704-23
Microphysogobio chinssuensis|YRFIR1705-23
Microphysogobio chinssuensis|YRFIR1706-23
Microphysogobio chinssuensis|YRFIR1707-23
Microphysogobio chinssuensis|YRFIR1708-23
Microphysogobio chinssuensis|YRFIR1709-23
Microphysogobio chinssuensis|YRFIR1710-23
Microphysogobio chinssuensis|YRFIR1711-23
Microphysogobio chinssuensis|YRFIR1713-23
Microphysogobio chinssuensis|YRFIR1714-23
Microphysogobio chinssuensis|YRFIR1715-23
Microphysogobio chinssuensis|YRFIR1716-23
Microphysogobio chinssuensis|YRFIR1717-23
Microphysogobio chinssuensis|YRFIR1718-23
Microphysogobio chinssuensis|YRFIR1719-23
Microphysogobio chinssuensis|YRFIR1720-23
Microphysogobio chinssuensis|YRFIR1700-23
Microphysogobio chinssuensis|YRFIR1701-23
Microphysogobio chinssuensis|YRFIR1721-23
Microphysogobio chinssuensis|YRFIR1722-23
Microphysogobio chinssuensis|YRFIR1723-23
Microphysogobio chinssuensis|YRFIR1724-23
Microphysogobio chinssuensis|YRFIR1725-23
Microphysogobio elongatus|YRFIR1694-23
Microphysogobio elongatus|YRFIR1689-23
Microphysogobio elongatus|YRFIR1697-23
Microphysogobio elongatus|YRFIR1698-23
Microphysogobio elongatus|YRFIR1699-23
Microphysogobio elongatus|YRFIR1736-23
Microphysogobio elongatus|YRFIR1727-23
Microphysogobio elongatus|YRFIR1730-23
Microphysogobio elongatus|YRFIR1737-23
Microphysogobio elongatus|YRFIR1732-23
Microphysogobio elongatus|YRFIR1731-23
Microphysogobio elongatus|YRFIR1696-23
Microphysogobio elongatus|YRFIR1695-23
Microphysogobio elongatus|YRFIR1693-23
Microphysogobio elongatus|YRFIR1692-23
Microphysogobio elongatus|YRFIR1691-23
Microphysogobio elongatus|YRFIR1690-23
Microphysogobio elongatus|YRFIR1688-23
Microphysogobio elongatus|YRFIR1687-23
Microphysogobio elongatus|YRFIR1686-23
Microphysogobio elongatus|YRFIR1685-23
Microphysogobio elongatus|YRFIR1726-23
Microphysogobio elongatus|YRFIR1728-23
Microphysogobio elongatus|YRFIR1729-23
Microphysogobio elongatus|YRFIR1733-23
Microphysogobio elongatus|YRFIR1734-23
Microphysogobio elongatus|YRFIR1735-23
Microphysogobio elongatus|YRFIR1738-23
Microphysogobio elongatus|YRFIR1740-23
Microphysogobio elongatus|YRFIR1741-23
Microphysogobio elongatus|YRFIR1744-23
Microphysogobio elongatus|YRFIR1745-23
Microphysogobio amurensis|YRFIR1742-23
Microphysogobio amurensis|YRFIR1739-23
Microphysogobio amurensis|YRFIR1743-23
Microphysogobio amurensis|YRFIR1746-23
Microphysogobio amurensis|YRFIR1747-23
Microphysogobio amurensis|YRFIR1748-23
Gobio coriparoides|YRFIR1417-23
Gobio coriparoides|YRFIR1454-23
Gobio coriparoides|YRFIR1456-23
Gobio coriparoides|YRFIR1448-23
Gobio coriparoides|YRFIR1453-23
Gobio coriparoides|YRFIR1421-23
Gobio coriparoides|YRFIR1455-23
Gobio coriparoides|YRFIR1457-23
Gobio coriparoides|YRFIR1407-23
Gobio coriparoides|YRFIR1423-23
Gobio coriparoides|YRFIR2925-23
Gobio coriparoides|YRFIR1412-23
Gobio coriparoides|YRFIR1393-23
Gobio coriparoides|YRFIR1394-23
Gobio coriparoides|YRFIR1395-23
Gobio coriparoides|YRFIR1396-23
Gobio coriparoides|YRFIR1398-23
Gobio coriparoides|YRFIR1399-23
Gobio coriparoides|YRFIR1400-23
Gobio coriparoides|YRFIR1401-23
Gobio coriparoides|YRFIR1402-23
Gobio coriparoides|YRFIR1404-23

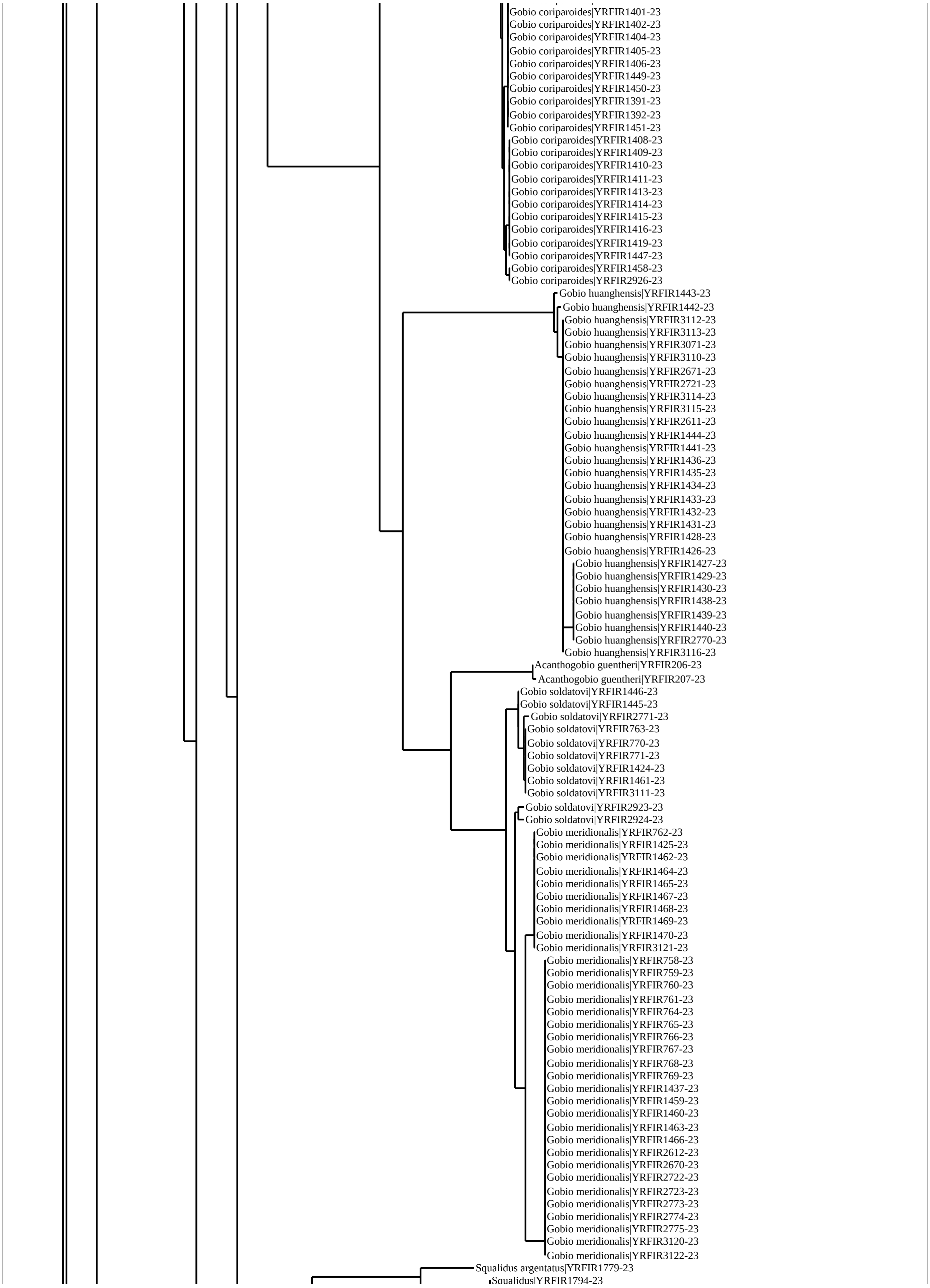
Gobio coriparoides|YRFIR1401-23
Gobio coriparoides|YRFIR1402-23
Gobio coriparoides|YRFIR1404-23
Gobio coriparoides|YRFIR1405-23
Gobio coriparoides|YRFIR1406-23
Gobio coriparoides|YRFIR1449-23
Gobio coriparoides|YRFIR1450-23
Gobio coriparoides|YRFIR1391-23
Gobio coriparoides|YRFIR1392-23
Gobio coriparoides|YRFIR1451-23
Gobio coriparoides|YRFIR1408-23
Gobio coriparoides|YRFIR1409-23
Gobio coriparoides|YRFIR1410-23
Gobio coriparoides|YRFIR1411-23
Gobio coriparoides|YRFIR1413-23
Gobio coriparoides|YRFIR1414-23
Gobio coriparoides|YRFIR1415-23
Gobio coriparoides|YRFIR1416-23
Gobio coriparoides|YRFIR1419-23
Gobio coriparoides|YRFIR1447-23
Gobio coriparoides|YRFIR1458-23
Gobio coriparoides|YRFIR2926-23
Gobio huanghensis|YRFIR1443-23
Gobio huanghensis|YRFIR1442-23
Gobio huanghensis|YRFIR3112-23
Gobio huanghensis|YRFIR3113-23
Gobio huanghensis|YRFIR3071-23
Gobio huanghensis|YRFIR3110-23
Gobio huanghensis|YRFIR2671-23
Gobio huanghensis|YRFIR2721-23
Gobio huanghensis|YRFIR3114-23
Gobio huanghensis|YRFIR3115-23
Gobio huanghensis|YRFIR2611-23
Gobio huanghensis|YRFIR1444-23
Gobio huanghensis|YRFIR1441-23
Gobio huanghensis|YRFIR1436-23
Gobio huanghensis|YRFIR1435-23
Gobio huanghensis|YRFIR1434-23
Gobio huanghensis|YRFIR1433-23
Gobio huanghensis|YRFIR1432-23
Gobio huanghensis|YRFIR1431-23
Gobio huanghensis|YRFIR1428-23
Gobio huanghensis|YRFIR1426-23
Gobio huanghensis|YRFIR1427-23
Gobio huanghensis|YRFIR1429-23
Gobio huanghensis|YRFIR1430-23
Gobio huanghensis|YRFIR1438-23
Gobio huanghensis|YRFIR1439-23
Gobio huanghensis|YRFIR1440-23
Gobio huanghensis|YRFIR2770-23
Gobio huanghensis|YRFIR3116-23
Acanthogobio guentheri|YRFIR206-23
Acanthogobio guentheri|YRFIR207-23
Gobio soldatovi|YRFIR1446-23
Gobio soldatovi|YRFIR1445-23
Gobio soldatovi|YRFIR2771-23
Gobio soldatovi|YRFIR763-23
Gobio soldatovi|YRFIR770-23
Gobio soldatovi|YRFIR771-23
Gobio soldatovi|YRFIR1424-23
Gobio soldatovi|YRFIR1461-23
Gobio soldatovi|YRFIR3111-23
Gobio soldatovi|YRFIR2923-23
Gobio soldatovi|YRFIR2924-23
Gobio meridionalis|YRFIR762-23
Gobio meridionalis|YRFIR1425-23
Gobio meridionalis|YRFIR1462-23
Gobio meridionalis|YRFIR1464-23
Gobio meridionalis|YRFIR1465-23
Gobio meridionalis|YRFIR1467-23
Gobio meridionalis|YRFIR1468-23
Gobio meridionalis|YRFIR1469-23
Gobio meridionalis|YRFIR1470-23
Gobio meridionalis|YRFIR3121-23
Gobio meridionalis|YRFIR758-23
Gobio meridionalis|YRFIR759-23
Gobio meridionalis|YRFIR760-23
Gobio meridionalis|YRFIR761-23
Gobio meridionalis|YRFIR764-23
Gobio meridionalis|YRFIR765-23
Gobio meridionalis|YRFIR766-23
Gobio meridionalis|YRFIR767-23
Gobio meridionalis|YRFIR768-23
Gobio meridionalis|YRFIR769-23
Gobio meridionalis|YRFIR1437-23
Gobio meridionalis|YRFIR1459-23
Gobio meridionalis|YRFIR1460-23
Gobio meridionalis|YRFIR1463-23
Gobio meridionalis|YRFIR1466-23
Gobio meridionalis|YRFIR2612-23
Gobio meridionalis|YRFIR2670-23
Gobio meridionalis|YRFIR2722-23
Gobio meridionalis|YRFIR2723-23
Gobio meridionalis|YRFIR2773-23
Gobio meridionalis|YRFIR2774-23
Gobio meridionalis|YRFIR2775-23
Gobio meridionalis|YRFIR3120-23
Gobio meridionalis|YRFIR3122-23
Squalidus argentatus|YRFIR1779-23
Squalidus|YRFIR1794-23

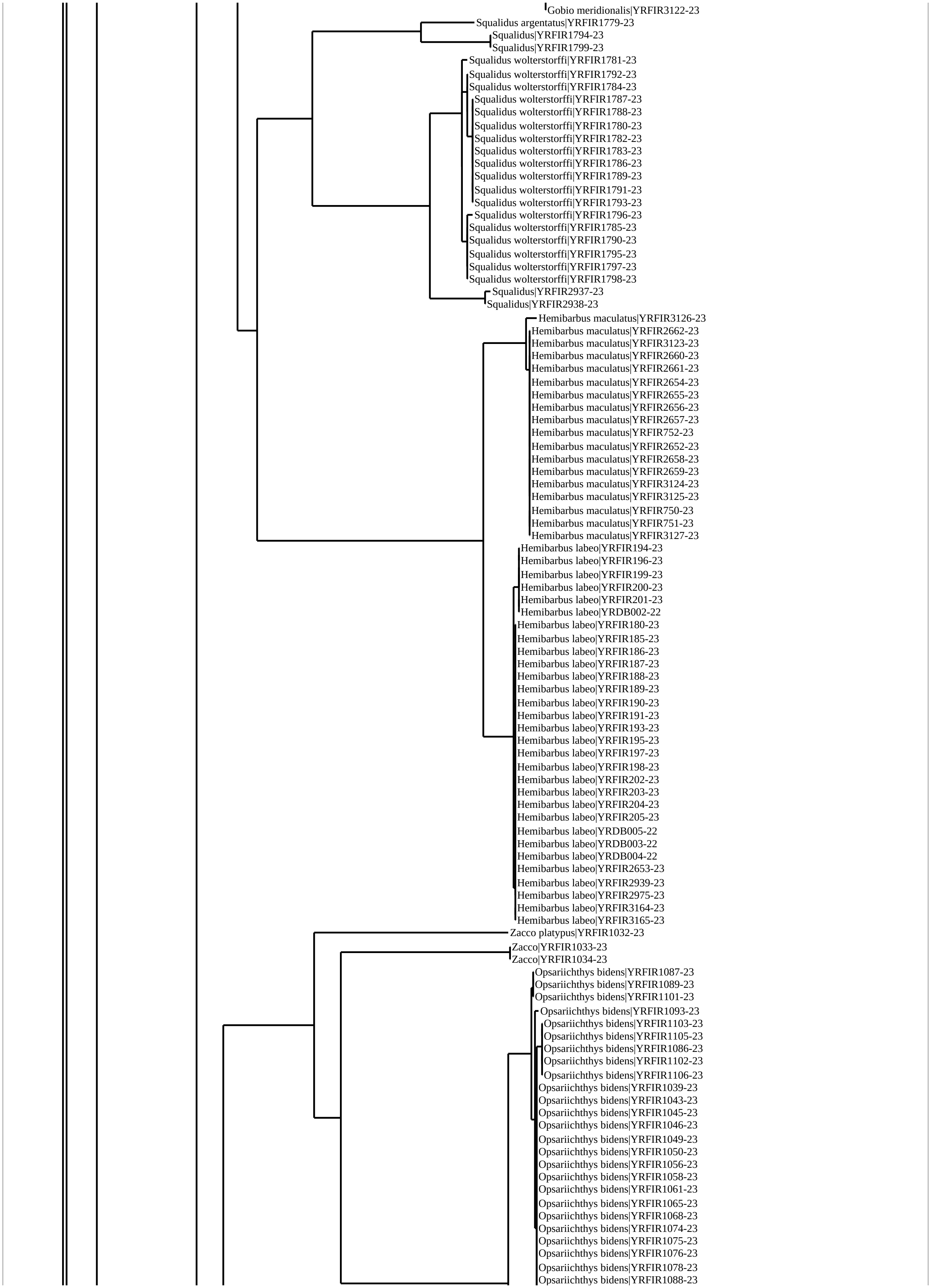
Gobio meridionalis|YRFIR3122-23
Squalidus argentatus|YRFIR1779-23
Squalidus|YRFIR1794-23
Squalidus|YRFIR1799-23
Squalidus wolterstorffi|YRFIR1781-23
Squalidus wolterstorffi|YRFIR1792-23
Squalidus wolterstorffi|YRFIR1784-23
Squalidus wolterstorffi|YRFIR1787-23
Squalidus wolterstorffi|YRFIR1788-23
Squalidus wolterstorffi|YRFIR1780-23
Squalidus wolterstorffi|YRFIR1782-23
Squalidus wolterstorffi|YRFIR1783-23
Squalidus wolterstorffi|YRFIR1786-23
Squalidus wolterstorffi|YRFIR1789-23
Squalidus wolterstorffi|YRFIR1791-23
Squalidus wolterstorffi|YRFIR1793-23
Squalidus wolterstorffi|YRFIR1796-23
Squalidus wolterstorffi|YRFIR1785-23
Squalidus wolterstorffi|YRFIR1790-23
Squalidus wolterstorffi|YRFIR1795-23
Squalidus wolterstorffi|YRFIR1797-23
Squalidus wolterstorffi|YRFIR1798-23
Squalidus|YRFIR2937-23
Squalidus|YRFIR2938-23
Hemibarbus maculatus|YRFIR3126-23
Hemibarbus maculatus|YRFIR2662-23
Hemibarbus maculatus|YRFIR3123-23
Hemibarbus maculatus|YRFIR2660-23
Hemibarbus maculatus|YRFIR2661-23
Hemibarbus maculatus|YRFIR2654-23
Hemibarbus maculatus|YRFIR2655-23
Hemibarbus maculatus|YRFIR2656-23
Hemibarbus maculatus|YRFIR2657-23
Hemibarbus maculatus|YRFIR752-23
Hemibarbus maculatus|YRFIR2652-23
Hemibarbus maculatus|YRFIR2658-23
Hemibarbus maculatus|YRFIR2659-23
Hemibarbus maculatus|YRFIR3124-23
Hemibarbus maculatus|YRFIR3125-23
Hemibarbus maculatus|YRFIR750-23
Hemibarbus maculatus|YRFIR751-23
Hemibarbus maculatus|YRFIR3127-23
Hemibarbus labeo|YRFIR194-23
Hemibarbus labeo|YRFIR196-23
Hemibarbus labeo|YRFIR199-23
Hemibarbus labeo|YRFIR200-23
Hemibarbus labeo|YRFIR201-23
Hemibarbus labeo|YRDB002-22
Hemibarbus labeo|YRFIR180-23
Hemibarbus labeo|YRFIR185-23
Hemibarbus labeo|YRFIR186-23
Hemibarbus labeo|YRFIR187-23
Hemibarbus labeo|YRFIR188-23
Hemibarbus labeo|YRFIR189-23
Hemibarbus labeo|YRFIR190-23
Hemibarbus labeo|YRFIR191-23
Hemibarbus labeo|YRFIR193-23
Hemibarbus labeo|YRFIR195-23
Hemibarbus labeo|YRFIR197-23
Hemibarbus labeo|YRFIR198-23
Hemibarbus labeo|YRFIR202-23
Hemibarbus labeo|YRFIR203-23
Hemibarbus labeo|YRFIR204-23
Hemibarbus labeo|YRFIR205-23
Hemibarbus labeo|YRDB005-22
Hemibarbus labeo|YRDB003-22
Hemibarbus labeo|YRDB004-22
Hemibarbus labeo|YRFIR2653-23
Hemibarbus labeo|YRFIR2939-23
Hemibarbus labeo|YRFIR2975-23
Hemibarbus labeo|YRFIR3164-23
Hemibarbus labeo|YRFIR3165-23
Zacco platypus|YRFIR1032-23
Zacco|YRFIR1033-23
Zacco|YRFIR1034-23
Opsariichthys bidens|YRFIR1087-23
Opsariichthys bidens|YRFIR1089-23
Opsariichthys bidens|YRFIR1101-23
Opsariichthys bidens|YRFIR1093-23
Opsariichthys bidens|YRFIR1103-23
Opsariichthys bidens|YRFIR1105-23
Opsariichthys bidens|YRFIR1086-23
Opsariichthys bidens|YRFIR1102-23
Opsariichthys bidens|YRFIR1106-23
Opsariichthys bidens|YRFIR1039-23
Opsariichthys bidens|YRFIR1043-23
Opsariichthys bidens|YRFIR1045-23
Opsariichthys bidens|YRFIR1046-23
Opsariichthys bidens|YRFIR1049-23
Opsariichthys bidens|YRFIR1050-23
Opsariichthys bidens|YRFIR1056-23
Opsariichthys bidens|YRFIR1058-23
Opsariichthys bidens|YRFIR1061-23
Opsariichthys bidens|YRFIR1065-23
Opsariichthys bidens|YRFIR1068-23
Opsariichthys bidens|YRFIR1074-23
Opsariichthys bidens|YRFIR1075-23
Opsariichthys bidens|YRFIR1076-23
Opsariichthys bidens|YRFIR1078-23
Opsariichthys bidens|YRFIR1088-23

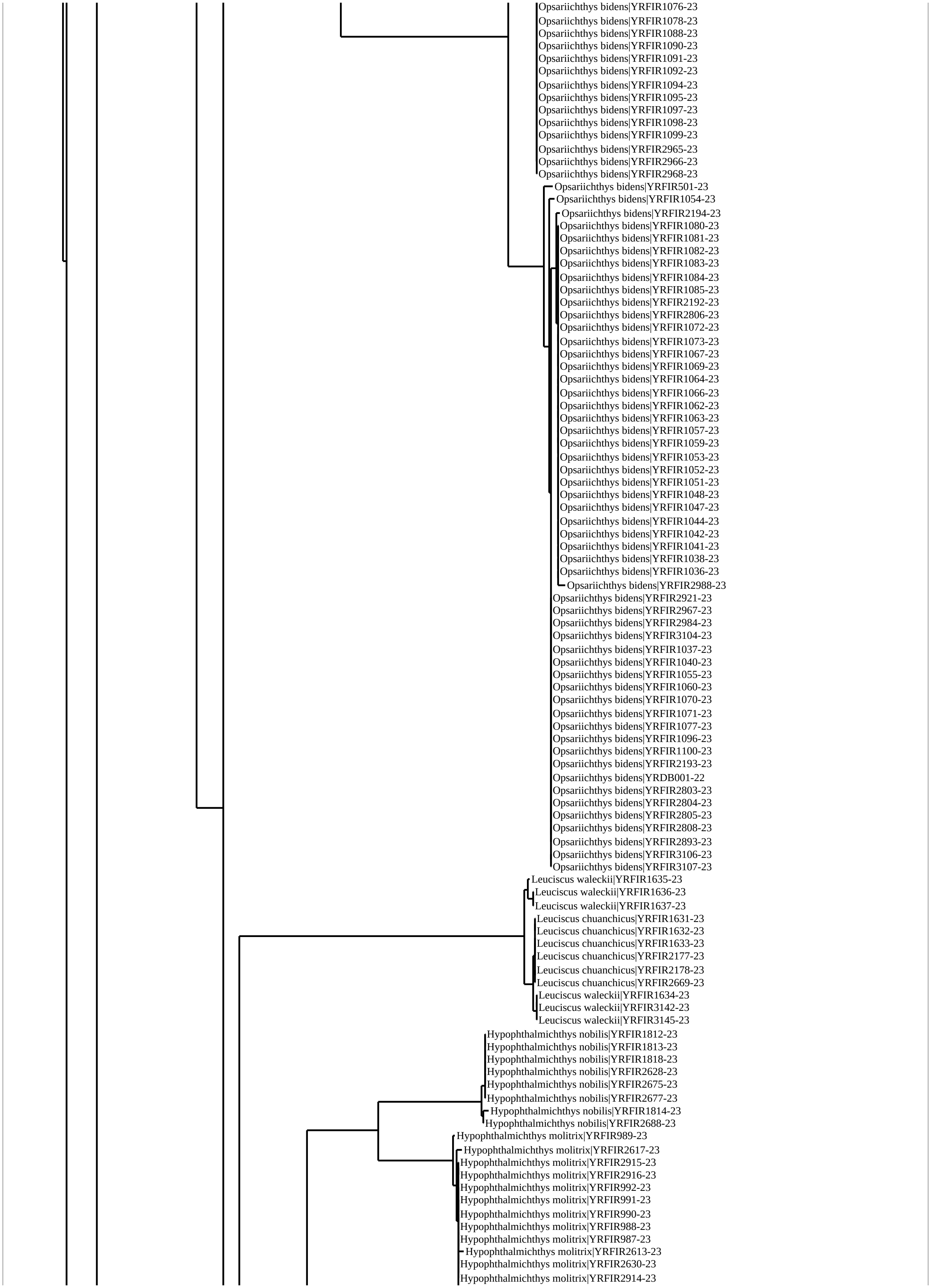
Opsariichthys bidens|YRFIR1076-23
Opsariichthys bidens|YRFIR1078-23
Opsariichthys bidens|YRFIR1088-23
Opsariichthys bidens|YRFIR1090-23
Opsariichthys bidens|YRFIR1091-23
Opsariichthys bidens|YRFIR1092-23
Opsariichthys bidens|YRFIR1094-23
Opsariichthys bidens|YRFIR1095-23
Opsariichthys bidens|YRFIR1097-23
Opsariichthys bidens|YRFIR1098-23
Opsariichthys bidens|YRFIR1099-23
Opsariichthys bidens|YRFIR2965-23
Opsariichthys bidens|YRFIR2966-23
Opsariichthys bidens|YRFIR2968-23
Opsariichthys bidens|YRFIR501-23
Opsariichthys bidens|YRFIR1054-23
Opsariichthys bidens|YRFIR2194-23
Opsariichthys bidens|YRFIR1080-23
Opsariichthys bidens|YRFIR1081-23
Opsariichthys bidens|YRFIR1082-23
Opsariichthys bidens|YRFIR1083-23
Opsariichthys bidens|YRFIR1084-23
Opsariichthys bidens|YRFIR1085-23
Opsariichthys bidens|YRFIR2192-23
Opsariichthys bidens|YRFIR2806-23
Opsariichthys bidens|YRFIR1072-23
Opsariichthys bidens|YRFIR1073-23
Opsariichthys bidens|YRFIR1067-23
Opsariichthys bidens|YRFIR1069-23
Opsariichthys bidens|YRFIR1064-23
Opsariichthys bidens|YRFIR1066-23
Opsariichthys bidens|YRFIR1062-23
Opsariichthys bidens|YRFIR1063-23
Opsariichthys bidens|YRFIR1057-23
Opsariichthys bidens|YRFIR1059-23
Opsariichthys bidens|YRFIR1053-23
Opsariichthys bidens|YRFIR1052-23
Opsariichthys bidens|YRFIR1051-23
Opsariichthys bidens|YRFIR1048-23
Opsariichthys bidens|YRFIR1047-23
Opsariichthys bidens|YRFIR1044-23
Opsariichthys bidens|YRFIR1042-23
Opsariichthys bidens|YRFIR1041-23
Opsariichthys bidens|YRFIR1038-23
Opsariichthys bidens|YRFIR1036-23
Opsariichthys bidens|YRFIR2988-23
Opsariichthys bidens|YRFIR2921-23
Opsariichthys bidens|YRFIR2967-23
Opsariichthys bidens|YRFIR2984-23
Opsariichthys bidens|YRFIR3104-23
Opsariichthys bidens|YRFIR1037-23
Opsariichthys bidens|YRFIR1040-23
Opsariichthys bidens|YRFIR1055-23
Opsariichthys bidens|YRFIR1060-23
Opsariichthys bidens|YRFIR1070-23
Opsariichthys bidens|YRFIR1071-23
Opsariichthys bidens|YRFIR1077-23
Opsariichthys bidens|YRFIR1096-23
Opsariichthys bidens|YRFIR1100-23
Opsariichthys bidens|YRFIR2193-23
Opsariichthys bidens|YRDB001-22
Opsariichthys bidens|YRFIR2803-23
Opsariichthys bidens|YRFIR2804-23
Opsariichthys bidens|YRFIR2805-23
Opsariichthys bidens|YRFIR2808-23
Opsariichthys bidens|YRFIR2893-23
Opsariichthys bidens|YRFIR3106-23
Opsariichthys bidens|YRFIR3107-23
Leuciscus waleckii|YRFIR1635-23
Leuciscus waleckii|YRFIR1636-23
Leuciscus waleckii|YRFIR1637-23
Leuciscus chuanchicus|YRFIR1631-23
Leuciscus chuanchicus|YRFIR1632-23
Leuciscus chuanchicus|YRFIR1633-23
Leuciscus chuanchicus|YRFIR2177-23
Leuciscus chuanchicus|YRFIR2178-23
Leuciscus chuanchicus|YRFIR2669-23
Leuciscus waleckii|YRFIR1634-23
Leuciscus waleckii|YRFIR3142-23
Leuciscus waleckii|YRFIR3145-23
Hypophthalmichthys nobilis|YRFIR1812-23
Hypophthalmichthys nobilis|YRFIR1813-23
Hypophthalmichthys nobilis|YRFIR1818-23
Hypophthalmichthys nobilis|YRFIR2628-23
Hypophthalmichthys nobilis|YRFIR2675-23
Hypophthalmichthys nobilis|YRFIR2677-23
Hypophthalmichthys nobilis|YRFIR1814-23
Hypophthalmichthys nobilis|YRFIR2688-23
Hypophthalmichthys molitrix|YRFIR989-23
Hypophthalmichthys molitrix|YRFIR2617-23
Hypophthalmichthys molitrix|YRFIR2915-23
Hypophthalmichthys molitrix|YRFIR2916-23
Hypophthalmichthys molitrix|YRFIR992-23
Hypophthalmichthys molitrix|YRFIR991-23
Hypophthalmichthys molitrix|YRFIR990-23
Hypophthalmichthys molitrix|YRFIR988-23
Hypophthalmichthys molitrix|YRFIR987-23
Hypophthalmichthys molitrix|YRFIR2613-23
Hypophthalmichthys molitrix|YRFIR2630-23
Hypophthalmichthys molitrix|YRFIR2914-23

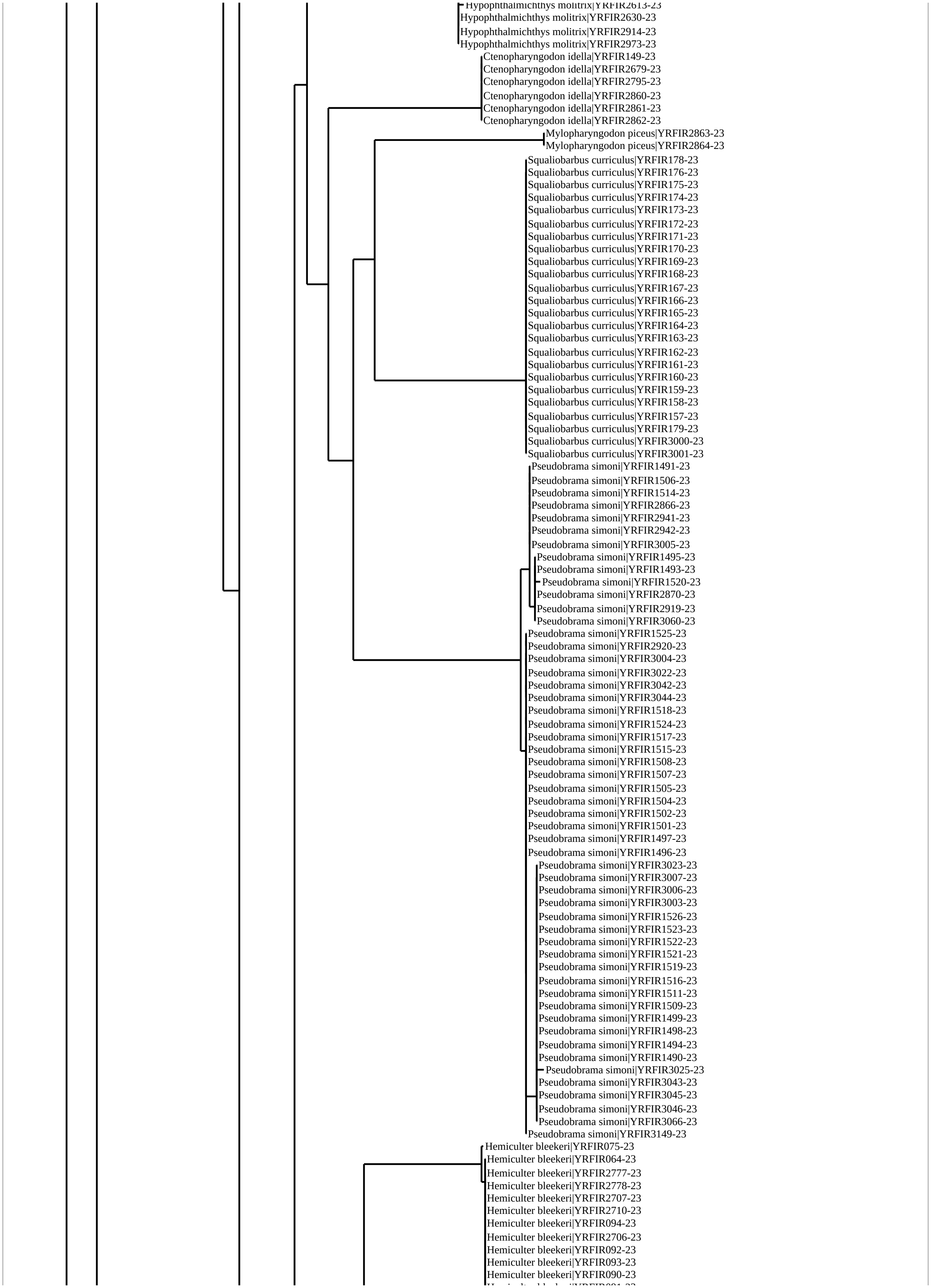
Hypophthalmichthys molitrix|YRFIR2613-23
Hypophthalmichthys molitrix|YRFIR2630-23
Hypophthalmichthys molitrix|YRFIR2914-23
Hypophthalmichthys molitrix|YRFIR2973-23
Ctenopharyngodon idella|YRFIR149-23
Ctenopharyngodon idella|YRFIR2679-23
Ctenopharyngodon idella|YRFIR2795-23
Ctenopharyngodon idella|YRFIR2860-23
Ctenopharyngodon idella|YRFIR2861-23
Ctenopharyngodon idella|YRFIR2862-23
Mylopharyngodon piceus|YRFIR2863-23
Mylopharyngodon piceus|YRFIR2864-23
Squaliobarbus curriculus|YRFIR178-23
Squaliobarbus curriculus|YRFIR176-23
Squaliobarbus curriculus|YRFIR175-23
Squaliobarbus curriculus|YRFIR174-23
Squaliobarbus curriculus|YRFIR173-23
Squaliobarbus curriculus|YRFIR172-23
Squaliobarbus curriculus|YRFIR171-23
Squaliobarbus curriculus|YRFIR170-23
Squaliobarbus curriculus|YRFIR169-23
Squaliobarbus curriculus|YRFIR168-23
Squaliobarbus curriculus|YRFIR167-23
Squaliobarbus curriculus|YRFIR166-23
Squaliobarbus curriculus|YRFIR165-23
Squaliobarbus curriculus|YRFIR164-23
Squaliobarbus curriculus|YRFIR163-23
Squaliobarbus curriculus|YRFIR162-23
Squaliobarbus curriculus|YRFIR161-23
Squaliobarbus curriculus|YRFIR160-23
Squaliobarbus curriculus|YRFIR159-23
Squaliobarbus curriculus|YRFIR158-23
Squaliobarbus curriculus|YRFIR157-23
Squaliobarbus curriculus|YRFIR179-23
Squaliobarbus curriculus|YRFIR3000-23
Squaliobarbus curriculus|YRFIR3001-23
Pseudobrama simoni|YRFIR1491-23
Pseudobrama simoni|YRFIR1506-23
Pseudobrama simoni|YRFIR1514-23
Pseudobrama simoni|YRFIR2866-23
Pseudobrama simoni|YRFIR2941-23
Pseudobrama simoni|YRFIR2942-23
Pseudobrama simoni|YRFIR3005-23
Pseudobrama simoni|YRFIR1495-23
Pseudobrama simoni|YRFIR1493-23
Pseudobrama simoni|YRFIR1520-23
Pseudobrama simoni|YRFIR2870-23
Pseudobrama simoni|YRFIR2919-23
Pseudobrama simoni|YRFIR3060-23
Pseudobrama simoni|YRFIR1525-23
Pseudobrama simoni|YRFIR2920-23
Pseudobrama simoni|YRFIR3004-23
Pseudobrama simoni|YRFIR3022-23
Pseudobrama simoni|YRFIR3042-23
Pseudobrama simoni|YRFIR3044-23
Pseudobrama simoni|YRFIR1518-23
Pseudobrama simoni|YRFIR1524-23
Pseudobrama simoni|YRFIR1517-23
Pseudobrama simoni|YRFIR1515-23
Pseudobrama simoni|YRFIR1508-23
Pseudobrama simoni|YRFIR1507-23
Pseudobrama simoni|YRFIR1505-23
Pseudobrama simoni|YRFIR1504-23
Pseudobrama simoni|YRFIR1502-23
Pseudobrama simoni|YRFIR1501-23
Pseudobrama simoni|YRFIR1497-23
Pseudobrama simoni|YRFIR1496-23
Pseudobrama simoni|YRFIR3023-23
Pseudobrama simoni|YRFIR3007-23
Pseudobrama simoni|YRFIR3006-23
Pseudobrama simoni|YRFIR3003-23
Pseudobrama simoni|YRFIR1526-23
Pseudobrama simoni|YRFIR1523-23
Pseudobrama simoni|YRFIR1522-23
Pseudobrama simoni|YRFIR1521-23
Pseudobrama simoni|YRFIR1519-23
Pseudobrama simoni|YRFIR1516-23
Pseudobrama simoni|YRFIR1511-23
Pseudobrama simoni|YRFIR1509-23
Pseudobrama simoni|YRFIR1499-23
Pseudobrama simoni|YRFIR1498-23
Pseudobrama simoni|YRFIR1494-23
Pseudobrama simoni|YRFIR1490-23
Pseudobrama simoni|YRFIR3025-23
Pseudobrama simoni|YRFIR3043-23
Pseudobrama simoni|YRFIR3045-23
Pseudobrama simoni|YRFIR3046-23
Pseudobrama simoni|YRFIR3066-23
Pseudobrama simoni|YRFIR3149-23
Hemiculter bleekeri|YRFIR075-23
Hemiculter bleekeri|YRFIR064-23
Hemiculter bleekeri|YRFIR2777-23
Hemiculter bleekeri|YRFIR2778-23
Hemiculter bleekeri|YRFIR2707-23
Hemiculter bleekeri|YRFIR2710-23
Hemiculter bleekeri|YRFIR094-23
Hemiculter bleekeri|YRFIR2706-23
Hemiculter bleekeri|YRFIR092-23
Hemiculter bleekeri|YRFIR093-23
Hemiculter bleekeri|YRFIR090-23

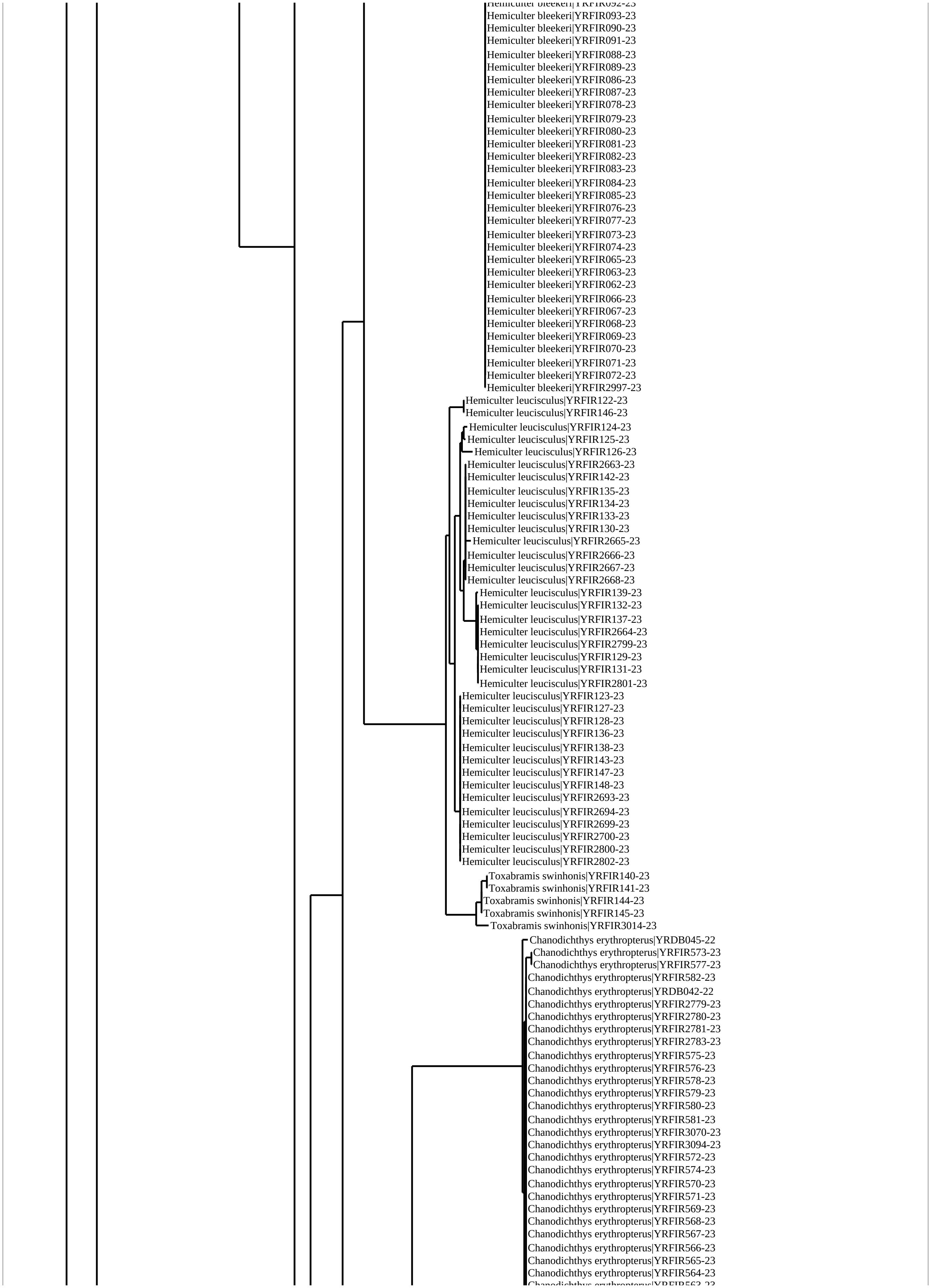
Hemiculter bleekeri|YRFIR092-23
Hemiculter bleekeri|YRFIR093-23
Hemiculter bleekeri|YRFIR090-23
Hemiculter bleekeri|YRFIR091-23
Hemiculter bleekeri|YRFIR088-23
Hemiculter bleekeri|YRFIR089-23
Hemiculter bleekeri|YRFIR086-23
Hemiculter bleekeri|YRFIR087-23
Hemiculter bleekeri|YRFIR078-23
Hemiculter bleekeri|YRFIR079-23
Hemiculter bleekeri|YRFIR080-23
Hemiculter bleekeri|YRFIR081-23
Hemiculter bleekeri|YRFIR082-23
Hemiculter bleekeri|YRFIR083-23
Hemiculter bleekeri|YRFIR084-23
Hemiculter bleekeri|YRFIR085-23
Hemiculter bleekeri|YRFIR076-23
Hemiculter bleekeri|YRFIR077-23
Hemiculter bleekeri|YRFIR073-23
Hemiculter bleekeri|YRFIR074-23
Hemiculter bleekeri|YRFIR065-23
Hemiculter bleekeri|YRFIR063-23
Hemiculter bleekeri|YRFIR062-23
Hemiculter bleekeri|YRFIR066-23
Hemiculter bleekeri|YRFIR067-23
Hemiculter bleekeri|YRFIR068-23
Hemiculter bleekeri|YRFIR069-23
Hemiculter bleekeri|YRFIR070-23
Hemiculter bleekeri|YRFIR071-23
Hemiculter bleekeri|YRFIR072-23
Hemiculter bleekeri|YRFIR2997-23
Hemiculter leucisculus|YRFIR122-23
Hemiculter leucisculus|YRFIR146-23
Hemiculter leucisculus|YRFIR124-23
Hemiculter leucisculus|YRFIR125-23
Hemiculter leucisculus|YRFIR126-23
Hemiculter leucisculus|YRFIR2663-23
Hemiculter leucisculus|YRFIR142-23
Hemiculter leucisculus|YRFIR135-23
Hemiculter leucisculus|YRFIR134-23
Hemiculter leucisculus|YRFIR133-23
Hemiculter leucisculus|YRFIR130-23
Hemiculter leucisculus|YRFIR2665-23
Hemiculter leucisculus|YRFIR2666-23
Hemiculter leucisculus|YRFIR2667-23
Hemiculter leucisculus|YRFIR2668-23
Hemiculter leucisculus|YRFIR139-23
Hemiculter leucisculus|YRFIR132-23
Hemiculter leucisculus|YRFIR137-23
Hemiculter leucisculus|YRFIR2664-23
Hemiculter leucisculus|YRFIR2799-23
Hemiculter leucisculus|YRFIR129-23
Hemiculter leucisculus|YRFIR131-23
Hemiculter leucisculus|YRFIR2801-23
Hemiculter leucisculus|YRFIR123-23
Hemiculter leucisculus|YRFIR127-23
Hemiculter leucisculus|YRFIR128-23
Hemiculter leucisculus|YRFIR136-23
Hemiculter leucisculus|YRFIR138-23
Hemiculter leucisculus|YRFIR143-23
Hemiculter leucisculus|YRFIR147-23
Hemiculter leucisculus|YRFIR148-23
Hemiculter leucisculus|YRFIR2693-23
Hemiculter leucisculus|YRFIR2694-23
Hemiculter leucisculus|YRFIR2699-23
Hemiculter leucisculus|YRFIR2700-23
Hemiculter leucisculus|YRFIR2800-23
Hemiculter leucisculus|YRFIR2802-23
Toxabramis swinhonis|YRFIR140-23
Toxabramis swinhonis|YRFIR141-23
Toxabramis swinhonis|YRFIR144-23
Toxabramis swinhonis|YRFIR145-23
Toxabramis swinhonis|YRFIR3014-23
Chanodichthys erythropterus|YRDB045-22
Chanodichthys erythropterus|YRFIR573-23
Chanodichthys erythropterus|YRFIR577-23
Chanodichthys erythropterus|YRFIR582-23
Chanodichthys erythropterus|YRDB042-22
Chanodichthys erythropterus|YRFIR2779-23
Chanodichthys erythropterus|YRFIR2780-23
Chanodichthys erythropterus|YRFIR2781-23
Chanodichthys erythropterus|YRFIR2783-23
Chanodichthys erythropterus|YRFIR575-23
Chanodichthys erythropterus|YRFIR576-23
Chanodichthys erythropterus|YRFIR578-23
Chanodichthys erythropterus|YRFIR579-23
Chanodichthys erythropterus|YRFIR580-23
Chanodichthys erythropterus|YRFIR581-23
Chanodichthys erythropterus|YRFIR3070-23
Chanodichthys erythropterus|YRFIR3094-23
Chanodichthys erythropterus|YRFIR572-23
Chanodichthys erythropterus|YRFIR574-23
Chanodichthys erythropterus|YRFIR570-23
Chanodichthys erythropterus|YRFIR571-23
Chanodichthys erythropterus|YRFIR569-23
Chanodichthys erythropterus|YRFIR568-23
Chanodichthys erythropterus|YRFIR567-23
Chanodichthys erythropterus|YRFIR566-23
Chanodichthys erythropterus|YRFIR565-23
Chanodichthys erythropterus|YRFIR564-23
Chanodichthys erythropterus|YRFIR563-23

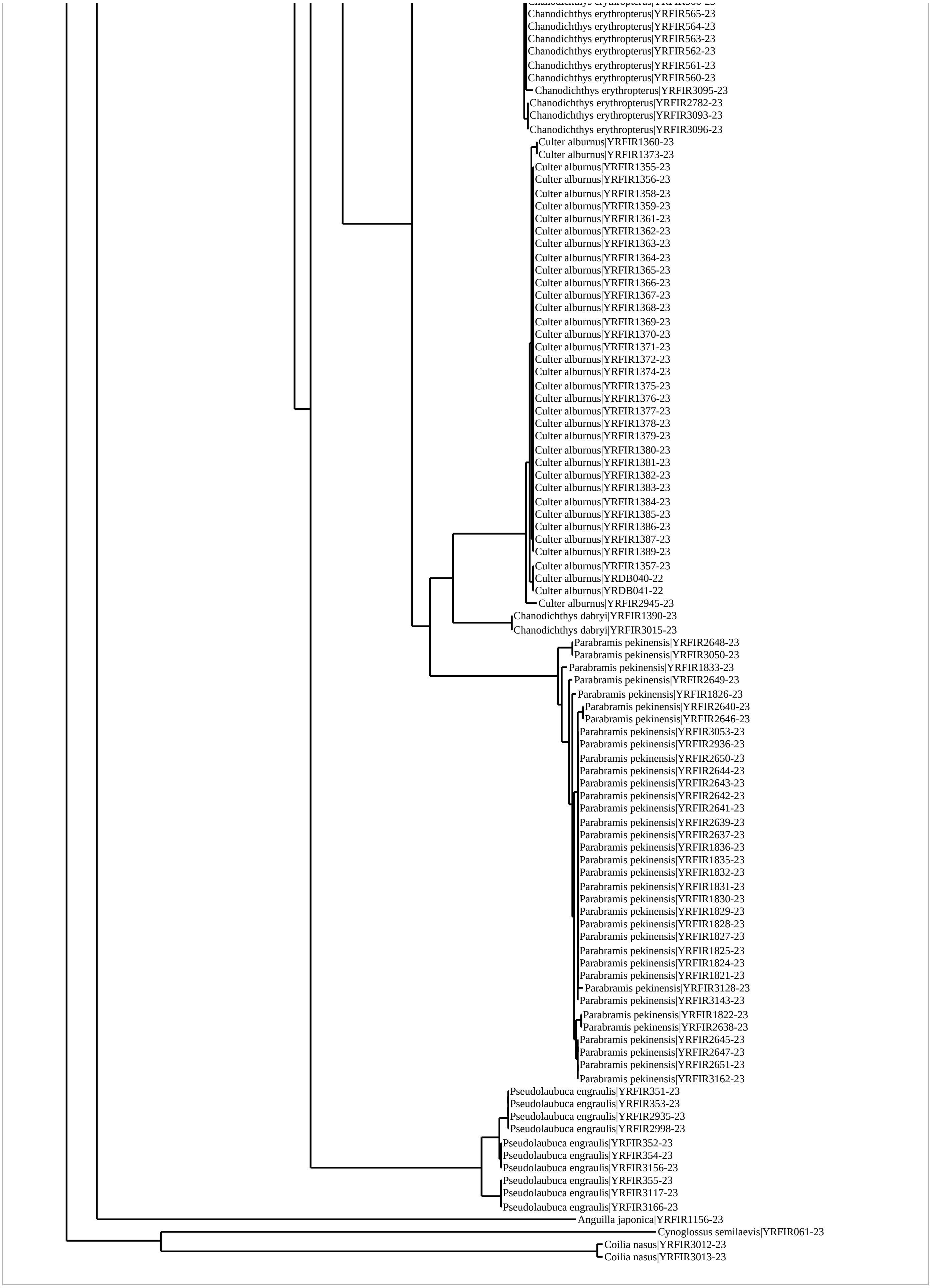
Chanodichthys erythropterus|YRFIR565-23
Chanodichthys erythropterus|YRFIR564-23
Chanodichthys erythropterus|YRFIR563-23
Chanodichthys erythropterus|YRFIR562-23
Chanodichthys erythropterus|YRFIR561-23
Chanodichthys erythropterus|YRFIR560-23
Chanodichthys erythropterus|YRFIR3095-23
Chanodichthys erythropterus|YRFIR2782-23
Chanodichthys erythropterus|YRFIR3093-23
Chanodichthys erythropterus|YRFIR3096-23
Culter alburnus|YRFIR1360-23
Culter alburnus|YRFIR1373-23
Culter alburnus|YRFIR1355-23
Culter alburnus|YRFIR1356-23
Culter alburnus|YRFIR1358-23
Culter alburnus|YRFIR1359-23
Culter alburnus|YRFIR1361-23
Culter alburnus|YRFIR1362-23
Culter alburnus|YRFIR1363-23
Culter alburnus|YRFIR1364-23
Culter alburnus|YRFIR1365-23
Culter alburnus|YRFIR1366-23
Culter alburnus|YRFIR1367-23
Culter alburnus|YRFIR1368-23
Culter alburnus|YRFIR1369-23
Culter alburnus|YRFIR1370-23
Culter alburnus|YRFIR1371-23
Culter alburnus|YRFIR1372-23
Culter alburnus|YRFIR1374-23
Culter alburnus|YRFIR1375-23
Culter alburnus|YRFIR1376-23
Culter alburnus|YRFIR1377-23
Culter alburnus|YRFIR1378-23
Culter alburnus|YRFIR1379-23
Culter alburnus|YRFIR1380-23
Culter alburnus|YRFIR1381-23
Culter alburnus|YRFIR1382-23
Culter alburnus|YRFIR1383-23
Culter alburnus|YRFIR1384-23
Culter alburnus|YRFIR1385-23
Culter alburnus|YRFIR1386-23
Culter alburnus|YRFIR1387-23
Culter alburnus|YRFIR1389-23
Culter alburnus|YRFIR1357-23
Culter alburnus|YRDB040-22
Culter alburnus|YRDB041-22
Culter alburnus|YRFIR2945-23
Chanodichthys dabryi|YRFIR1390-23
Chanodichthys dabryi|YRFIR3015-23
Parabramis pekinensis|YRFIR2648-23
Parabramis pekinensis|YRFIR3050-23
Parabramis pekinensis|YRFIR1833-23
Parabramis pekinensis|YRFIR2649-23
Parabramis pekinensis|YRFIR1826-23
Parabramis pekinensis|YRFIR2640-23
Parabramis pekinensis|YRFIR2646-23
Parabramis pekinensis|YRFIR3053-23
Parabramis pekinensis|YRFIR2936-23
Parabramis pekinensis|YRFIR2650-23
Parabramis pekinensis|YRFIR2644-23
Parabramis pekinensis|YRFIR2643-23
Parabramis pekinensis|YRFIR2642-23
Parabramis pekinensis|YRFIR2641-23
Parabramis pekinensis|YRFIR2639-23
Parabramis pekinensis|YRFIR2637-23
Parabramis pekinensis|YRFIR1836-23
Parabramis pekinensis|YRFIR1835-23
Parabramis pekinensis|YRFIR1832-23
Parabramis pekinensis|YRFIR1831-23
Parabramis pekinensis|YRFIR1830-23
Parabramis pekinensis|YRFIR1829-23
Parabramis pekinensis|YRFIR1828-23
Parabramis pekinensis|YRFIR1827-23
Parabramis pekinensis|YRFIR1825-23
Parabramis pekinensis|YRFIR1824-23
Parabramis pekinensis|YRFIR1821-23
Parabramis pekinensis|YRFIR3128-23
Parabramis pekinensis|YRFIR3143-23
Parabramis pekinensis|YRFIR1822-23
Parabramis pekinensis|YRFIR2638-23
Parabramis pekinensis|YRFIR2645-23
Parabramis pekinensis|YRFIR2647-23
Parabramis pekinensis|YRFIR2651-23
Parabramis pekinensis|YRFIR3162-23
Pseudolaubuca engraulis|YRFIR351-23
Pseudolaubuca engraulis|YRFIR353-23
Pseudolaubuca engraulis|YRFIR2935-23
Pseudolaubuca engraulis|YRFIR2998-23
Pseudolaubuca engraulis|YRFIR352-23
Pseudolaubuca engraulis|YRFIR354-23
Pseudolaubuca engraulis|YRFIR3156-23
Pseudolaubuca engraulis|YRFIR355-23
Pseudolaubuca engraulis|YRFIR3117-23
Pseudolaubuca engraulis|YRFIR3166-23
Anguilla japonica|YRFIR1156-23
Cynoglossus semilaevis|YRFIR061-23
Coilia nasus|YRFIR3012-23
Coilia nasus|YRFIR3013-23
